# Clinical Benefits and Safety of FMS-Like Tyrosine Kinase 3 Inhibitors in Various Treatment Stages of Acute Myeloid Leukemia: A Systematic Review, Meta-Analysis, and Network Meta-Analysis

**DOI:** 10.3389/fonc.2021.686013

**Published:** 2021-06-03

**Authors:** Qingyu Xu, Shujiao He, Li Yu

**Affiliations:** ^1^ Department of Hematology and Oncology, International Cancer Center, Shenzhen Key Laboratory, Shenzhen University General Hospital, Shenzhen University Clinical Medical Academy, Shenzhen University Health Science Center, Shenzhen, China; ^2^ Department of Hematology and Oncology, Medical Faculty Mannheim, Heidelberg University, Mannheim, Germany

**Keywords:** acute myeloid leukemia, FLT3 inhibitor, response, survival, toxicity, meta-analysis, network meta-analysis

## Abstract

**Background:**

Given the controversial roles of FMS-like tyrosine kinase 3 inhibitors (FLT3i) in various treatment stages of acute myeloid leukemia (AML), this study was designed to assess this problem and further explored which FLT3i worked more effectively.

**Methods:**

A systematic review, meta-analysis and network meta-analysis (NMA) were conducted by filtering PubMed, Embase, Cochrane library, and Chinese databases. We included studies comparing therapeutic effects between FLT3i and non-FLT3i group in AML, particularly *FLT3*(+) patients, or demonstrating the efficiency of allogeneic hematopoietic stem cell transplantation (allo-HSCT) in *FLT3*(+) AML. Relative risk (RR) with 95% confidence intervals (CI) was used for estimating complete remission (CR), early death and toxicity. Hazard ratio (HR) was used to assess overall survival (OS), event-free survival (EFS), relapse-free survival (RFS) and cumulative incidence of relapse (CIR).

**Results:**

After addressing all criteria, 39 studies were eventually analyzed. Better CR was accomplished by FLT3i in untreated AML (RR 0.88, *p* = 0.04) and refractory and relapsed *FLT3*(+) AML (rrAML) (RR 0.61, *p* < 0.01) compared to non-FLT3i arm, followed by improved survival (untreated AML: OS, HR 0.76; EFS, HR 0.67; RFS, HR 0.72; all *p* < 0.01; *FLT3*(+) rrAML: OS, HR 0.60, *p* < 0.01; RFS, HR 0.40, *p* = 0.01). In addition, allo-HSCT improved survival in *FLT3*(+) AML (OS, HR 0.53; EFS, HR 0.50; RFS, HR 0.57; CIR, HR 0.26; all *p* < 0.01), which was further prolonged by FLT3i administrated after allo-HSCT (OS, HR 0.45; RFS, HR 0.34; CIR, HR 0.32; all *p* < 0.01). Additionally, FLT3i consistently improved OS (p < 0.05) regardless of *FLT3-ITD* ratio, when compared to non-FLT3i group. Besides, FLT3i showed significantly increased risk of thrombocytopenia, neutropenia, anemia, skin- and cardiac-related adverse effects, increased alanine aminotransferase, and increased risk of cough and dyspnea (*p* < 0.05). In NMA, gilteritinib showed the highest probability for improved prognosis.

**Conclusions:**

FLT3i safely improved prognosis in induction/reinduction stage of *FLT3*(+) AML and further boosted survival benefits from allo-HSCT as maintenance therapy, suggesting better prognosis if FLT3i is combined before and after allo-HSCT. In NMA, gilteritinib potentially achieved the best prognosis, which should be identified in direct trials.

## Introduction

Acute myeloid leukemia (AML) is a heterogeneous hematologic malignancy characterized by a maturation block and accumulation of myeloid progenitor cells (1). Among the most prevalent AML genetic aberrations, FMS-like tyrosine kinase 3 (*FLT3*) mutations are detected in approximately one-third of patients (2), comprising about three-quarters of *FLT3*(+) patients with internal tandem duplication (*FLT3-ITD*) ranging from 3 to more than 100 amino acids located in the juxtamembrane region and a *FLT3* point mutation in the tyrosine kinase domain (*TKD*) in approximately 8% of newly diagnosed AML (3). *FLT3*-related mutations induce constitutive activation of *FLT3* receptor and trigger the downstream pathways resulting in leukemic cell proliferation, impaired differentiation, and resistance to apoptosis (4). *FLT3-ITD*(+) patients always manifest poor prognosis, distinguished by high resistance frequency to induction chemotherapy and relapse, decreased response to salvage therapy, and shorter survival. However, *TKD* influence on prognosis remains contradictory (2).

*FLT3* inhibitors (FLT3i) are a type of tyrosine kinase inhibitors with *FLT3* inhibitory activity that may be particularly utilized to treat *FLT3*(+) AML with likely improved prognosis. According to current clinical studies, they proved that AML patients benefited from FLT3i (3, 5–7). The following FLT3i are the most frequently estimated in phase 2 or 3 of randomized controlled trials (RCT) and retrospective studies: sorafenib, lestaurtinib, midostaurin, quizartinib and gilteritinib. Typically, sorafenib, lestaurtinib and midostaurin demonstrate activity against multi-kinase. In particular, sorafenib is the most common FLT3i for AML, with high activity against *ITD* mutations instead of wild-type *FLT3* and *TK*D mutations (8). Lestaurtinib is an indolocarbazole inhibitor, with equal inhibiting impact on *FLT3-ITD* and *-TKD* mutations (9, 10). Midostaurin is also a multi-targeted indolocarbazole, with equal activity against mutated *FLT3-ITD* and *-*TKD (3). Quizartinib is a FLT3i with potent activity against mutated *FLT3-ITD* and wild-type *FLT3*, but without intrinsic activity against *TKD* mutations (5, 9). Quizartinib can also moderately inhibit *KIT* (5). Gilteritinib is a highly selective inhibitor of *FLT3* and *AXL* receptor tyrosine kinases, with anti-leukemic activity against *ITD* and *TKD* mutations (6, 9) but with weak activity against *KIT* (6).

These FLT3i are used in various stages, including induction with/without consolidation therapy in newly diagnosed AML, maintenance treatment after allogeneic hematopoietic stem cell transplantation (allo-HSCT) and salvage therapy in refractory and relapsed AML (rrAML). Up to now, no extensive study has been found to comprehensively explore the role of FLT3i in various AML treatment stages and explore which FLT3i probably works the best. Herein, we conducted a meta-analysis in an attempt to clarify the clinical benefit and safety of FLT3i. Data from allo-HSCT in *FLT3*(+) patients were also summarized to observe the effects of FLT3i as the maintenance therapy after allo-HSCT. We further performed network meta-analyses (NMA) and ranked the prognostic effects of various FLT3i based on phase 2 and 3 RCT to check the most effective FLT3i.

## Methods

This study was conducted according to Preferred Reporting Items for Systematic Reviews and Meta-Analyses (PRISMA) (**Supplementary Table 1**) (11) and was registered at PROSPERO (CRD42020158077).

### Search Strategy and Study Selection

A literature search was conducted through databases of PubMed, Embase, Cochrane library, China National Knowledge Infrastructure, and Wanfang since inception until September 30th, 2020, following keywords “FMS-like tyrosine kinase 3”, “*FLT3*”, “acute myeloid leukemia”, “AML”, “hematopoietic stem cell transplant”, “HSCT”, “sorafenib”, “lestaurtinib”, “midostaurin”, “quizartinib”, and “gilteritinib”. The included reports were: (i) published in English or Chinese, (ii) limited to retrospective cohort studies or RCT reporting the therapeutic effects of FLT3i on AML, especially for FLT3(+) patients, or restricted to studies demonstrating prognostic effects of allo-HSCT on FLT3(+) AML, (iii) designed to contain two arms or more for comparing prognostic influence of FLT3i or allo-HSCT with controls. Studies were excluded if they: (i) reported unavailable or insufficient data, (ii) were reviews, case reports, editorials and letters, (iii) had overlapping cohorts and (iv) were sing-arm studies.

Study selection was conducted in two stages. Initially, abstracts and titles of potential literature were independently browsed and screened by QX and SH according to inclusion and exclusion criteria. Both reviewers then evaluated the candidate articles and decided on their inclusion. Any discrepancy was discussed and, if required, settled through discussion or consultation with a third reviewer (LY). After selecting the candidate studies, full texts were checked to identify final eligible ones.

### Quality Assessment and Publication Bias Investigation

The methodologic quality of primary studies was assessed separately by two reviewers (QX and SH), based on Newcastle-Ottawa-Scale (NOS) (12) and Cochrane Risk of Bias Tool (13) used for quality assessment of retrospective cohort studies and prospective RCT, respectively. Any disparity can be resolved through discussion panel. Publication bias was investigated with funnel plots as well as Begg’s (14) and Egger’s (15) tests. A *P*-value < 0.05 implied publication bias existence.

### Data Collection

Clinical information from the included studies was extracted independently by two authors (QX and SH), and any reported disagreement was settled by discussion or consultation with the third author (LY). The extracted data were comprised of the first author, study characteristics, patients’ baseline and prognostic information. The endpoints included overall survival (OS), event-free survival (EFS), relapse-free survival (RFS), cumulative incidence of relapse (CIR), early death (defined as induction death or 30-day mortality), adverse events and complete remission (CR), defined by revised International Working Group Criteria (16), without requirement of peripheral count recovery for CR. Hazard ratio (HR) was utilized to assess survival, and relative risk (RR) was utilized to evaluate CR, early death and adverse events. Data were preferentially extracted from multivariate analyses. However, in researches without multivariate data, RR and HR were exacted from univariate analyses or calculated from Kaplan-Meier survival curves or numeric reports under the methods provided by Tierney et al. (17).

### Statistical Analysis

The pooled HR and 95% confidence intervals (95% CI) for survival were calculated with the inverse variance method, and pooled RR and 95% CI for CR, early death and adverse events were produced from the Mantel-Haenszel method (18). Analyses were conducted with Stata 15.1 using random-effect models to account for heterogeneity between studies. Pooled RR or HR < 1.00 indicated better effects supporting FLT3i or allo-HSCT. It was also considered statistically significant with 95% CI range that did not cover 1.00 and a *p-*value of < 0.05. The χ2-based Q statistic was used to assess the heterogeneity among studies. Low, moderate, substantial and considerable heterogeneity showed *I^2^* < 30%, 30%–50%, 50%–75% and > 75%, respectively. A *P*-value ≥ 0.10 implied no heterogeneity or slight heterogeneity, whereas *P* < 0.10 meant significant heterogeneity existence (19). When *P*-value of heterogeneity was < 0.10, sensitivity and subgroup analyses were conducted to determine significant heterogeneity source.

Bayesian NMA was done with R 4.0.2 by means of a random model *via* packages of “gemtc” and “rjags” in RCT. We also calculated HR or risk ratios (RR) regarding non-FLT3i group as the baseline to act as the effect measure, displayed in forest plots, where RR and HR with 95% credible intervals (95% Crl) were utilized to explain the extent of effects in CR and survival, respectively. The range of 95% Crl without covering 1.00 implied statistical significance. To estimate relative HR and RR, a Markov Chain Monte Carlo simulation was finished with 10000 adaptations and 100000 iterations of each of the three automatically generated Markov chains. After finishing all simulations, NMA determined the probability that each treatment would be best by calculating the probability of simulations in which a certain treatment ranked best. For each iteration, therapies were ranked according to the evaluated log HR or log RR. The data from Bayesian NMA were compared with data from pairwise meta-analyses to assess inconsistency using the node splitting method (20). Significant inconsistency was indicated if node-splitting analysis showed a *P-*value < 0.05. If no closed-loop was present in the network evidence plot, inconsistency analysis could not be executed. Besides, the network evidence plots were drawn from Stata 15.1.

All analyses were based on published data; therefore, no ethical approval and patient consent were required.

## Results

### Characteristics of Included Studies

**Figure 1:**
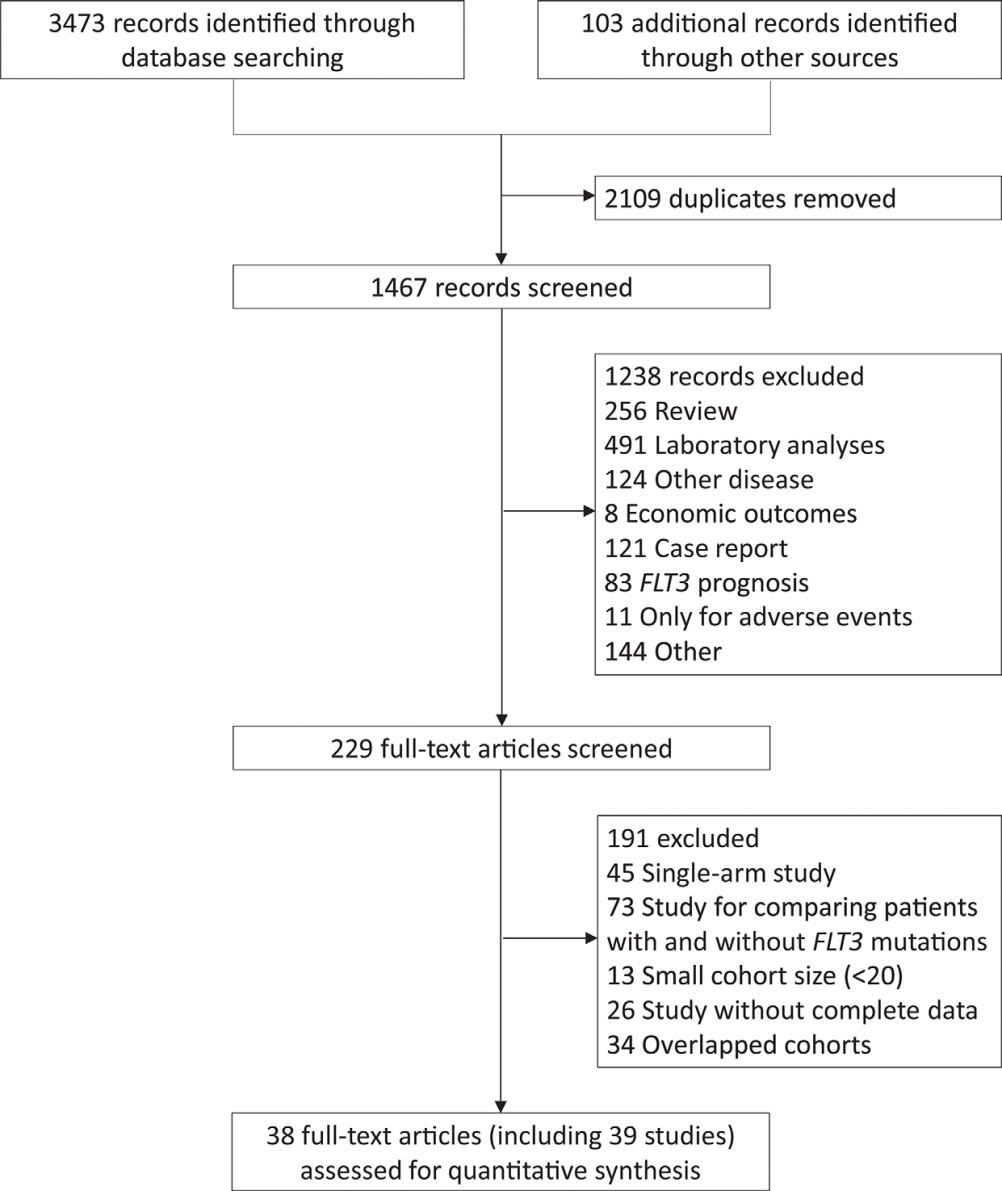
Flow diagram of the study selection. FLT3, FMS-like tyrosine kinase 3.

The study selection was shown in **Figure 1**. A total of 38 articles, including 39 studies, were eventually included. The 4^th^ study covered the 5^th^ cohort but focused on different disease statuses, while the 35^th^ and 36^th^ studies contained the same population but involved different treatments. The study characteristics and qualification assessment were listed in **Supplementary Table 2**.

Totally, 6859 AML patients were included, and their studies were conducted in *FLT3*(+) AML, except for Roellig et al. (7) and Serve et al. (21) (**Supplementary Table 2**), covering 464 newly diagnosed AML receiving sorafenib plus chemotherapy as induction and consolidation regimen, regardless of FLT3 mutational status. Three studies comprised 380 newly diagnosed *FLT3*(+) AML patients given sorafenib-related therapies as induction strategy, with or without consolidation and maintenance therapy following allo-HSCT. Six studies (*n* = 951) only focused on the maintenance therapy of sorafenib after allo-HSCT, and two studies (*n* = 235) regarded sorafenib as part of salvage therapy following relapse. Midostaurin was used in the stages of induction, consolidation and maintenance treatment in CR in four studies (*n* = 1604) and one article (*n* = 60) reported results of midostaurin-related maintenance therapy after allo-HSCT. For lestaurtinib studies, two (*n* = 500) focused on the induction stage in untreated *FLT3*(+) AML and one (*n* = 224) involved salvage therapy in *FLT3*(+) rrAML. In addition, the clinical efficacy of gilteritinib (*n* = 371) and quizartinib (*n* = 367) was evaluated in RCT for rrAML, and two studies (*n* = 178) combined several FLT3i. Finally, fourteen studies (*n* = 1797) compared prognostic discordance between allo-HSCT and non-HSCT in *FLT3*(+) AML.

Twenty-eight retrospective cohort studies and eleven RCT were included. The reported eleven RCT comprised four for sorafenib, two for midostaurin, one for gilteritinib, one for quizartinib, and three for lestaurtinib. **Supplementary Figures 1, 2** showed risk of bias in RCT quality assessment. For survival endpoints, we thought that bias was unlikely because relapse and death were the endpoints without susceptibility to patient, physician, or outcome assessor bias. The details for NOS score of retrospective studies were listed in **Supplementary Table 3**.

### Survival Benefits of FLT3i on Newly Diagnosed AML During Induction Treatment

**Figure 2:**
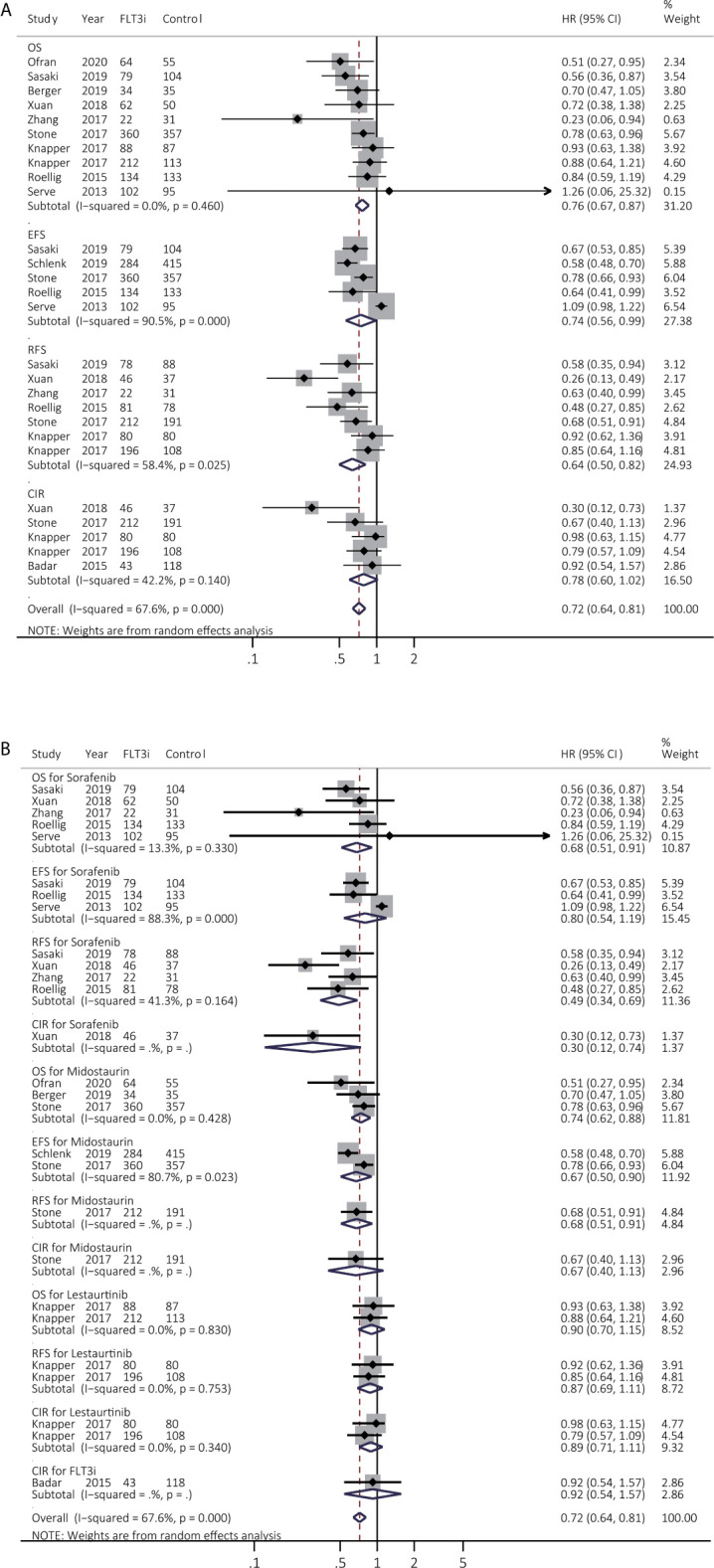
Survival influences of FLT3i on newly diagnosed AML during induction treatment. **(A)** Pooled HR and 95% CI of all FLT3i during induction treatment in newly diagnosed AML for OS, EFS, RFS and CIR; **(B)** Pooled HR and 95% CI of each FLT3i during induction treatment in newly diagnosed AML for OS, EFS, RFS and CIR. FLT3, FMS-like tyrosine kinase 3; FLT3i, FLT3 inhibitor; AML, acute myeloid leukemia; OS, overall survival; EFS, event-free survival; RFS, relapse-free survival; CIR, cumulative incidence of relapse; HR, hazard ratio; 95% CI, 95% confidence interval.

Twelve studies reported survival across various FLT3i in the induction stage. The summary HR consistently showcased prolonged OS (HR 0.76, 95% CI 0.67–0.87, *p* < 0.01; heterogeneity: *I^2^* = 0.0%, *P* = 0.460) in FLT3i groups but inconsistently in prolonged EFS (HR 0.74, 95% CI 0.56–0.99, *p* = 0.04; heterogeneity: *I^2^* = 90.5%, *P* = 0.000) and better RFS (HR 0.64, 95% CI 0.50–0.82, *p* < 0.01; heterogeneity: *I^2^* = 58.4%, *P* = 0.025) (**Figure 2A** and **Supplementary Table 4**). After excluding the studies of Serve et al. (EFS) (21) and Xuan et al. (RFS) (22), the combined HR became consistent in the sensitivity analyses, indicating longer EFS (HR 0.67, 95% CI 0.58–0.78, *p* < 0.01; heterogeneity: *I^2^* = 43.1%, *P* = 0.153) and better RFS (HR 0.72, 95% CI 0.60–0.85, *p* < 0.01; heterogeneity: *I^2^* = 14.3%, *P* = 0.323) (**Supplementary Figure 3** and **Supplementary Table 4**).

The subgroup analyses were further evaluated among various FLT3i (**Figure 2B** and **Supplementary Table 5**). Sorafenib consistently achieved better OS (HR 0.68, 95% CI 0.51–0.91, *p* = 0.01; heterogeneity: *I^2^* = 13.3%, *P* = 0.330) and RFS (HR 0.49, 95% CI 0.34–0.69, *p* < 0.01; heterogeneity: *I^2^* = 41.3%, *P* = 0.164). Besides, midostaurin consistently prolonged OS (HR 0.74, 95% CI 0.62–0.88, *p* < 0.01; heterogeneity: *I^2^* = 0.0%, *P* = 0.428) but lestaurtinib did not provide better survival (*p* > 0.05).

### Survival Benefits of Allo-HSCT and FLT3i After Allo-HSCT on *FLT3*(+) AML

**Figure 3:**
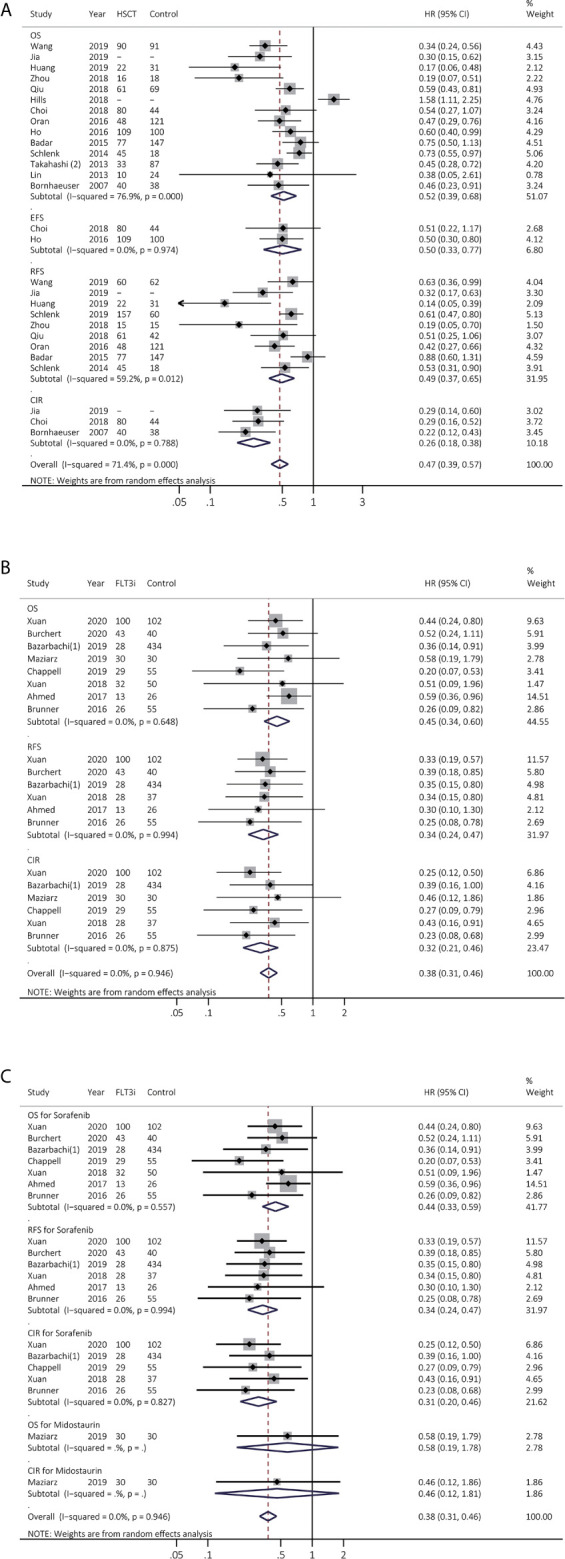
Survival influences of allo-HSCT and FLT3i as the maintenance therapy after allo-HSCT on FLT3(+) AML. **(A)** Pooled HR and 95% CI of allo-HSCT in FLT3 (+) AML for OS, EFS, RFS and CIR; **(B)** Pooled HR and 95% CI of all FLT3i as the maintenance therapy after allo-HSCT in FLT3 (+) AML for OS, RFS and CIR; **(C)** Pooled HR and 95% CI of each FLT3i as the maintenance therapy after allo-HSCT in FLT3 (+) AML for OS, RFS and CIR. FLT3, FMS-like tyrosine kinase 3; FLT3i, FLT3 inhibitor; Allo-HSCT, allogeneic hematopoietic stem cell transplant; AML, acute myeloid leukemia; OS, overall survival; EFS, event-free survival; RFS, relapse-free survival; CIR, cumulative incidence of relapse; HR, hazard ratio; 95% CI, 95% confidence interval.

The survival effects of allo-HSCT in *FLT3*(+) AML were then investigated in 14 studies (**Figure 3A** and **Supplementary Table 4**). EFS and CIR were remarkably consistently prolonged by allo-HSCT (EFS, HR 0.50, 95% CI 0.33–0.77, *p* < 0.01; heterogeneity: *I^2^* = 0.0%, *P* = 0.974; CIR, HR 0.26, 95% CI 0.18–0.38, *p* < 0.01; heterogeneity: *I^2^* = 0.0%, *P* = 0.788). OS and RFS were improved by allo-HSCT but with high heterogeneity (OS, HR 0.52, 95% CI 0.39–0.68, *p* < 0.01; heterogeneity: *I^2^* = 76.9%, *P* = 0.000; RFS, HR 0.49, 95% CI 0.37–0.65, *p* < 0.01; heterogeneity: *I^2^* = 59.2%, *P* = 0.012). After sensitivity analyses, combined HR of OS and RFS became consistent, demonstrating pooled HR of 0.53 for OS (95% CI 0.45–0.64, *p* < 0.01; heterogeneity: *I^2^* = 35.5%, *P* = 0.115) and pooled HR of 0.57 for RFS (95% CI 0.45–0.71, *p* < 0.01; heterogeneity: *I^2^* = 38.5%, *P* = 0.135), respectively (**Supplementary Figure 4**).

We next analyzed FLT3i role as the maintenance treatment after allo-HSCT in eight studies, consistently showing better survival (OS, HR 0.45, 95% CI 0.34–0.60, *p* < 0.01; heterogeneity: *I^2^* = 0.0%, *P* = 0.648; RFS, HR 0.34, 95% CI 0.24–0.47, *p* < 0.01; heterogeneity: *I^2^* = 0.0%, *P* = 0.994; CIR, HR 0.32, 95% CI 0.21–0.46, *p* < 0.01; heterogeneity: *I^2^* = 0.0%, *P* = 0.875; **Figure 3B** and **Supplementary Table 4**). In subgroup analyses (**Figure 3C** and **Supplementary Table 5**), sorafenib consistently exhibited longer survival (OS, HR 0.44, 95% CI 0.33–0.59, *p* < 0.01; heterogeneity: *I^2^* = 0.0%, *P* = 0.557; RFS, HR 0.34, 95% CI 0.24–0.47, *p* < 0.01; heterogeneity: *I^2^* = 0.0%, *P* = 0.994; CIR, HR 0.31, 95% CI 0.20–0.46, *p* < 0.01; heterogeneity: *I^2^* = 0.0%, *P* = 0.827).

### Survival Benefits of FLT3i on *FLT3*(+) rrAML During Salvage Therapy

**Figure 4:**
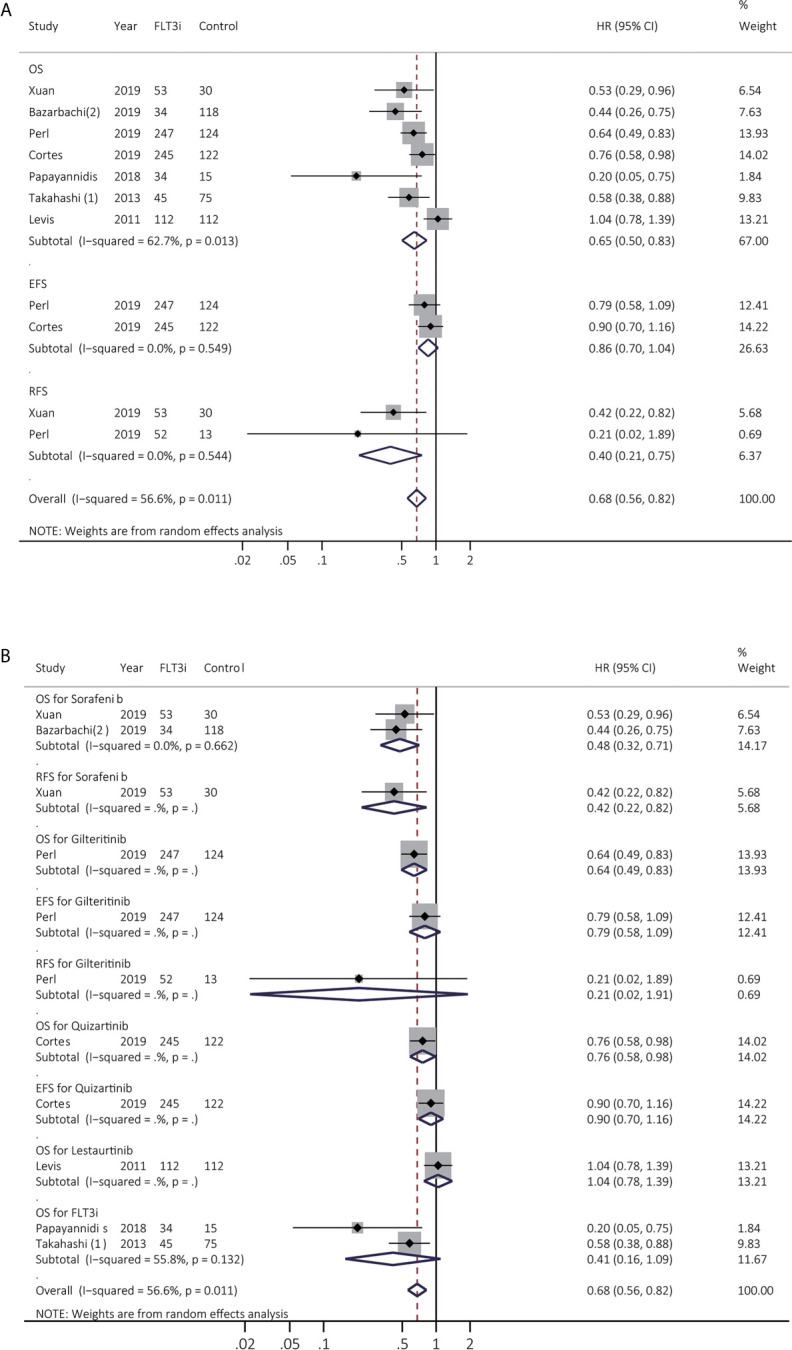
Survival influences of FLT3i on FLT3(+) rrAML. **(A)** Pooled HR and 95% CI of all FLT3i during salvage therapy in FLT3(+) rrAML for OS, EFS, and RFS; **(B)** Pooled HR and 95% CI of each FLT3i during salvage therapy in FLT3(+) rrAML for OS, EFS, and RFS. FLT3, FMS-like tyrosine kinase 3; FLT3i, FLT3 inhibitor; AML, acute myeloid leukemia; rrAML, refractory and relapsed AML; OS, overall survival; EFS, event-free survival; RFS, relapse-free survival; HR, hazard ratio; 95% CI, 95% confidence interval.

Prognostic effects of FLT3i in salvage regimen in rrAML were estimated among seven studies. After excluding Levis et al. (23) to decrease heterogeneity, the combined HR of OS was changed from 0.65 (95% CI 0.50–0.83, *p* < 0.01; heterogeneity: *I^2^* = 62.7%, *P* = 0.013; **Figure 4A**) to 0.60 (95% CI 0.49–0.74, *p* < 0.01; heterogeneity: *I^2^* = 29.6%, *P* = 0.213; **Supplementary Figure 5**) (**Supplementary Table 4**). RFS was consistently enhanced in FLT3i (HR 0.40, 95% CI 0.21–0.75, *p* = 0.01; heterogeneity: *I^2^* = 0.0%, *P* = 0.544; **Figure 4A** and **Supplementary Table 4**). In subgroup analyses (**Figure 4B** and **Supplementary Table 5**), pooled HR of OS in sorafenib was 0.48 (95% CI 0.32–0.71, *p* < 0.01; heterogeneity: *I^2^* = 0.0%, *P* = 0.662), and only one study was involved in gilteritinib, quizartinib, and lestaurtinib, respectively. Better OS was observed in gilteritinib (HR 0.64, 95% CI 0.49–0.83, *p* < 0.01) and quizartinib (HR 0.76, 95% CI 0.58–0.98, *p* = 0.04). However, lestaurtinib did not support better survival (*p* > 0.05).

### Response of FLT3i in AML

**Figure 5:**
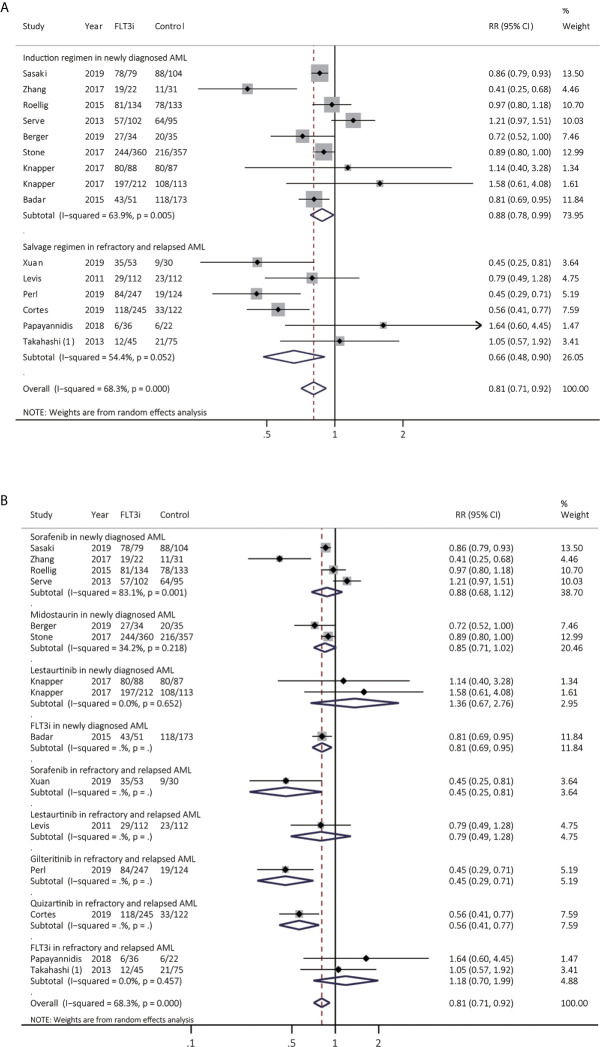
Response to FLT3i during induction/reinduction treatment of newly diagnosed AML and FLT3(+) rrAML. **(A)** Pooled RR and 95% CI of all FLT3i during induction treatment and salvage regimen for CR; **(B)** Pooled RR and 95% CI of each FLT3i during induction treatment and salvage regimen for CR. FLT3, FMS-like tyrosine kinase 3; FLT3i, FLT3 inhibitor; AML, acute myeloid leukemia; rrAML, refractory and relapsed AML; CR, complete remission; RR, relative risk; 95% CI, 95% confidence interval.

CR from 15 studies was assessed (**Figure 5A** and **Supplementary Table 4**), and better CR was heterogeneously realized in FLT3i group in both newly diagnosed AML (RR 0.88, 95% CI 0.78–0.99, *p* = 0.04; heterogeneity: *I^2^* = 63.9%, *P* = 0.005) and rrAML (RR 0.66, 95% CI 0.48–0.90, *p* = 0.01; heterogeneity: *I^2^* = 54.4%, *P* = 0.052). After sensitive analysis, combined RR of CR in rrAML became consistent, demonstrating pooled RR of 0.61 (95% CI 0.46–0.81, *p* < 0.01; heterogeneity: *I^2^* = 44.3%, *P* = 0.126). In the subgroup analyses for newly diagnosed AML (**Figure 5B** and **Supplementary Table 5**), pooled RR of sorafenib, midostaurin, and lestaurtinib was 0.88 (95% CI 0.68–1.12, *p* = 0.32; heterogeneity: *I^2^* = 83.1%, *P* = 0.001), 0.85 (95% CI 0.71–1.02, *p* = 0.08; heterogeneity: *I^2^* = 34.2%, *P* = 0.218) and 1.36 (95% CI 0.67–2.76, *p* = 0.40; heterogeneity: *I^2^* = 0.0%, *P* = 0.652), respectively. In the subgroup analyses for salvage regimen in rrAML (**Figure 5B** and **Supplementary Table 5**), HR for sorafenib, lestaurtinib, gilteritinib, and quizartinib was 0.45 (95% CI 0.25-0.81, *p* = 0.01), 0.79 (95% CI 0.49-1.28, *p* = 0.34), 0.45 (95% CI 0.29-0.71, *p* < 0.01), and 0.56 (95% CI 0.41-0.77, *p* < 0.01), respectively.

### Effectiveness of FLT3i to the *FLT3-ITD* Allelic Ratio

**Figure 6:**
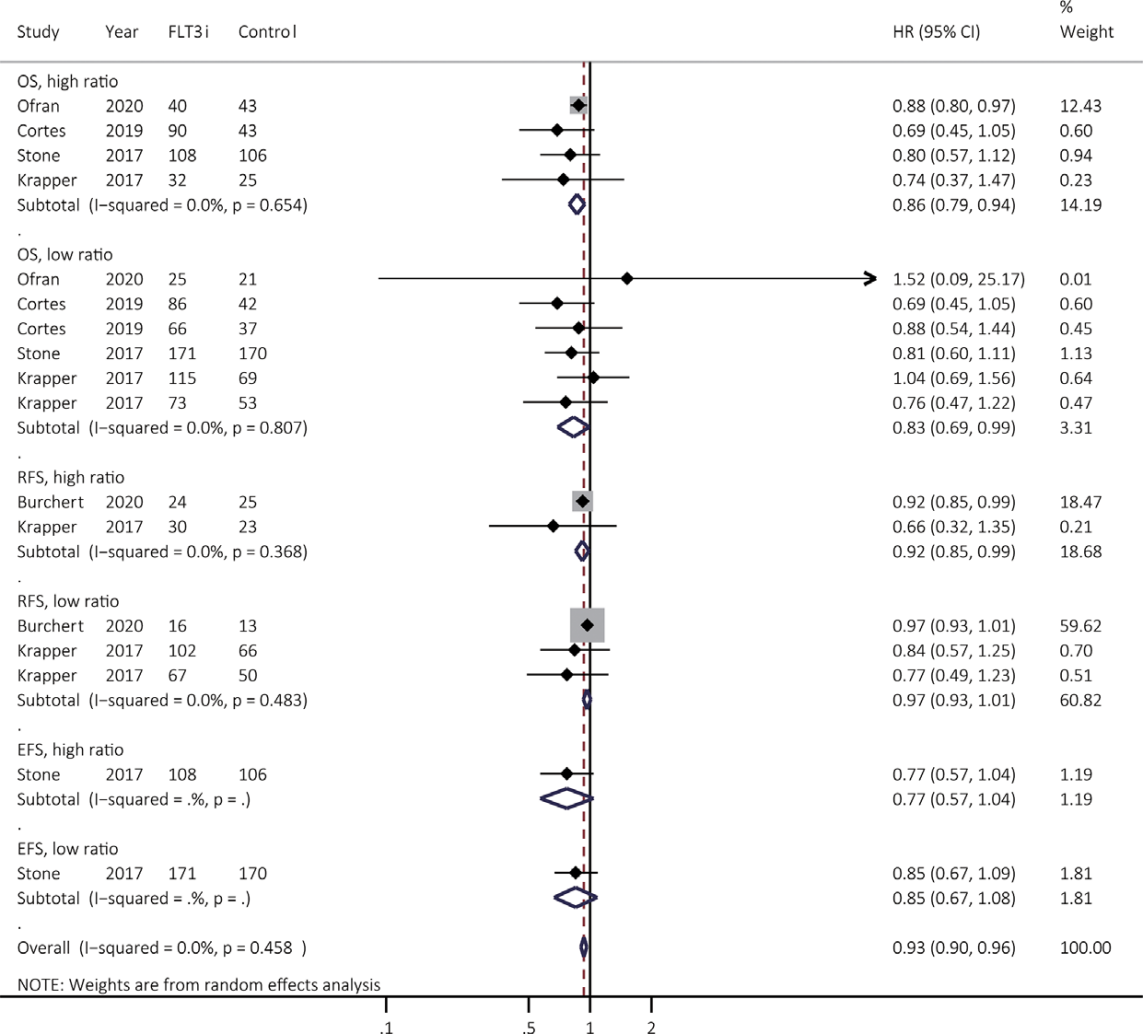
Survival effectiveness of FLT3i to the *FLT3-ITD* allelic ratio. Except for the study from Stone et al. (3), high ratio was identified as ≥ 0.5 as shown in the **Supplementary Table 6**. In the study from Stone et al. (3), high ratio was regarded as >0.7. FLT3, FMS-like tyrosine kinase 3; FLT3i, FLT3 inhibitor; *FLT3-ITD*, FMS-like tyrosine kinase 3-internal tandem duplication; OS, overall survival; EFS, event-free survival; RFS, relapse-free survival; HR, hazard ratio; 95% CI, 95% confidence interval.

The treatment effectiveness of FLT3i was then analyzed in patients with low and high *FLT3-ITD* allelic ratio, respectively. Due to the lack of relevant information involved in response, survival benefits of FLT3i were summarized in **Figure 6**. Exact stratifications of *FLT3-ITD* ratio in different studies were summarized in **Supplementary Table 6**. For OS, FLT3i consistently illustrated improved survival in both of high (HR 0.86, 95% CI 0.79–0.94, *p* < 0.01; heterogeneity: *I^2^* = 0.0%, *P* = 0.654) and low ratio (HR 0.83, 95% CI 0.69–0.99, *p* = 0.04; heterogeneity: *I^2^* = 0.0%, *P* = 0.807) when compared to non-FLT3i group. However, FLT3i significantly prolonged RFS in patients with high-ratio *FLT3-ITD* (HR 0.92, 95% CI 0.85–0.99, *p* = 0.03; heterogeneity: *I^2^* = 0.0%, *P* = 0.368) rather than those with low-ratio *FLT3-ITD* mutations (HR 0.97, 95% CI 0.93–0.1.01, *p* = 0.15; heterogeneity: *I^2^* = 0.0%, *P* = 0.483), when compared to non-FLT3i arm. Finally, there was only one study involved in EFS, showing no prolonged survival in FLT3i group in both of high and low *FLT3-ITD* ratio.

### Harm

Totally, early death and 21 types of toxic effects were analyzed (**Supplementary Table 7**) in sorafenib, gilteritinib, quizartinib, midostaurin, and lestaurtinib. FLT3i consistently showed significantly increased risk of thrombocytopenia (FLT3i *vs.* non-FLT3i: 28.96% *vs.* 18.36%; RR, 2.20; 95% CI, 1.39–3.48, *p* = 0.0008; *I^2^* = 55.2%, *P* = 0.108), neutropenia (FLT3i *vs.* non-FLT3i: 34.92% *vs.* 22.30%; RR, 1.32; 95% CI, 1.05–1.65, *p* = 0.016; *I^2^* = 0.0%, *P* = 0.620), as well as anemia (FLT3i *vs.* non-FLT3i: 41.89% *vs.* 33.50%; RR, 1.26; 95% CI, 1.01–1.57, *p* = 0.04; *I^2^* = 0.0%, *P* = 0.460) regardless of grades.

Besides, FLT3i were clearly related to higher risk of skin-related adverse effects at all grades (FLT3i *vs.* non-FLT3i: 27.42% *vs.* 22.62%; RR, 1.37; 95% CI, 1.01–1.87, *p* = 0.045; *I^2^* = 49.9%, *P* = 0.092), especially for grades ≥ 3 (FLT3i *vs.* non-FLT3i: 7.46% *vs.* 4.43%; RR, 2.07; 95% CI, 1.44–2.99, *p* = 0.0001) with low heterogeneity (*I^2^* = 0.0%, *P* = 0.543). FLT3i also consistently showed a tendency of increased risk for all-grade gastrointestinal-related toxicities (FLT3i *vs.* non-FLT3i: 38.80% *vs.* 26.28%; RR, 1.49; 95% CI, 1.00–2.23, *p* = 0.051; *I^2^* = 15.10%, *P* = 0.278). Additionally, significantly increased alanine aminotransferase was found in FLT3i group at all grades (FLT3i *vs.* non-FLT3i: 27.72% *vs.* 6.90%; RR, 4.13; 95% CI, 2.45–6.95, *p* = 0.000; *I^2^* = 0.0%, *P* = 0.528) and grade ≥ 3 (FLT3i *vs.* non-FLT3i: 10.45% *vs.* 7.90%; RR, 1.68; 95% CI, 1.04–2.72, *p* = 0.034; *I^2^* = 14.80%, *P* = 0.309). Cardiac-related adverse events were mainly reported in sorafenib in all grades (sorafenib *vs.* control: 23.08% *vs.* 15.74%; RR, 1.47; 95% CI, 1.01–2.14, *p* = 0.044; *I^2^* = 0.0%, *P* = 0.336) and grade ≥ 3 (sorafenib *vs.* control: 4.76% *vs.* 2.12%; RR, 2.68; 95% CI, 1.09–6.59, *p* = 0.032; *I^2^* = 0.0%, *P* = 0.407). Higher risk of all-grade cough (FLT3i *vs.* non-FLT3i: 26.28% *vs.* 11.82%; RR, 2.18; 95% CI, 1.28–3.73, *p* = 0.004; *I^2^* = 42.4%, *P* = 0.188) and dyspnea (FLT3i *vs.* non-FLT3i: 21.97% *vs.* 7.39%; RR, 2.92; 95% CI, 1.75–4.90, *p* = 0.000; *I^2^* = 0.0%, *P* = 0.415) was reported in FLT3i group.

Finally, there was no clear relationship between FLT3i as well as high risk of early death and acute/chronic graft-versus-host disease (GVHD).

### Sensitivity Analyses and Publication Bias

Sensitivity analyses were conducted if high heterogeneity (*P* < 0.10) existed. The heterogeneity sources were listed in **Supplementary Table 4**, which were related to age, various FLT3i, allo-HSCT, cytogenetics, and genetics. **Supplementary Table 8** displayed the publication bias in RFS of FLT3i in induction stage, OS and RFS of allo-HSCT in *FLT3*(+) AML, as well as OS of FLT3i in salvage regimen. The funnel plots were illustrated in **Supplementary Figures 6–10**.

### Network Meta-Analyses

**Supplementary Figures 11A–D** showed the network evidence plots for comparing CR, OS, EFS, and RFS between various FLT3i in AML, noting the lack of head-to-head trial except for analyses of OS and RFS. Consequently, for EFS and CR, the summarized data between interventions were produced either from qualified indirect or direct evidence but not from both, without ability to estimate the inconsistency between direct and indirect comparisons.

Regarding all endpoints included in NMA, gilteritinib accomplished a tendency of the best prognosis compared with standard chemotherapy, particularly in CR (RR 0.45, 95% Crl 0.20-1.10) and OS (HR 0.64, 95% Crl 0.39-1.00) (**Supplementary Figures 11E–H**), and also favored the highest probability of improving prognosis (**Supplementary Figures 11I–L**) when compared to other strategies. The detailed results of prognostic effects from all treatments were presented in **Supplementary Tables 9–12**. The results of node splitting method exhibited no inconsistency between indirect and direct evidence for OS and RFS (**Supplementary Figure 12**). No heterogeneity was found in all NMA, except for pooled RR of CR and HR of EFS from studies of Roellig et al. (7) and Serve et al. (21) (**Supplementary Figure 13**). The heterogeneity reason is that ages of AML patients from Roellig et al. (7) were aged 60 years or younger, but it was opposite in Serve et al. (21).

## Discussion

Until now, no consensus was present on FLT3i role in various AML treatment stages, especially for *FLT3*(+) AML, and which FLT3i might be the best, which were investigated in this study. FLT3i possibly supported better CR in induction and salvage therapy that further boosted the improved survival of FLT3i. Allo-HSCT improved survival in *FLT3*(+) AML and FLT3i as the maintenance therapy after allo-HSCT might further enhance survival benefit gained from allo-HSCT. FLT3i also improved OS regardless of stratification of *FLT3-ITD* ratio, when compared to non-FLT3i group. Additionally, FLT3i consistently showed significantly increased risk of thrombocytopenia, neutropenia, anemia, skin-related adverse effects, increased alanine aminotransferase, cardiac-related adverse events, cough and dyspnea, but were not associated with high early death and increased risk of GVHD. NMA showed that gilteritinib probably favored the highest possibility toward better prognosis, which should be identified in more direct head-to-head RCT.

The summarized data in induction stage favored the prognostic benefit from FLT3i in AML, especially for sorafenib and midostaurin, consistent with a preceding RCT. Roellig et al. (7), one of the largest two RCT of sorafenib, reported improved EFS and RFS of sorafenib in patients aged ≤ 60 years. A possible mechanism underlying these data even in wild-type *FLT3* was the antileukemic activity of sorafenib in inhibiting other kinases such as *RAF* (24), *KIT*, platelet-derived growth factor receptors, and vascular endothelial growth factor receptors (7). In contrast, Serve et al. (21), another largest RCT for sorafenib, exhibited a higher early mortality and increased toxicity without improved antileukemic efficacy of sorafenib in patients ≥ 60 years. The plausible explanation was the lower tolerability of elderly patients for sorafenib, as well as overexpressed multidrug-resistant phenotypes and, probably, more epigenetic changes in elderly cohorts (25), which offset the targeting effect of sorafenib for *FLT3* and contributed to the heterogeneity of summarized EFS in induction stage in our study. For midostaurin, a large RCT (RATIFY) (3) reported that among *FLT3*(+) patients aged 18-59 years, midostaurin plus chemotherapy achieved prolonged survival, regardless of *FLT3-TKD* and different levels of mutant *FLT3-ITD* ratio, probably resulting from enough exposure to this inhibitor. Given a benefit among patients with low allelic *FLT3-ITD* mutation and a large disease burden, the benefit of this multitargeted kinase inhibitor might lie beyond its ability to inhibit *FLT3*, like inhibiting *KIT* in *FLT3* (–) AML (3). Finally, Knapper et al. (10) confirmed that lestaurtinib + chemotherapy had no enhanced prognosis among younger *FLT3*(+) AML, possibly due to the rising level of *FLT3* ligand induced by chemotherapy, which could interfere with activity of *FLT3* inhibition, including lestaurtinib (10).

Next, allo-HSCT possibly also enhanced survival in *FLT3*(+) AML. However, some variables greatly affect the effectiveness of allo-HSCT, including disease status (CR or not), *FLT3* variables (allelic burden and co-mutations), and using FLT3i before and/or after allo-HSCT. Unfortunately, no RCT assessed the most suitable post-remission treatment in *FLT3*(+) AML, considering diverse combinations (26). However, we herein summarized the maintenance effects of FLT3i following allo-HSCT, since even after allo-HSCT, early relapse frequently occurred in *FLT3*(+) AML (30%-59%) (27). Indeed, increased survival was observed in FLT3i as the maintenance therapy after allo-HSCT, especially for sorafenib. Similarly, one recent phase 3 RCT (28) showed enhanced survival and high tolerance of sorafenib, identifying safety and availability of sorafenib after allo-HSCT. Moreover, in a large retrospective study including 144 *FLT3-ITD*(+) patients undergoing allo‐HSCT (22), probability of CIR and OS was the best in sorafenib administrated both before and after allo-HSCT compared to either alone and non-sorafenib group. Based on these results, further clinical trials should be determined to directly compare availability between four arms above. Besides, sorafenib, midostaurin and gilteritinib were currently evaluated for maintaining after allo-HSCT in *FLT3-ITD*(+) AML. In the RADIUS phase 2 RCT (29), midostaurin achieved a slight tendency of better OS and CIR. Gilteritinib is prospectively estimated in a phase 3 RCT (NCT02997202). Overall, for the foreseeable future, *FLT3*(+) AML patients may still benefit from sorafenib after allo-HSCT because of the less use of other FLT3i (26).

Due to high refractory and relapse incidence in *FLT3*(+) AML (6) and limited knowledge to treat *FLT3*(+) rrAML, we also explored consistent or different therapeutic efficiency between various FLT3i in such patients. Except for lestaurtinib, the combined OS could be consistently improved by sorafenib, gilteritinib, and quizartinib, showing the potential of FLT3i in treating *FLT3*(+) rrAML. A phase 3 RCT randomized 371 *FLT3*(+) rrAML to either gilteritinib or salvage chemotherapy (6), showing increased survival as well as decreased frequency of adverse events in gilteritinib. Results from a similar phase 3 RCT (QuANTUM-R) comparing quizartinib with salvage chemotherapy in 367 *FLT3*(+) rrAML patients also confirmed quizartinib availability in improving prognosis (*p* < 0.05). Until now, the promising efficiency of gilteritinib and quizartinib could be observed in *FLT3*(+) rrAML in the two large RCT. However, a lack of evidence is present to add the two FLT3i to induction, consolidation, and maintenance therapy after allo-HSCT. Several phase 2 or 3 RCT are being processed (Gilteritinib: NCT02927262, NCT02997202, and NCT02752035; Quizartinib: NCT02668653). Additionally, two retrospective studies of sorafenib from Bazarbachi et al. (30) and Xuan et al. (31) also revealed enhanced OS comparing sorafenib with salvage chemotherapy in *FLT3*(+) rrAML, which should be further confirmed in RCT. Finally, midostaurin might be specifically effective in the untreated AML rather than rrAML, since *in vitro* studies, midostaurin had broader activity and might achieve greater clinical utility in newly diagnosed AML with blasts tending to be less addicted to *FLT3*-mediated signaling than rrAML (32).

We also explored the effectiveness of FLT3i on *FLT3-ITD*(+) AML stratified by ITD allelic ratios, showing that FLT3i consistently achieved significantly improved OS in both of high and low ratio crossing sorafenib, quizartinib, midostaurin and even lestaurtinib, further suggesting the benefits of FLT3i in *FLT3-ITD*(+) AML. However, there was no information involved in assessing clinical efficiency of gilteritinib in patients with different levels of *FLT3-ITD* ratio, which needs to be further explored in more studies.

For adverse events, FLT3i were significantly associated with increased risk of thrombocytopenia, neutropenia and anemia regardless of grades, high risk of skin- and cardiac-related adverse effects, especially for grades ≥ 3, all-grade increased alanine aminotransferase, high risk of all-grade cough and dyspnea, as well as a tendency of increased risk for all-grade gastrointestinal-related toxicities. However, these adverse events were generally manageable based on treatment interruptions or dose reductions (2). Moreover, there was no significant relationship between FLT3i as well as high early death and high risk of GVHD, demonstrating the safety of FLT3i.

Finally, due to co-existence of several FLT3i, it would also be meaningful to explore which inhibitor might be the best. Herein, we finished an NMA based on all RCT to settle this problem. The results displayed that gilteritinib probably tended to favor the highest probability of improving prognosis compared with other FLT3i, standard of care and standard chemotherapy. The relevant RCT involved in gilteritinib for rrAML treatment also illustrated improved survival as mentioned above (6). However, these results and corresponding consequences were relatively limited, given the rather small improvement obtained which each FLT3i through the indirect comparison. More direct head-to-head RCT with large cohort size are required to explore the different clinical efficiency between these FLT3i.

Overall, our study was up to now the biggest and the most comprehensive meta-analysis involved in various FLT3i in AML, containing all RCT and retrospective cohort studies. In addition to FLT3i function in induction stage in untreated AML, the role of allo-HSCT in *FLT3*(+) AML and therapeutic efficiency of FLT3i as maintenance therapy after allo-HSCT and as salvage regimens in rrAML were summarized and explored, showing that combining FLT3i, especially sorafenib, into the treatment before and after allo-HSCT might be more beneficial in improving prognosis, which should be further explored in RCT. Our study also primitively proposed NMA to compare various FLT3i, but with limitations as mentioned above. There were also several limitations in this study. Of 39 studies, 28 studies were retrospective, making it difficult to precisely control selection, attrition, information and confounding bias. In particular, due to RCT lack, limited results and conclusion were obtained from NMA. Besides, some data were extracted from Kaplan-Meier survival curves and numeric reports, probably resulting in slight disparity with the fact. Finally, this study did not make subgroup analyses based on age, co-existing mutations with *FLT3* mutations, and cytogenetic stratifications due to limited relevant data in primary studies. As a consequence, we will continue updating this study to explore suitable treatment of FLT3i in AML patients.

## Conclusions

In this work, FLT3i reinforced better prognosis in the induction stage of newly diagnosed *FLT3*(+) AML and salvage therapy of *FLT3*(+) rrAML and further enhanced survival advantages from allo-HSCT as the maintenance therapy. This probably indicates that better prognosis could be achieved if FLT3i is added into both treatments before and after allo-HSCT. Besides, FLT3i probably improved OS regardless of *FLT3-ITD* ratio. Additionally, FLT3i were significantly linked to increased risk of all-grade thrombocytopenia, neutropenia, anemia, all-grade skin- and cardiac-related adverse effects, especially for grade ≥ 3, as well as all-grade increased alanine aminotransferase, enhanced risk of all-grade cough and dyspnea, and a tendency of increased risk for all-grade gastrointestinal-related toxicities, but not related to higher incidence of early death and GVHD compared to non-FLT3i group. In NMA, gilteritinib potentially accomplished the best prognosis, which should be identified in direct head-to-head RCT.

## Data Availability Statement

The original contributions presented in the study are included in the article/**Supplementary Material**. Further inquiries can be directed to the corresponding author.

## Author Contributions

QX and LY contributed to conception and design of the study. QX and SH collected data from database. QX and SH performed the statistical analyses. QX and LY wrote the first draft of the manuscript. All authors contributed to the article and approved the submitted version.

## Funding

This work was supported by Chinese National Major Project for New Drug Innovation (2019ZX09201002003), National Natural Science Foundation of China (82030076, 82070161, 81970151, 81670162 and 81870134), Shenzhen Science and Technology Foundation (JCYJ20190808163601776, JCYJ20200109113810154) and Shenzhen Key Laboratory Foundation (ZDSYS20200811143757022).

## Conflict of Interest

The authors declare that the research was conducted in the absence of any commercial or financial relationships that could be construed as a potential conflict of interest.
