# Supplementary material for: Clinical Benefits and Safety of FMS-Like Tyrosine Kinase 3 Inhibitors in Various Treatment Stages of Acute Myeloid Leukemia: A Systematic Review, Meta-Analysis, and Network Meta-Analysis
Source: Front Oncol. 2021 Jun 3;11:686013. doi: 10.3389/fonc.2021.686013 (PMC8209493; doi:10.3389/fonc.2021.686013)
Supplement: Supplementary file 1 [file DataSheet_1.docx]

Supplementary Material

# Supplementary Tables

**Supplementary Table 1. The preferred reporting items for systematic reviews and meta-analyses (PRISMA).**

| Section/topic | # | Checklist item | Reported on page # |
| --- | --- | --- | --- |
| **TITLE** | | | |
| Title | 1 | Identify the report as a systematic review, meta-analysis, or both. | 1 |
| **ABSTRACT** | | | |
| Structured summary | 2 | Provide a structured summary including, as applicable: background; objectives; data sources; study eligibility criteria, participants, and interventions; study appraisal and synthesis methods; results; limitations; conclusions and implications of key findings; systematic review registration number. | 1,2 |
| **INTRODUCTION** | | | |
| Rationale | 3 | Describe the rationale for the review in the context of what is already known. | 2 |
| Objectives | 4 | Provide an explicit statement of questions being addressed with reference to participants, interventions, comparisons, outcomes, and study design (PICOS). | 2 |
| **METHODS** | | | |
| Protocol and registration | 5 | Indicate if a review protocol exists, if and where it can be accessed (e.g., Web address), and, if available, provide registration information including registration number. | PROSPERO (CRD42020158077) |
| Eligibility criteria | 6 | Specify study characteristics (e.g., PICOS, length of follow-up) and report characteristics (e.g., years considered, language, publication status) used as criteria for eligibility, giving rationale. | 3 |
| Information sources | 7 | Describe all information sources (e.g., databases with dates of coverage, contact with study authors to identify additional studies) in the search and date last searched. | 3 |
| Search | 8 | Present full electronic search strategy for at least one database, including any limits used, such that it could be repeated. | 3 |
| Study selection | 9 | State the process for selecting studies (i.e., screening, eligibility, included in systematic review, and, if applicable, included in the meta-analysis). | 3 |
| Data collection process | 10 | Describe method of data extraction from reports (e.g., piloted forms, independently, in duplicate) and any processes for obtaining and confirming data from investigators. | 3 |
| Data items | 11 | List and define all variables for which data were sought (e.g., PICOS, funding sources) and any assumptions and simplifications made. | 3 |
| Risk of bias in individual studies | 12 | Describe methods used for assessing risk of bias of individual studies (including specification of whether this was done at the study or outcome level), and how this information is to be used in any data synthesis. | 3 |
| Summary measures | 13 | State the principal summary measures (e.g., risk ratio, difference in means). | 3 |
| Synthesis of results | 14 | Describe the methods of handling data and combining results of studies, if done, including measures of consistency (e.g., I^2^) for each meta-analysis. | 3, 4 |
| Risk of bias across studies | 15 | Specify any assessment of risk of bias that may affect the cumulative evidence (e.g., publication bias, selective reporting within studies). | 3 |
| Additional analyses | 16 | Describe methods of additional analyses (e.g., sensitivity or subgroup analyses, meta-regression), if done, indicating which were pre-specified. | 4 |
| RESULTS | | | |
| Study selection | 17 | Give numbers of studies screened, assessed for eligibility, and included in the review, with reasons for exclusions at each stage, ideally with a flow diagram. | 4 |
| Study characteristics | 18 | For each study, present characteristics for which data were extracted (e.g., study size, PICOS, follow-up period) and provide the citations. | 4, 5 |
| Risk of bias within studies | 19 | Present data on risk of bias of each study and, if available, any outcome-level assessment (see Item 12). | 5 |
| Results of individual studies | 20 | For all outcomes considered (benefits or harms), present, for each study: (a) simple summary data for each intervention group and (b) effect estimates and confidence intervals, ideally with a forest plot. | 5, 6, 7 |
| Synthesis of results | 21 | Present results of each meta-analysis done, including confidence intervals and measures of consistency. | 5, 6, 7 |
| Risk of bias across studies | 22 | Present results of any assessment of risk of bias across studies (see Item 15). | 7 |
| Additional analysis | 23 | Give results of additional analyses, if done (e.g., sensitivity or subgroup analyses, meta-regression [see Item 16]). | 5, 6, 7 |
| DISCUSSION | | | |
| Summary of evidence | 24 | Summarize the main findings including the strength of evidence for each main outcome; consider their relevance to key groups (e.g., health care providers, users, and policy makers). | 7, 8, 9 |
| Limitations | 25 | Discuss limitations at study and outcome level (e.g., risk of bias), and at review level (e.g., incomplete retrieval of identified research, reporting bias). | 9 |
| Conclusions | 26 | Provide a general interpretation of the results in the context of other evidence, and implications for future research. | 1, 9, 10 |
| FUNDING | | | |
| Funding | 27 | Describe sources of funding for the systematic review and other support (e.g., supply of data); role of funders for the systematic review. | 10 |

**Supplementary Table 2.** **Study characteristics included into this systematic review, meta-analysis and network meta-analysis.**

| **First Author** | **Year** | **Region** | **Research duration** | **Research type** | **NCT No.** | **Assessment of quality** | **Median age (ranges) or Mean age (±SE)** | **Patient cohorts** | **Treatment stage** | **Research group** | **Combination regimen in research group** | **N of research group** | **Control regimen** | **N of control group** |
| --- | --- | --- | --- | --- | --- | --- | --- | --- | --- | --- | --- | --- | --- | --- |
| Xuan(1) | 2020 | China | 2015.6-2018.7 | A phase III randomized, open-label, multicentre trial | NCT02474290 | 6 | Sorafenib: 35 (26–42); Control: 35 (26–43) | FLT3-mutated AML receiving allo-HSCT | Maintenance therapy post allo-HSCT | Patients received sorafenib from day 30 until day 180 post-transplantation. For patients not meeting inclusion criteria on day 30 but did so within 31–60 days post-transplantation, sorafenib was used at 31–60 days post-transplantation and continued until day 180. The initial dose of sorafenib was 400 mg orally twice daily. If adverse events of ≥grade 3 occurred, dose modifications were allowed, including either dose reductions to 200 mg once or twice a day or dose interruptions. After resolution of adverse events, the sorafenib dose was re-escalated from 200 mg either once or twice a day to 400 mg twice a day. | - | 100 | - | 102 |
| Ofran(2) | 2020 | Israeli | 2015-2019 | A retrospective research | - | 8 | 56.3 ± 14.2; Midostaurin: 57.6 ± 14.8; Control: 54.9 ± 13.5 | Newly diagnosed FLT3-ITD mutated AML | Induction, consolidation and maintenance therapy after CR | The midostaurin regimen was identical to that applied in the RATIFY trial (50 mg twice a day for two weeks after each intensive chemotherapy cycle followed by 12 months of maintenance upon completion of chemotherapy). Maintenance therapy was also administered to some of our patients after recovery from allo-SCT. | Conventional standard regimen | 64 | Conventional standard regimen | 55 |
| Burchert(3) | 2020 | Germany and Austria | 2010.10-2016.5 | A phase II randomized, double-blind, placebo-controlled, multicenter trial | EudraCT 2010-018539-16 and DRKS00000591) | 6 | 54 (18.58-75.58); Sorafenib: 54.17 (23.58-74.58), Control: 54.59 (18.58-75.58) | FLT3-ITD mutated AML in CR1 receiving allo-HSCT | Maintenance therapy post allo-HSCT | The dose of sorafenib was escalated from 2 tablets (equivalent to 2 x 200 mg sorafenib) per day for 2 weeks (dose level 1), to 3 tablets per day for 4 weeks (dose level 2), up to the full dose of 2 x 2 tablets per day (dose level 3) thereafter. The full dose was equivalent to 800 mg. | - | 43 | Placebo was used as sorafenib. | 40 |
| Bazarbachi (1)(4) | 2019 | Lebanon, France, Italy, the Netherlands, Spain, Israel | 2010-2015 | A retrospective research | - | 6 | 50 (19-75) | FLT3-mutated AML receiving allo-HSCT | Maintenance therapy post allo-HSCT | Sorafenib treatment was initiated at a median of 55 days posttransplant (range 1–173) at a median dose of 800 (range 200–800) mg daily. | - | 28 | - | 434 |
| Bazarbachi (2)(5) | 2019 | Lebanon, France, Italy, the Netherlands, Spain, Israel | 2010-2015 | A retrospective research | - | 7 | Sorafenib: 48 (19-69); Control: 51 (19-75) | FLT3-ITD mutated, replased AML after allo-HSCT | Salvage therapy after relapse post allo-HSCT | Patients received sorafenib after allo-HSCT. Sorafenib was initiated after a median of 13 days (range 1-128) from relapse at the dose of 800 mg/day in 21 patients, 400 mg/day in 12, and 200 mg/day in one patient. | Conventional salvage chemotherapy | 34 | Used as the combination regimen in research group | 118 |
| Sasaki(6) | 2019 | USA | 2001.2-2017.12 | A retrospective research | - | 9 | 52 (17-65); Sorafenib: 53 (20-64), Control: 51 (17-65) | Newly diagnosed FLT3-ITD mutated AML | Induction and consolidation therapy | Sorafenib was administered during the first 7 or 14 days of induction, at doses of 400 mg orally twice daily; and at doses of 400mg orally twice daily for up to 28 days per cycle during consolidation. | Induction regimens included the IA combinations (CIA, CLIA, FIA, IA); Patients without allo-HSCT received consolidation with attenuated dose chemotherapy similar to their induction therapy. | 79 | Used as the combination regimen in research group | 104 |
| Schlenk(7) | 2019 | German-Austrian | Research cohort: 2012.6- 2016.5; Control cohort: 1993-2008 | A phase II, single-arm, open-label, multi-center trial; The historical controls comprise 5 previous AMLSG trials recruiting between 1993 and 2008 | NCT01477606, AMLSG 16-10 | 7 | AMLSG 16-10: 54.1 (18-70), Historical controls: 50.5 (18-70) | Newly diagnosed FLT3-ITD mutated AML | Induction, consolidation and maintenance therapy after allo-HSCT and HiDAC | Induction: Midostaurin was used orally at 50 mg twice daily from day8, until 48 hours before the start of the subsequent chemotherapy cycle. Consolidation: Midostaurin was used orally at 50 mg twice daily from day 6, until 48 hours before start of conditioning therapy for allo-HSCT or 48 hours before start of subsequent consolidation chemotherapy. Maintenance: Midostaurin was intended after allo-HSCT or after HiDAC, given orally 50mg twice daily for 365 days. After consolidation therapy with HiDAC, midostaurin was continued after the last applied cycle. After allo-HSCT, midostaurin was started at the earliest 30 days and at the latest 100 days after transplantation. | Induction regimen: patients received one induction cycle of 7+3. Patients achieving partial remission received an optional second cycle of induction therapy identical to the first cycle. Consolidation: Patients in CR or CRi received consolidation therapy. In case allo-HSCT was not possible, patients received up to 4 cycles of high-dose Ara-c (3 g/m^2^, patients > 65 years: 1 g/m^2^) twice daily on days 1, 3, and 5. | 284 | Treatment in all patients consisted of induction therapy with idarubicin, Ara-c, etoposide, and up to 4 cycles of high-dose Ara-c-based consolidation therapy. Allo-HSCT in first CR was performed on investigators discretion. | 415 |
| Berger(8) | 2019 | Israel | 2014-2018 | A retrospective research | - | 7 | 60 (26–82); Midostaurin: 60 (31-76), Control: 60 (26-82) | Newly diagnosed FLT3 mutated AML | Induction, consolidation and maintenance therapy after CR | Midostaurin was at 50 mg orally twice daily days 8-21 after the induction therapy. For consolidation, midostaurin was used at 50 mg orally twice daily days 8-14. Maintenance therapy was given to participants who continued in remission for 12 28-day cycles of midostaurin 50 mg orally twice daily. | Induction therapy included daunorubicin 60 mg/m^2^ by IV push day1-3 + Ara-c 200 mg/m^2^ IV day1-7, and was followed by consolidation therapy with four 28-day cycles of HiDAC (3g/m^2^) days 1, 3, & 5 for patients in CR after induction therapy. | 34 | Induction therapy included continuous 100 mg/m^2^ Ara-c for 7 days in combination with daunorubicin on days 1-3 (‘3 + 7’) for most patients and was followed by consolidation therapy with intermediate- or high-dose Ara-c or allo-HSCT. | 35 |
| Chappell(9) | 2019 | USA | 2008.1-2017.12 | A retrospective research | - | 5 | 55(10-71); Sorafineb: 49 (13-71), Control: 54 (10-71) | FLT3-ITD mutated AML after allo-HSCT | Maintenance therapy post allo-HSCT | Sorafenib was initiated a median of 78 days (range, 36–297) post allo-HSCT, and continued for a median of 381 days (range, 18–805). The median starting dose was 400 mg/day (range, 100–800/day). 26 subjects had toxicities, leading to dose changes in 19 and dose holds in 21 subjects lasting a median of 30 days. The median ending dose was 200 mg/day (100–800 mg/day). | - | 29 | Not receiving sorafenib after allo-HSCT. | 55 |
| Maziarz(10) | 2019 | USA and Canada | 2014.2-2018.4 | A phase II randomized, open-label exploratory trial | NCT01883362 | 4 | 18-70 | FLT3-ITD mutated AML after allo-HSCT | Maintenance therapy post allo-HSCT | Adults were randomized 28 to 60 days after allo-HSCT to receive standard of care ＋ midostaurin 50 mg twice daily for up to 12 (4-week) cycles: Midostaurin was supplied in 25mg taken orally twice a day for 28 days of each cycle, totally for 12 cycles. | - | 30 | Eligible adults with allo-HSCT in CR1 were randomized 28 to 60 days after allo-HSCT to receive standard of care for up to 12 (4-week) cycles. | 30 |
| Xuan(11) | 2019 | China | 2012.1-2017.10 | A retrospective research | - | 8 | 36 (14-59); Sorafenib: 37 (15-59); Control: 35 (14-57) | FLT3-ITD mutated, replased AML after allo-HSCT | Salvage therapy after relapse post allo-HSCT | Sorafenib was started at 400 mg twice daily and adjusted based on suspected toxicity (dose range, 200 to 800 mg daily). | Four salvage regimens were administered after relapse post-HSCT, including sorafenib plus chemotherapy followed by DLI or not. The chemotherapy included theaclacinomycin, Ara-c, and G-CSF regimen, the IA regimen, and others. For patients without grades II to >II aGVHD or extensive cGVHD at the time of relapse, DLI was administered at the following day of chemotherapy end if donor lymphocytes were available. | 53 | Four salvage regimens were administered after relapse post-transplant, including chemotherapy followed by DLI (n = 17), and monochemotherapy (n= 13). The salvage chemotherapy regimens were the same with the sorafenib group. | 30 |
| Perl(12) | 2019 | USA, Canada, France, Germany, Israel, Italy, Japan, Korea, Poland, Spain, Taiwan, Turkey, UK | 2015.10-2018.10 | A phase III randomized, open-label, multicenter trial | NCT02421939 | 6 | 62 (19-85); Gilteritinib: 62(20-84), Control: 61.5(19-85) | FLT3 mutated, refractory or relapsed AML | Salvage therapy for refractory or relapsed AML | Participants received 120 mg dose (3 tablets of 40 mg) of gilteritinib orally once a day in continuous 28-day cycles, at least 2 hours after or 1 hour before food. Gilteritinib treatment continued until participants met one of the treatment discontinuation criteria. | - | 247 | Patients received chemotherapy in 28-day cycles. Participants on LoDAC received 20 mg of Ara-c twice daily by SC or IV for 10 days. Participants on azacitidine received 75 mg/m^2^ daily by SC or IV for 7 days. Participants on LoDAC or azacitidine treatment continued until they met discontinuation criteria. Participants on MEC chemotherapy received mitoxantrone 8 mg/m^2^ daily by IV for 5 days, etoposide 100 mg/m^2^ daily by IV for 5 days and Ara-c 1000 mg/m^2^ daily by IV for 5 days (days 1-5). Participants on FLAG-IDA received G-CSF 300 μg/m^2^ daily by SC/IV on day1-5, fludarabine 30 mg/m^2^ daily by IV on day2-6, Ara-c 2000 mg/m^2^ daily by IV on day2-6 and idarubicin 10 mg/m^2^ daily by IV on day2-4). Participants receiving MEC or FLAG-IDA received 1 cycle of therapy and were assessed for response on or after day 15. | 124 |
| Cortes(13) | 2019 | USA, Australia, Belgium, Canada, Croatia, Czechia, France, Germany, Hongkong, Hungary, Italy, Korea, Netherlands, Poland, Serbia, Singapore, Spain, Taiwan, UK | 2014.5-2017.9 | A phase III randomized, open-label, multicenter trial | NCT02039726 | 6 | Quizartinib: 55 (46–65); Control: 57.5 (44–66) | FLT3-ITD mutated, refractory or relapsed AML | Salvage therapy for refractory or relapsed AML | Patients randomly received quizartinib dihydrochloride starting from 30 mg orally once daily, creased to 60 mg once daily on day 16 (±1 day) of cycle 1 if the patient’s mean QTcF interval of triplicate ECG readings was ≤450 ms on or before day 15 of cycle 1 (±1 day). Patients receiving concurrent strong CYP3A inhibitors had a reduced starting dose of 20 mg once daily, which was increased to 30 mg once daily if the same QT interval criteria were met. Quizartinib was given in continuous 28-day cycles with or without food. Stepwise dose reductions were permitted: 53·0 mg to 26·5 mg to 17·7 mg free base daily for patients with CYP3A inhibitor initiation, QT prolongation, non-haematological toxicity, or myelosuppression. For patients randomly assigned to the quizartinib group who underwent allo-HSCT, quizartinib was discontinued 7 days before the start of a conditioning regimen. Treatment with quizartinib after allo-HSCT was allowed per protocol starting at 30–100 days after the transplant. After allo-HSCT, patients started quizartinib at 26·5 mg or 17·7 mg free base daily and escalated to 53·0 mg or 26·5 mg free base daily using the same criteria as for initial treatment. | - | 245 | Up to 2 cycles of MEC or FLAG-IDA were permitted, patients assigned to salvage chemotherapy received either LoDAC (SC injection of Ara-c 20 mg twice daily on days 1–10 of 28-day cycles), MEC (IV infusions of mitoxantrone 8 mg/m² per day, etoposide 100mg/m² per day, and Ara-c 1000 mg/m² per day on days 1–5 of up to two 28-day cycles), or FLAG-IDA (G-SCF IV infusion 300 μg/m² per day or 5 μg/kg per day SC on days 1–5, fludarabine IV infusion 30 mg/m² per day on days 2–6, Ara-c IV infusion 2000 mg/m² per day on days 2–6, and IV infusion idarubicin 10 mg/m² per day on days 2–4 in up to two 28-day cycles). | 122 |
| Wang(14) | 2019 | China | 2006.4-2016.7 | A retrospective research | - | 6 | - | Newly diagnosed FLT3-ITD mutated AML | Allo-HSCT vs. non-HSCT | Allo-HSCT | Most patients received IA/DA. Older patients and patients received CAG (G-SCF, low-dose Ara-c and aclarubicin), IAG (G-SCF, low-dose Ara-c and idarubicin), or HMA with or without CAG or IAG. After CR, patients subsequently received allo-HSCT. | 90 | Therapies before allo-HSCT were used as the combination regimen in research group, not followed by allo-HSCT. | 91 |
| Jia(15) | 2019 | China | 2013.1-2016.12 | A retrospective research | - | 5 | 42 (14-72) | FLT3-ITD mutated AML | Allo-HSCT vs. non-HSCT | Allo-HSCT | 1. Induction therapy: <60 years old (1) The induction regimen was standard DA, IA, and HAA; (2) Secondary AML post MDS, AML with myelodysplastic changes, the induction regimens included CAG (Ara-c+aclarithromycin +G-CSF), DCAG (decitabine 20 mg/m^2^/d×5d+CAG). 2. Consolidation therapy: (1) Low-risk patients continue intermediate-dose Ara-c after CR, 2g/m^2^ once every 12 hours×3 days, a total of 2-4 times, for 6-8 courses. (2) Intermediate/high-risk patients receives allo-HSCT after 2 consolidation courses. 3. (1) Partial remission (PR): repeat the initial plan or use a rescue plan of induction regimen; (2) Non-remission (NR): choose CAG, HAA, FLAG or clinical trials, and some patients will be combined with sorafenib. | unknown | Therapies before allo-HSCT were used as the combination regimen in research group, not followed by allo-HSCT. | unknown |
| Huang(16) | 2019 | China | 2009.7-2018.3 | A retrospective research | - | 6 | - | FLT3 mutated AML with NPM1 mutation and normal karyotype | Allo-HSCT vs. non-HSCT | Allo-HSCT | Induction regimens included IA and HAA. Patients in CR were treated with another course of IA or HAA, and then all patients were treated with intermediate-dose Ara-c for 2–3 courses, coupled with standard dose chemotherapies composed of aclacinomycin, Ara-c, etoposide, harringtonine, idarubicin and mitoxantrone as consolidation therapies. When relapsed, patients were treated with FLAG (fludarabine/Ara-c/G-SCF) or the CLAG (cladribine/Ara-c/G-SCF), or decitabine+CAG (Ara-c, aclacinomycin and G-CSF) for re-induction therapy. 22 patients received allo-HSCT at CR1. | 22 | Therapies before allo-HSCT were used as the combination regimen in research group, not followed by allo-HSCT. | 31 |
| Xuan(17) | 2018 | China | 2012.1-2015.12 | A retrospective research | - | 8 | 35 (14-57); Sorafenib before allo-HSCT: 33 (16-55), Sorafenib after allo-HSCT: 37 (15-55); Sorafenib before and after allo-HSCT: 33 (16-54), No sorafenib: 34 (14-57) | Newly diagnosed FLT3-ITD mutated AML | Induction, consolidation, with maintenance post allo-HSCT, or not. | Sorafenib before allo-HSCT was defined as more than 30 days before allo-HSCT. The initial dose of sorafenib was generally 400 mg twice daily, and the dose was adjusted on the basis of suspected toxicity (dose range, 200-800 mg daily). | Conventional induction chemotherapy and postremission consoliation treatment. | 36 | Used as the combination regimen in research group | 50 |
| Papayannidis(18) | 2018 | Italy | 2004-2017 | A retrospective research | - | 7 | 59 (17-74); FLT3 inhibitors: 55.5 (24-74), Control: 60(17-72) | FLT3-ITD mutated, refractory or relapsed AML | Salvage therapy for refractory or relapsed AML | Paitents received single agents based on FLT3 inhibitors (sorafenib, ponatinib, quizartinib, gilteritinib, midostaurin). | Patients received chemotherapy (3+7 like regimens; 3+7 like regimens with the addition of a third agent; fludarabine-based regimens). | 36 | Used as the combination regimen in research group | 22 |
| Zhou(19) | 2018 | China | 2012.4-2017.7 | A retrospective research | - | 7 | 40 (4-71) | FLT3-ITD mutated AML with MLL gene aberrations | Allo-HSCT vs. non-HSCT | Allo-HSCT | Induction: DA, IA, AA (aclarithromycin, Ara-C), ADE, HAA and others; consolidation: similar to induction therapy and adds intermediate- and high-dose Ara-C or a regimen containing methotrexate (MTX). Relapsed or refractory patients use CAG, FLAG (fludarabine, Ara-C, G-CSF), decitabine + Ara-C, CLAG (cladribine, Ara-C, G-CSF) and other regimens. Finally, patients received allo-HSCT. | 16 | Therapies before allo-HSCT were used as the combination regimen in research group, not followed by allo-HSCT. | 18 |
| Qiu(20) | 2018 | China | 2010.7-2015.10 | A retrospective research | - | 7 | 44 (19-59) | FLT3-ITD mutated AML | Allo-HSCT vs. non-HSCT | Allo-HSCT | 3 + 7 regimen and consolidation therapies were given to all patients. If patients failed to achieve CR after 2 courses of induction therapy, a priming regimen or a decitabine-based regimen with or without sorafenib was given for reinduction. | 61 | Therapies before allo-HSCT were used as the combination regimen in research group, not followed by allo-HSCT. | 69 |
| Hills(21) | 2018 | UK | - | A retrospective research | UK NCRI AML15,16,17 | 5 | 51 (18-84) | FLT3-ITD mutated, refractory or relapsed AML | Allo-HSCT vs. non-HSCT | Allo-HSCT | Allo-HSCT | - | Non-HSCT | - |
| Choi(22) | 2018 | Korea | 2002.1-2016.9 | A retrospective research | - | 6 | - | FLT3-ITD mutated AML | Allo-HSCT vs. non-HSCT | Allo-HSCT | Patients who achieved CR received allo-HSCT. | 80 | Patients who achieved CR received four to six courses of consolidation chemotherapy. | 44 |
| Zhang(23) | 2017 | China | 2013.1-2013.8 | A retrospective research | - | 7 | 45 (7-76) | Newly diagnosed FLT3-ITD mutated AML | Induction and consolidation therapy | The dose of sorafenib therapy was generally 400 mg twice daily. | Induction: DA, IA, TA (Topotecan + Ara-C), MA with/without homoharringtonine; Elderly patients or infected patients received regimens based on LoDAC (10mg/m^2^), like CAG. If induction failed, patients received the same regimen or changed regimen. Patients in CR received consolidation, followed by maintenance therapy or allo-HSCT. | 22 | Used as the combination regimen in research group | 31 |
| Stone(24) | 2017 | USA and Canada etc. 17 countries | 2008.5-2011.10 | A phase III randomized, double-blind, placebo-controlled, multicenter trial | NCT00651261 | 7 | 47.9 (18.0-60.9); Midostaurin: 47.1(19-59.8), Control 48.6(18-60.9) | Newly diagnosed FLT3 mutated AML | Induction, consolidation and maintenance therapy after CR | Midostaurin was administered in a double-blind fashion, at a dose of 50 mg orally twice daily, on days 8 through 21. Midostaurin was not administered if the patient had a corrected QT interval above 500 msec or a grade 3 or 4 nonhematologic toxic effect. Consolidation: Midostaurin was administered at a dose of 50 mg orally twice daily on days 8 through 21. Patients who remained in remission after completion of consolidation therapy entered a maintenance phase in which they received midostaurin, administered at a dose of 50 mg orally twice daily, for twelve 28-day cycles. | Induction therapy: daunorubicin (60 mg/m^2^ IV injection, day1-3) and Ara-c (200 mg/ m^2^ IV infusion on day1-7). A BM examination was performed on day 21. If there was definitive evidence of clinically significant residual leukemia, a second cycle of induction therapy that was identical to the first, including midostaurin, was administered. Patients in CR after induction therapy received four 28-day cycles of consolidation therapy with high-dose Ara-c (3000 mg/m^2^, administered over a period of 3 hours every 12 hours on days 1, 3, and 5). | 360 | Placebo: 50 mg orally twice daily, on day8-21. Placebo was not used if the patient had a corrected QT interval above 500 msec or a grade 3 or 4 nonhematologic toxic effect. Consolidation: Placebo was administered at a dose of 50 mg orally twice daily on day8-21. Patients who remained in remission after completion of consolidation therapy entered a maintenance phase in which they received placebo, administered at a dose of 50 mg orally twice daily, for twelve 28-day cycles. Chemotherapy was used as research group. | 357 |
| Knapper(25) | 2017 | United Kingdom, Denmark, and New Zealand | 2007.1-2009.1 | A phase III randomized, double-blind, placebo-controlled, multicenter trial | ISRCTN 17161961, the UK MRC AML15 | 6 | Lestaurtinib: 48 (16-66), Control: 46 (16-65) | Newly diagnosed FLT3 mutated AML | Induction therapy | Lestaurtinib was commenced 2 days after chemotherapy and administered in cycles of up to 28 days, for a maximum of 4 cycles, being stopped at least 2 days before commencing the next course of chemotherapy. The initial dose was 80mg orally twice daily; if well-tolerated, an increase to a maximum dose of 100mg was permitted from cycle 2 onward. In case of additional toxicity, which was anticipated with the co-administration of azole antifungal drugs with CYP3A4 inhibitory activity, provision was made for a reduced dose of 40-60 mg. Patients receiving allo-HSCT continued lestaurtinib until 28 days after their last pretransplant course of chemotherapy, but did not receive further lestaurtinib after transplant. | Induction chemotherapy (courses 1-2) was with ADE (Arac/daunorubicin/etoposide), DA, or FLAG-Ida, with or without GO in course 1; Consolidation (courses 3-4) comprised high-dose Ara-c (1.5 g/m^2^ or 3 g/m^2^) or MACE/MidAC. Allo-HSCT was permitted for patients with intermediate- or poor-risk disease with a recommendation of myeloablative conditioning for patients younger than 35 years and reduced-intensity conditioning for patients older than 45 years, with investigator/patient choice in the intermediate age group in AML15, but was recommended only for poor-risk patients in AML17. In neither trial was FLT3 status an indication for transplant. | 88 | Used as the combination regimen in research group | 87 |
| Knapper(25) | 2017 | United Kingdom, Denmark, and New Zealand | 2009.4-2012.10 | A phase III randomized, double-blind placebo-controlled, multicenter trial | ISRCTN 55675535, NCRI AML 17 study | 6 | Lestaurtinib: 50 (5-68), Control: 50 (6-65) | Newly diagnosed FLT3 mutated AML | Induction therapy | As above | As above | 212 | As above | 113 |
| Ahmed(26) | 2017 | US | 2010.1-2016.10 | A retrospective research | - | 7 | - | FLT3-ITD mutated AML after allo-HSCT | Maintenance therapy post allo-HSCT | The most commonly administered dose for sorafenib post allo-HSCT was 400 mg daily (5 patients) for 28 days cycle; only 2 patients tolerated higher doses and 6 patients received 300mg daily or less. | - | 13 | - | 26 |
| Brunner(27) | 2016 | USA | 2008.1-2014.12 | A retrospective research | - | 8 | Sorafenib: 55 (20-74), Control: 56 (25-73) | FLT3-ITD mutated AML after allo-HSCT | Maintenance therapy post allo-HSCT | Sorafenib was dosed 200-400 mg twice daily, and patients were assessed to initiate therapy starting at day +45 after transplant and onward. The maximum tolerated dose was 400 mg twice a day. Among patients treated with sorafenib, 6 started at 200 mg twice daily, 3 were treated with 400 mg in the morning and 200mg in the evening, and 17 started at 400 mg twice daily. | Induction: IA, DA, MA; Consolidation: none; High/Intermediate-dose Ara-c; sorafenib-based; 26 cases received sorafenib post allo-HSCT. | 26 | Used as the combination regimen in research group; 55 patients didn't receive sorafenib post allo-HSCT. | 55 |
| Oran(28) | 2016 | USA | 2000.7-2013.11 | A retrospective research | - | 7 | 59 (50-68); allo-HSCT: 55 (47-62); Control: 62(52-70) | FLT3-ITD mutated AML | Allo-HSCT vs. non-HSCT | Allo-HSCT | Induction: high-dose Ara-c-based regimens, HMAs, clofarabine and cladrabine. Most patients aged 65 years received high-dose Ara-c-based regimen. All high-risk patients received allo-HSCT. | 48 | Therapies before allo-HSCT were used as the combination regimen in research group. Patients without HSCT received consolidation therapy similar to induction therapy. | 121 |
| Ho(29) | 2016 | Germany | 2003.12-2009.12 | A retrospective research | NCT00180102 | 7 | 49 (20-60) | FLT3-ITD mutated AML | Allo-HSCT vs. non-HSCT | Allo-HSCT | All patients received the “7+3” regimen with DA. And 109 patients were scheduled for allo-HSCT. | 109 | Therapies before allo-HSCT were used as the combination regimen in research group, not followed by allo-HSCT. | 100 |
| Roellig(30) | 2015 | Germany | 2009.3-2011.11 | A phase II randomised, double-blind, placebo-controlled trial | NCT00893373 | 7 | Sorafenib: 50 (43–56), Control: 50 (44–55) | Newly diagnosed AML | Induction, consolidation and maintenance therapy after CR | For induction regimen, patients received 400mg sorafenib, twice daily on days 10–19. For consolidation therapy, sorafenib were given from day 8 until 3 days before the next consolidation cycle. Sorafenib 400 mg twice daily was given continuously for 12 months after the last consolidation cycle. In case of grade 3 or worse toxicity, dose reductions to 400 mg sorafenib once a day or 400 mg every other day were allowed. | Induction: DA (Ara-c 100 mg/m² daily day1-7, daunorubicin 60 mg/m² day3–5). Patients with a reduction in BM blast or cellularity on early response assessment on day16 under took a second induction from day22, whereas non-responders received high-dose Ara-c 3h 3 g/m² twice daily on day1–3 plus mitoxantrone 10 mg/m² on day3–5. Once patient achieved CR, intermediate-risk patients with a sibling donor and high-risk patients with a matched donor were offered an allo-HSCT whereas all other patients proceeded to three cycles of Ara-c-based consolidation with Ara-c 3h, 3 g/m² twice daily on days 1, 3, and 5. | 134 | Used as the combination regimen in research group | 133 |
| Badar(31) | 2015 | USA | 2000-2014 | A retrospective research | - | 6 | 61 (17-89) | FLT3-ITD mutated AML | Allo-HSCT vs. non-HSCT | Allo-HSCT | Unknown | 77 | Non-HSCT | 147 |
| Schlenk(32) | 2014 | German-Austrian | 1993-2009 | A retrospective research | NCT00151242; AML HD9329; AML HD98A30; AMLSG 07-0431 | 6 | - | FLT3-ITD mutated AML | Allo-HSCT vs. non-HSCT | Allo-HSCT | Unknown | 45 | Non-HSCT | 18 |
| Serve(33) | 2013 | Germany | 2006.9-2008.6 | A phase II randomized, double-blind, placebo-controlled, multicenter trial | NCT00373373 | 7 | 68 (61-80); Sorafenib: 67.5(61-78), Control: 69(61-80) | Newly diagnosed AML | Induction, consolidation and maintenance therapy after CR | Patients received sorafenib 400 mg twice daily, from day 3 after end of induction chemotherapy and each consolidation course continuously until 3 days before the first day of the next chemotherapy course. Maintenance therapy was given to all patients in CR after consolidation as sorafenib 400 mg twice daily, starting on day3 after the end of the last consolidation course until 1 year after start of induction therapy. | Induction therapy was a combination of Ara-c (100 mg/m^2^ per day as continuous intravenous infusion on day1-7) and daunorubicin (60 mg/m^2^ per day intravenously on day3-5). Patients in CR received consolidation therapy administered as two courses of intermediate/high-dose Ara-c (1g/m^2^, every 12 hours, on days 1, 3, and 5) not earlier than 1 week after attaining CR. If the patient’s condition did not allow for consolidation therapy, it was possible to postpone chemotherapy for up to 6 weeks after CR was reached. If consolidation therapy could not be applied within 6 weeks after CR, the patient went on to maintenance therapy. | 102 | Patients received two placebo tablets twice daily, from day 3 after end of induction chemotherapy and each consolidation course continuously until 3 days before the first day of the next chemotherapy course. Maintenance therapy was given to all patients in CR after consolidation as placebo tablets twice daily, starting on day 3 after the end of the last consolidation course until 1 year after start of induction therapy. Induction therapy, consolidation therapy and maintenance therapy were used as the combination regimens in research group. | 95 |
| Takahashi (1)(34) | 2013 | USA | 1995.8 2011.6 | A retrospective research | - | 6 | FLT3 inhibitors: 55 ± 2.6, Control: 51 ± 1.8 | FLT3-ITD mutated, refractory or relapsed AML | Salvage therapy for refractory or relapsed AML | The most common FLT3 inhibitors used as salvage regimen were lestaurtinib (alone or combination) and sorafenib (alone or combination). Other patients in the FLT3i group received quizartinib (alone), KW-2449 (alone), ponatinib (alone) or midostaurin (alone or combination). | CEP-701+VP16 + Mitoxantrone + Ara-C/high-dose Ara-C; Sorafenib + IA/Plerixafor/Plerixafor + G-CSF; midostaurin+5-Aza. | 45 | IA, high-dose Ara-C; FA included fludarabine + Ara-C; DCTER included daunorubicin + Ara-C + topotecan + etoposide + dexamethasone; CAT included cyclophosphamide + Ara-C + topotecan; FAI included fludarabine + Ara-C + idarubicin; Other regimens included cloretazine-based regimens, hypomethylator containing and clofarabin-based regimens | 75 |
| Takahashi (2)(34) | 2013 | USA | 1995.8-2011.6 | A retrospective research | - | 6 | - | FLT3-ITD mutated, refractory or relapsed AML | Allo-HSCT vs. non-HSCT | Allo-HSCT | Unknown | 33 | Non-HSCT | 87 |
| Lin(35) | 2013 | China | 2003.1-2010.12 | A retrospective research | - | 6 | 46.1 ± 10.3 | Newly diagnosed FLT3-ITD mutated AML | Allo-HSCT vs. non-HSCT | Allo-HSCT | Induction: anthracycline for 3 days, Ara-c 100–200 mg/m^2^/day on day1–7. Patients in CR received high-dose Ara-c (2–3 g/m^2^/day on days 1, 3 and 5) with/without anthracycline, as consolidation therapy. Salvage therapy for relapsed AML: MEC (mitoxantrone 8 mg/m^2^ on day1-3, etoposide 100 mg/m^2^ on day1–5, and Ara-c 75 mg/m^2^/ twice a day on day1–5), FLAG (fludarabine 30 mg/m^2^ on day1–5, Ara-c 2 g/m^2^ on day1–5 and G-CSF 300 ug daily on day0–6), as well as N3A7 (mitoxanthrone 8 mg/m^2^ on day1–3 and Ara-c 100–200 mg/m^2^/day on day1–7) and N3-HDAC (mitoxanthrone 8 mg/m^2^ on day1–3 and HDAC). If a suitable donor was available, patients receive allo-HSCT under CR or at least a good partial response. | 10 | Therapies before allo-HSCT were used as the combination regimen in research group, not followed by allo-HSCT. | 24 |
| Levis(36) | 2011 | USA, Australia, Canada, Israel, Europe-related countries | 2004.1 -2008.12 | A phase II randomized, open-label, multicenter trial | NCT00079482 | 4 | Lestaurtinib: 59 (20-81), Control: 54 (21-79) | FLT3-ITD mutated, refractory or relapsed AML | Salvage therapy for refractory or relapsed AML | Lestaurtinib was administered at a dose of 80 mg orally twice daily (12 hours between doses), beginning 2 days after the completion of chemotherapy (day 7). | Patients in CR1 lasting from 1 to 6 months received MEC, consisting of mitoxantrone 8 mg/m^2^/day, etoposide 100 mg/m^2^, and Ara-c 1000 mg/m^2^/day IV on day1-5. Patients in CR1 lasted from 6 to 24 months received HiDAC, consisting of Ara-c 1500 mg/m^2^ daily on day1-5. | 112 | Used as the combination regimen in research group | 112 |
| Bornhaeuser(37) | 2007 | Germany | 1996-2003 | A retrospective research | - | 6 | - | Newly diagnosed FLT3-ITD(+) AML | Allo-HSCT vs. non-HSCT | Allo-HSCT | Induction: 2 cycles of high-dose Ara-C and allo-HSCT using an HLA-matched sibling donor was performed after conditioning therapy. | 40 | Therapies before allo-HSCT were used as the combination regimen in research group, not followed by allo-HSCT. | 38 |

Abbreviations: AML, acute myeloid leukemia; FLT3-ITD, FMS-like tyrosine kinase 3-internal tandem duplication; allo-HSCT, allogeneic hematopoietic stem cell transplant; CR, complete remission; CRi, CR with incomplete blood count recovery; Ara-c, Cytarabine; CIA, clofarabine, idarubicin, and Ara-c; CLIA, cladribine, idarubicin and Ara-c; FIA, fludarabine, idarubicin, Ara-c; IA, idarubicin and Ara-c; HMA, hypomethylating agents; HAA, homoharringtonine, aclamycin and Ara-c; GO, gemtuzumab ozogamicin; IV, intravenous; DLI, donor lymphocyte infusion; G-CSF, granulocyte-colony stimulating factor; LoDAC, low-dose Ara-c; SC, subcutaneous; MEC, mitoxantrone, etoposide and Ara-c; FLAG-IDA, G-CSF, fludarabine, Ara-c and idarubicin; DA, daunorubicin and Ara-C; ADE, daunorubicin, Ara-C, etoposide; HAA, MA, mitoxantrone and Ara-C; BM, bone marrow; CAG, G-SCF, Ara-c and aclarubicin; NPM1, nucleophosmin 1; HAA, homoharringtonine, aclamycin and Ara-c; MACE/MidAC, amsacrine, cytarabine, etoposide, and mitoxantrone/cytarabine.

**References**

1. Xuan L, Wang Y, Huang F, Fan Z, Xu Y, Sun J*,* et al. Sorafenib maintenance in patients with FLT3-ITD acute myeloid leukaemia undergoing allogeneic haematopoietic stem-cell transplantation: an open-label, multicentre, randomised phase 3 trial. *Lancet Oncol.* (2020) 21:1201-12. doi: 10.1016/S1470-2045(20)30455-1

2. Ofran Y, Leiba R, Frisch A, Horesh N, Henig I, Yehudai‐Ofir D, et al. Midostaurin in combination with chemotherapy is most effective in patients with acute myeloid leukemia presenting with high FLT3‐ITD allelic ratio who proceed to allogeneic stem cell transplantation while in first complete remission. *Eur J Haematol.* (2020) 106:64-71. doi: 10.1111/ejh.13518

3. Burchert A, Bug G, Fritz LV, Finke J, Stelljes M, Röllig C, et al. Sorafenib maintenance after allogeneic hematopoietic stem cell transplantation for acute myeloid leukemia with FLT3–Internal Tandem Duplication Mutation (SORMAIN). *J Clin Oncol.* (2020) 38:2993-3002. doi: 10.1200/JCO.19.03345

4. Bazarbachi A, Labopin M, Battipaglia G, Djabali A, Forcade E, Arcese W, et al. Allogeneic stem cell transplantation for FLT3-mutated acute myeloid leukemia: in vivo T-cell depletion and posttransplant sorafenib maintenance improve survival. A retrospective acute Leukemia Working Party-European Society for Blood and Marrow Transplant Study. *Clin Hematol Int.* (2019) 1:58-74. doi: 10.2991/chi.d.190310.001

5. Bazarbachi A, Labopin M, Battipaglia G, Djabali A, Passweg J, Socie G, et al. Sorafenib improves survival of FLT3-mutated acute myeloid leukemia in relapse after allogeneic stem cell transplantation: a report of the EBMT Acute Leukemia Working Party. *Haematologica.* (2019) 104:e398-e401. doi: 10.3324/haematol.2018.211615

6. Sasaki K, Kantarjian HM, Kadia T, Patel K, Loghavi S, Garcia-Manero G, et al. Sorafenib plus intensive chemotherapy improves survival in patients with newly diagnosed, FLT3-internal tandem duplication mutation-positive acute myeloid leukemia. *Cancer.* (2019) 125:3755-66. doi: 10.1002/cncr.32387

7. Schlenk RF, Weber D, Fiedler W, Salih HR, Wulf G, Salwender H, et al. Midostaurin added to chemotherapy and continued single-agent maintenance therapy in acute myeloid leukemia with FLT3-ITD. *Blood.* (2019) 133:840-51. doi: 10.1182/blood-2018-08-869453

8. Berger T, Rozovski U, Moshe Y, Yaari S, Frisch A, Hellmann I, et al. Midostaurin in combination with intensive chemotherapy is safe and associated with improved remission rates and higher transplantation rates in first remission-a multi-center historical control study. *Ann Hematol.* (2019) 98:2711-17. doi: 10.1007/s00277-019-03795-8

9. Chappell G, Geer M, Gatza E, Braun T, Churay T, Brisson J, et al. Maintenance sorafenib in FLT3-ITD AML following allogeneic HCT favorably impacts relapse and overall survival. *Bone Marrow Transplant.* (2019) 54:1518-20. doi: 10.1038/s41409-019-0493-5

10. Maziarz RT, Fernandez H, Patnaik MM, Scott BL, Mohan S, Deol A, et al. Radius: Midostaurin (mido) plus standard of care (SOC) after allogeneic stem cell transplant (alloSCT) in patients (pts) with FLT3-internal tandem duplication (ITD)–mutated acute myeloid leukemia (AML). *Biol Blood Marrow Transplant.* (2019) 25:S11-S2. doi: 10.1016/j.bbmt.2018.12.077

11. Xuan L, Wang Y, Chen J, Jiang E, Gao L, Wu B, et al. Sorafenib therapy is associated with improved outcomes for FMS-like tyrosine kinase 3 internal tandem duplication acute myeloid leukemia relapsing after allogeneic hematopoietic stem cell transplantation. *Biol Blood Marrow Transplant.* (2019) 25:1674-81. doi: 10.1016/j.bbmt.2019.04.018

12. Perl AE, Martinelli G, Cortes JE, Neubauer A, Berman E, Paolini S, et al. Gilteritinib or chemotherapy for relapsed or refractory FLT3-mutated AML. *N Engl J Med.* (2019) 381:1728-40. doi: 10.1056/NEJMoa1902688

13. Cortes JE, Khaled S, Martinelli G, Perl AE, Ganguly S, Russell N, et al. Quizartinib versus salvage chemotherapy in relapsed or refractory FLT3-ITD acute myeloid leukaemia (QuANTUM-R): a multicentre, randomised, controlled, open-label, phase 3 trial. *Lancet Oncol.* (2019) 20:984-97. doi: 10.1016/s1470-2045(19)30150-0

14. Wang H, Chu TT, Han SY, Qi JQ, Tang YQ, Qiu HY, et al. FLT3-ITD and CEBPA mutations predict prognosis in acute myelogenous leukemia irrespective of hematopoietic stem cell transplantation. *Biol Blood Marrow Transplant.* (2019) 25:941-8. doi: 10.1016/j.bbmt.2018.11.031

15. Jia J, Zhu H, Gong L, Zhao T, Wang J, Jiang Q, et al. Analysis of induction efficacy and prognostic factors in FLT3-ITD positive acute myeloid leukemia in the real world. *Chinese Journal of Hematology.* (2019) 40:398-403. doi: 10.3760/cma.j.issn.0253-2727.2019.05.010

16. Huang Y, Hu J, Lu T, Luo Y, Shi J, Wu W, et al. Acute myeloid leukemia patient with FLT3-ITD and NPM1 double mutation should undergo allogeneic hematopoietic stem cell transplantation in CR1 for better prognosis. *Cancer Manag Res.* (2019) 11:4129-42. doi: 10.2147/cmar.s194523

17. Xuan L, Wang Y, Huang F, Jiang E, Deng L, Wu B, et al. Effect of sorafenib on the outcomes of patients with FLT3-ITD acute myeloid leukemia undergoing allogeneic hematopoietic stem cell transplantation. *Cancer.* (2018) 124:1954-63. doi: 10.1002/cncr.31295

18. Papayannidis C, Marconi G, De Polo S, Ottaviani E, Paolini S, Bochicchio M, et al. Tyrosine kinase inhibitors (TKI) in relapsed/refractory (rr) patients with FLT3-ITD positive acute myeloid leukemia (AML) confer better survival than chemotherapy, due to a better safety profile. *Clin Lymphoma Myeloma Leuk.* (2018) 18(Supplement 1):S200. doi: 10.1016/j.clml.2018.07.048

19. Zhou JR, Zhang X, Zhao YL, Yang JF, Zhang JP, Cao XY, et al. [Clinical characteristics and prognosis of 34 cases of acute myeloid leukemia with FLT3 internal tandem duplication and MLL gene rearrangement]. *Chinese Journal of Hematology.* (2018) 39:751-6. doi: 10.3760/cma.j.issn.0253-2727.2018.09.010

20. Qiu QC, Wang C, Bao XB, Yang J, Shen HJ, Ding ZX, et al. The impact of FLT3 mutations on treatment response and survival in Chinese de novo AML patients. *Hematology (Amsterdam, Netherlands).* (2018) 23:131-8. doi: 10.1080/10245332.2017.1372248

21. Hills RK, Burnett AK, Gale R, Linch DC, Gilkes A, Russell NH. Outcomes in relapsed/refractory patients with FLT3-ITD mutated AML are poor when treated with non-targeted therapy with a potential role for stem cell transplantation: results from the NCRI AML trials. Am Soc Hematology; (2018).

22. Choi EJ, Lee JH, Lee JH, Park HS, Ko SH, Hur EH, et al. Comparison of anthracyclines used for induction chemotherapy in patients with FLT3-ITD-mutated acute myeloid leukemia. *Leuk Res.* (2018) 68:51-6. doi: 10.1016/j.leukres.2018.03.006

23. Zhang QY, Wei XD, Yin QS, Mi RH, Yuan FF, Chen L. [Sorafenib in combination with chemotherapy as first-line therapy for FLT3-ITD positive acute myeloid leukemia]. *Chinese Journal of Hematology.* (2017) 38:415-20. doi: 10.3760/cma.j.issn.0253-2727.2017.05.012

24. Stone RM, Mandrekar SJ, Sanford BL, Laumann K, Geyer S, Bloomfield CD, et al. Midostaurin plus chemotherapy for acute myeloid leukemia with a FLT3 mutation. *N Engl J Med.* (2017) 377:454-64. doi: 10.1056/NEJMoa1614359

25. Knapper S, Russell N, Gilkes A, Hills RK, Gale RE, Cavenagh JD, et al. A randomized assessment of adding the kinase inhibitor lestaurtinib to first-line chemotherapy for FLT3-mutated AML. *Blood.* (2017) 129:1143-54. doi: 10.1182/blood-2016-07-730648

26. Ahmed S, Saliba R, Rondon G, Alousi A, Bashir Q, Ciurea S, et al. Sorafenib maintenance in Flt3-Itd mutated acute myeloid leukemia after allogeneic stem cell transplant. (2017). FERRATA STORTI FOUNDATION VIA GIUSEPPE BELLI 4, 27100 PAVIA, ITALY. p 323-4.

27. Brunner AM, Li S, Fathi AT, Wadleigh M, Ho VT, Collier K, et al. Haematopoietic cell transplantation with and without sorafenib maintenance for patients with FLT3-ITD acute myeloid leukaemia in first complete remission. *Br J Haematol.* (2016) 175:496-504. doi: 10.1111/bjh.14260

28. Oran B, Cortes J, Beitinjaneh A, Chen HC, de Lima M, Patel K, et al. Allogeneic transplantation in first remission improves outcomes irrespective of FLT3-ITD allelic ratio in FLT3-ITD-positive acute myelogenous leukemia. *Biol Blood Marrow Transplant.* (2016) 22:1218-26. doi: 10.1016/j.bbmt.2016.03.027

29. Ho AD, Schetelig J, Bochtler T, Schaich M, Schafer-Eckart K, Hanel M, et al. Allogeneic stem cell transplantation improves survival in patients with acute myeloid leukemia characterized by a high allelic ratio of mutant FLT3-ITD. *Biol Blood Marrow Transplant.* (2016) 22:462-9. doi: 10.1016/j.bbmt.2015.10.023

30. Rollig C, Serve H, Huttmann A, Noppeney R, Muller-Tidow C, Krug U, et al. Addition of sorafenib versus placebo to standard therapy in patients aged 60 years or younger with newly diagnosed acute myeloid leukaemia (SORAML): a multicentre, phase 2, randomised controlled trial. *Lancet Oncol.* (2015) 16:1691-9. doi: 10.1016/s1470-2045(15)00362-9

31. Badar T, Kantarjian HM, Nogueras‐Gonzalez GM, Borthakur G, Garcia Manero G, Andreeff M, et al. Improvement in clinical outcome of FLT3 ITD mutated acute myeloid leukemia patients over the last one and a half decade. *Am J Hematol.* (2015) 90:1065-70. doi: 10.1002/ajh.24140

32. Schlenk RF, Kayser S, Bullinger L, Kobbe G, Casper J, Ringhoffer M, et al. Differential impact of allelic ratio and insertion site in FLT3-ITD-positive AML with respect to allogeneic transplantation. *Blood.* (2014) 124:3441-9. doi: 10.1182/blood-2014-05-578070

33. Serve H, Krug U, Wagner R, Sauerland MC, Heinecke A, Brunnberg U, et al. Sorafenib in combination with intensive chemotherapy in elderly patients with acute myeloid leukemia: results from a randomized, placebo-controlled trial. *J Clin Oncol.* (2013) 31:3110-8. doi: 10.1200/jco.2012.46.4990

34. Takahashi K, Kantarjian H, Pemmaraju N, Andreeff M, Borthakur G, Faderl S, et al. Salvage therapy using FLT3 inhibitors may improve long-term outcome of relapsed or refractory AML in patients with FLT3-ITD. *Br J Haematol.* (2013) 161:659-66. doi: 10.1111/bjh.12299

35. Lin PH, Lin CC, Yang HI, Li LY, Bai LY, Chiu CF, et al. Prognostic impact of allogeneic hematopoietic stem cell transplantation for acute myeloid leukemia patients with internal tandem duplication of FLT3. *Leuk Res.* (2013) 37:287-92. doi: 10.1016/j.leukres.2012.10.005

36. Levis M, Ravandi F, Wang ES, Baer MR, Perl A, Coutre S, et al. Results from a randomized trial of salvage chemotherapy followed by lestaurtinib for patients with FLT3 mutant AML in first relapse. *Blood.* (2011) 117:3294-301. doi: 10.1182/blood-2010-08-301796

37. Bornhauser M, Illmer T, Schaich M, Soucek S, Ehninger G, Thiede C. Improved outcome after stem-cell transplantation in FLT3/ITD-positive AML. *Blood.* (2007) 109:2264-5. doi: 10.1182/blood-2006-09-047225

**Supplementary Table 3. NOS score in 28 retrospective studies.**

| **Study** | **Selection** | | | | **Comparability** | **Outcome** | | | **Score** |
| --- | --- | --- | --- | --- | --- | --- | --- | --- | --- |
|  | **Representativeness of the exposed cohort** | **Selection of the non-exposed cohort** | **Ascertainment of exposure** | **Demonstration that outcome of interest was not present at start of study** | **Comparability of cohorts on the basis of the design or analysis** | **Assessment of outcome** | **Was follow-up long enough for outcomes to occur (5 years)** | **Adequacy of follow up of cohorts** |  |
| Ofran (2020) | ☆ | ☆ | ☆ | ☆ | ☆☆ | ☆ | ☆ | - | 8 |
| Bazarbachi (1)(2019) | ☆ | ☆ | ☆ | ☆ | - | ☆ | ☆ | - | 6 |
| Bazarbachi (2)(2019) | ☆ | ☆ | ☆ | ☆ | ☆☆ | ☆ | - | - | 7 |
| Sasaki (2019) | ☆ | ☆ | ☆ | ☆ | ☆☆ | ☆ | ☆ | ☆ | 9 |
| Xuan (2018) | ☆ | ☆ | ☆ | ☆ | ☆☆ | ☆ | ☆ | - | 8 |
| Zhang (2017) | ☆ | ☆ | ☆ | ☆ | ☆☆ | ☆ | - | - | 7 |
| Schlenk (2019) | ☆ | ☆ | ☆ | ☆ | ☆ | ☆ | ☆ | - | 7 |
| Berger (2019) | ☆ | ☆ | ☆ | ☆ | ☆☆ | ☆ | - | - | 7 |
| Chappell (2019) | ☆ | ☆ | ☆ | ☆ | - | ☆ | - | - | 5 |
| Ahmed (2017) | ☆ | ☆ | ☆ | ☆ | ☆☆ | ☆ | - | - | 7 |
| Brunner (2016) | ☆ | ☆ | ☆ | ☆ | ☆☆ | ☆ | - | ☆ | 8 |
| Xuan (2019) | ☆ | ☆ | ☆ | ☆ | ☆☆ | ☆ | ☆ | - | 8 |
| Papayannidis (2018) | ☆ | ☆ | ☆ | ☆ | ☆☆ | ☆ | - | - | 7 |
| Wang (2019) | ☆ | ☆ | ☆ | ☆ | - | ☆ | ☆ | - | 6 |
| Jia (2019) | ☆ | ☆ | ☆ | ☆ | - | ☆ | - | - | 5 |
| Huang (2019) | ☆ | ☆ | ☆ | ☆ | - | ☆ | - | ☆ | 6 |
| Zhou (2018) | ☆ | ☆ | ☆ | ☆ | ☆ | ☆ | - | ☆ | 7 |
| Qiu (2018) | ☆ | ☆ | ☆ | ☆ | - | ☆ | ☆ | ☆ | 7 |
| Hills (2018) | ☆ | ☆ | ☆ | ☆ | - | ☆ | - | - | 5 |
| Choi (2018) | ☆ | ☆ | ☆ | ☆ | - | ☆ | ☆ | - | 6 |
| Oran (2016) | ☆ | ☆ | ☆ | ☆ | - | ☆ | ☆ | ☆ | 7 |
| Ho (2016) | ☆ | ☆ | ☆ | ☆ | - | ☆ | ☆ | ☆ | 7 |
| Badar (2015) | ☆ | ☆ | ☆ | ☆ | - | ☆ | ☆ | - | 6 |
| Schlenk (2014) | ☆ | ☆ | ☆ | ☆ | - | ☆ | ☆ | - | 6 |
| Takahashi (1) (2013) | ☆ | ☆ | ☆ | ☆ | - | ☆ | - | ☆ | 6 |
| Takahashi (2) (2013) | ☆ | ☆ | ☆ | ☆ | - | ☆ | - | ☆ | 6 |
| Lin (2013) | ☆ | ☆ | ☆ | ☆ | - | ☆ | ☆ | - | 6 |
| Bornhaeuser (2007) | ☆ | ☆ | ☆ | ☆ | - | ☆ | ☆ | - | 6 |

**Supplementary Table 4. Summarized effects of FLT3i in various treatment stages before and after sensitivity analyses.**

|  |  | **Results before sensitive analyses** | | | | | | | | **Results after sensitive analyses** | | | | | | | | **Source of heterogeneity** | |
| --- | --- | --- | --- | --- | --- | --- | --- | --- | --- | --- | --- | --- | --- | --- | --- | --- | --- | --- | --- |
|  | **Groups** | **FLT3i**  **or allo-HSCT** | **Control** | **Pooled**  **RR or HR** | **Lower 95%CI** | **Upper 95%CI** | ***p*** | ***I^2^*** | ***P* of heterogeneity** | **FLT3i**  **or allo-HSCT** | **Control** | **Pooled**  **RR or HR** | **Lower 95%CI** | **Upper 95%CI** | ***p*** | ***I^2^*** | ***P* of heterogeneity** | **Study** | **Reason** |
| Induction treatment in newly diagnosed AML | OS | 1157 | 1060 | 0.76 | 0.67 | 0.87 | < 0.01 | 0.0% | 0.460 | - | - | - | - | - | - | - | - | - | - |
|  | EFS | 959 | 1104 | 0.74 | 0.56 | 0.99 | 0.04 | 90.5% | 0.000 | 857 | 1009 | 0.67 | 0.58 | 0.78 | < 0.01 | 43.1% | 0.153 | Serve, 2013 | 1. The patient cohort from this study contained all of AML, not limited to FLT3 (+) AML; 2. The patient cohort was limited to elderly AML (>60 years old). |
|  | RFS | 715 | 613 | 0.64 | 0.50 | 0.82 | < 0.01 | 58.4% | 0.025 | 669 | 576 | 0.72 | 0.60 | 0.85 | < 0.01 | 14.3% | 0.323 | Xuan, 2018 | The median age of patient cohort from this research was the youngest when compared to other studies. |
|  | CIR | 577 | 534 | 0.78 | 0.60 | 1.02 | 0.07 | 42.2% | 0.140 | - | - | - | - | - | - | - | - | - | - |
| Allo-HSCT in FLT3 (+) AML | OS^a^ | 631 | 788 | 0.52 | 0.39 | 0.68 | < 0.01 | 76.9% | 0.000 | 593 | 739 | 0.53 | 0.45 | 0.64 | < 0.01 | 35.5% | 0.115 | Huang, 2019, Zhou, 2018 and Hills, 2018 | 1. Huang 2019 focused on patient coexisting FLT3 and NPM1 mutations with normal cytogenetics; 2. Zhou 2018 focused on FLT3-ITD mutated AML with MLL gene aberrations, receiving allo-HSCT or not; 3. Hills 2018 focused on adult rrAML patients with FLT3-ITD mutation. |
|  | EFS | 189 | 144 | 0.50 | 0.33 | 0.77 | < 0.01 | 0.0% | 0.974 | - | - | - | - | - | - | - | - | - | - |
|  | RFS^a^ | 485 | 496 | 0.49 | 0.37 | 0.65 | < 0.01 | 59.2% | 0.012 | 448 | 450 | 0.57 | 0.45 | 0.71 | < 0.01 | 38.5% | 0.135 | Huang, 2019 and Zhou, 2018 | 1. Huang 2019 focused on patient coexisting FLT3 and NPM1 mutations with normal cytogenetics; 2. Zhou 2018 focused on FLT3-ITD mutated AML with MLL gene aberrations, receiving allo-HSCT or not. |
|  | CIR^a^ | 120 | 82 | 0.26 | 0.18 | 0.38 | < 0.01 | 0.0% | 0.788 | - | - | - | - | - | - | - | - | - | - |
| Maintenance treatment after allo-HSCT | OS | 301 | 792 | 0.45 | 0.34 | 0.60 | < 0.01 | 0.0% | 0.648 | - | - | - | - | - | - | - | - | - | - |
|  | RFS | 238 | 694 | 0.34 | 0.24 | 0.47 | < 0.01 | 0.0% | 0.994 | - | - | - | - | - | - | - | - | - | - |
|  | CIR | 241 | 713 | 0.32 | 0.21 | 0..46 | < 0.01 | 0.0% | 0.875 | - | - | - | - | - | - | - | - | - | - |
| Salvage therapy in FLT3(+) rrAML | OS | 770 | 596 | 0.65 | 0.50 | 0.83 | < 0.01 | 62.7% | 0.013 | 658 | 484 | 0.60 | 0.49 | 0.74 | < 0.01 | 29.6% | 0.213 | Levis, 2011 | This study focused on lestaurtinib, which did not work in all of trials listed in in our study. |
|  | EFS | 492 | 246 | 0.86 | 0.70 | 1.04 | 0.14 | 0.0% | 0.549 | - | - | - | - | - | - | - | - | - | - |
|  | RFS | 105 | 43 | 0.40 | 0.21 | 0.75 | 0.01 | 0.0% | 0.544 | - | - | - | - | - | - | - | - | - | - |
| Response (CR) | Induction treatment in newly diagnosed AML | 826/1082 (76.3%) | 783/1128 (69.4%) | 0.88 | 0.78 | 0.99 | 0.04 | 63.9% | 0.005 | - | - | - | - | - | - | - | - | - | No source for heterogeneity in the sensitivity analyses |
|  | Salvage therapy in FLT3(+) rrAML | 284/738 (38.5%) | 111/485 (22.9%) | 0.66 | 0.48 | 0.90 | 0.01 | 54.4% | 0.052 | 278/702 (39.6%) | 105/463 (22.7%) | 0.61 | 0.46 | 0.81 | < 0.01 | 44.3% | 0.126 | Papayannidis, 2018 | In this study, patients received single agent based on FLT3i (sorafenib, ponatinib, quizartinib, gilteritinib, midostaurin), which was different from other studies. |

^a^Studies from Jia (2019) and Hills (2018) did not report the cohort size of each group.

Abbreviations: FLT3, FMS-like tyrosine kinase 3; FLT3i, FLT3 inhibitor; allo-HSCT, allogeneic hematopoietic stem cell transplant; RR, relative risk; HR, hazard ratio; 95% CI, 95% confidence interval; AML, acute myeloid leukemia; OS, overall survival; EFS, event-free survival; RFS, relapse-free survival; CIR, cumulative incidence of relapse; CR, complete remission; rrAML, refractory and relapsed acute myeloid leukemia; NPM1, nucleophosmin 1, FLT3-ITD, FMS-like tyrosine kinase 3-internal tandem duplication; MLL, mixed lineage leukemia.

**Supplementary Table 5. Effects of various FLT3i on AML in different treatment stages.**

|  |  | **Induction treatment in newly diagnosed AML** | | | | | | | **Maintenance treatment after allo-HSCT** | | | | | | | **Salvage therapy in FLT3(+) rrAML** | | | | | | |
| --- | --- | --- | --- | --- | --- | --- | --- | --- | --- | --- | --- | --- | --- | --- | --- | --- | --- | --- | --- | --- | --- | --- |
| **Group** | **Endpoints** | **FLT3i** | **Control** | **HR/RR** | **95% CI** | ***p*** | ***I^2^*** | ***P* of heterogeneity** | **FLT3i** | **Control** | **HR/RR** | **95% CI** | ***p*** | ***I^2^*** | ***P* of heterogeneity** | **FLT3i** | **Control** | **HR/RR** | **95% CI** | ***p*** | ***I^2^*** | ***P* of heterogeneity** |
| Sorafenib | OS | 399 | 413 | 0.68 | 0.51-0.91 | 0.01 | 13.3% | 0.330 | 271 | 762 | 0.44 | 0.33-0.59 | < 0.01 | 0.0% | 0.557 | 87 | 148 | 0.48 | 0.32-0.71 | < 0.01 | 0.0% | 0.662 |
|  | EFS | 315 | 332 | 0.80 | 0.54-1.19 | 0.27 | 88.3% | 0.000 | - | - | - | - | - | - | - | - | - | - | - | - | - | - |
|  | RFS | 227 | 234 | 0.49 | 0.34-0.69 | < 0.01 | 41.3% | 0.164 | 238 | 694 | 0.34 | 0.24–0.47 | < 0.01 | 0.0% | 0.994 | 53 | 30 | 0.42 | 0.22-0.82 | 0.01 | - | - |
|  | CIR | 46 | 37 | 0.30 | 0.12-0.74 | 0.01 | - | - | 211 | 683 | 0.31 | 0.20-0.46 | < 0.01 | 0.0% | 0.827 | - | - | - | - | - | - | - |
|  | CR | 235/337, 69.7% | 241/363, 66.39% | 0.88 | 0.68-1.12 | 0.32 | 83.1% | 0.001 | - | - | - | - | - | - | - | 35/53, 66.0% | 9/30, 30.0% | 0.45 | 0.25-0.81 | 0.01 | - | - |
| Midostaurin | OS | 458 | 447 | 0.74 | 0.62–0.88 | < 0.01 | 0.0% | 0.428 | 30 | 30 | 0.58 | 0.19-1.78 | 0.34 | - | - | - | - | - | - | - | - | - |
|  | EFS | 644 | 772 | 0.67 | 0.50-0.90 | 0.01 | 80.7% | 0.023 | - | - | - | - | - | - | - | - | - | - | - | - | - | - |
|  | RFS | 212 | 191 | 0.68 | 0.51-0.91 | 0.01 | - | - | - | - | - | - | - | - | - | - | - | - | - | - | - | - |
|  | CIR | 212 | 191 | 0.67 | 0.40-1.13 | 0.13 | - | - | 30 | 30 | 0.46 | 0.12-1.81 | 0.26 | - | - | - | - | - | - | - | - | - |
|  | CR | 271/394, 68.8% | 236/392, 60.2% | 0.85 | 0.71-1.02 | 0.08 | 34.2% | 0.218 | - | - | - | - | - | - | - | - | - | - | - | - | - | - |
| Gilteritinib | OS | - | - | - | - | - | - | - | - | - | - | - | - | - | - | 247 | 124 | 0.64 | 0.49-0.83 | < 0.01 | - | - |
|  | EFS | - | - | - | - | - | - | - | - | - | - | - | - | - | - | 247 | 124 | 0.79 | 0.58-1.09 | 0.14 | - | - |
|  | RFS | - | - | - | - | - | - | - | - | - | - | - | - | - | - | 52 | 13 | 0.21 | 0.02-1.91 | 0.18 | - | - |
|  | CR | - | - | - | - | - | - | - | - | - | - | - | - | - | - | 84/247, 34.0% | 19/124, 15.3% | 0.45 | 0.29-0.71 | < 0.01 | - | - |
| Quizartinib | OS | - | - | - | - | - | - | - | - | - | - | - | - | - | - | 245 | 122 | 0.76 | 0.58-0.98 | 0.04 | - | - |
|  | EFS | - | - | - | - | - | - | - | - | - | - | - | - | - | - | 245 | 122 | 0.90 | 0.70-1.16 | 0.41 | - | - |
|  | CR | - | - | - | - | - | - | - | - | - | - | - | - | - | - | 118/245, 48.2% | 33/122, 27.0% | 0.56 | 0.41-0.77 | < 0.01 | - | - |
| Lestaurtinib | OS | 300 | 200 | 0.90 | 0.70-1.15 | 0.41 | 0.0% | 0.830 | - | - | - | - | - | - | - | 112 | 112 | 1.04 | 0.78-1.39 | 0.79 | - | - |
|  | RFS | 276 | 188 | 0.87 | 0.69-1.11 | 0.25 | 0.0% | 0.753 | - | - | - | - | - | - | - | - | - | - | - | - | - | - |
|  | CIR | 276 | 188 | 0.89 | 0.71-1.11 | 0.31 | 0.0% | 0.340 | - | - | - | - | - | - | - | - | - | - | - | - | - | - |
|  | CR | 277/300, 92.3% | 188/200, 94.0% | 1.36 | 0.67-2.76 | 0.39 | 0.0% | 0.654 | - | - | - | - | - | - | - | 29/112, 25.9% | 23/112, 20.5% | 0.79 | 0.49-1.28 | 0.34 | - | - |

Abbreviations: FLT3, FMS-like tyrosine kinase 3; FLT3i, FLT3 inhibitor; AML, acute myeloid leukemia; allo-HSCT, allogeneic hematopoietic stem cell transplant; rrAML, refractory and relapsed acute myeloid leukemia; RR, relative risk; HR, hazard ratio; 95% CI, 95% confidence interval; OS, overall survival; EFS, event-free survival; RFS, relapse-free survival; CIR, cumulative incidence of relapse; CR, complete remission.

**Supplementary Table 6. Identification of FLT3-ITD allelic ratio in included studies.**

| **Study** | **Year** | **FLT3i** | **Stratifications of FLT3-ITD allelic ratio** |
| --- | --- | --- | --- |
| Ofran | 2020 | Midostaurin | Low: ratio≤0.5;  High: ratio>0.5 |
| Burchert | 2020 | Sorafenib | Low: ratio<0.5;  High: ratio≥0.5 |
| Cortes | 2019 | Quizartinib | Low: 0.03<ratio≤0.25;  Intermediate: 0.25<ratio≤0.5;  High: ratio>0.5 |
| Stone | 2017 | Midostaurin | Low: ratio≤0.7;  High: ratio>0.7 |
| Krapper | 2017 | Lestaurtinib | Low: ratio<0.25;  Intermediate: 0.25≤ratio≤0.5;  High: ratio>0.5 |

**Abbreviations:** FLT3, FMS-like tyrosine kinase 3; FLT3i, FLT3 inhibitor; FLT3-ITD, FMS-like tyrosine kinase 3-internal tandem duplication.

**Supplementary Table 7. Pooled toxicity.**

| Study | Year | FLT3i | Stage | Grade | Early death | | | | | | Thrombocytopenia | | | | | | Neutropenia | | | | | |
| --- | --- | --- | --- | --- | --- | --- | --- | --- | --- | --- | --- | --- | --- | --- | --- | --- | --- | --- | --- | --- | --- | --- |
|  |  |  |  |  | FLT3i | Control | RR | Lower 95% CI | Upper 95% CI | P-value | FLT3i | Control | RR | Lower 95% CI | Upper 95% CI | P-value | FLT3i | Control | RR | Lower 95% CI | Upper 95% CI | P-value |
| Xuan | 2020 | Sorafenib | Maintenance therapy post allo-HSCT | All grade |  |  |  |  |  |  | 13/100 | 6/102 | 2.210 | 0.874 | 5.586 | 0.094 | 9/100 | 4/102 | 2.295 | 0.730 | 7.212 | 0.155 |
| Xuan | 2020 | Sorafenib | Maintenance therapy post allo-HSCT | Grade ≥3 |  |  |  |  |  |  | 13/100 | 6/102 | 2.210 | 0.874 | 5.586 | 0.094 | 9/100 | 4/102 | 2.295 | 0.730 | 7.212 | 0.155 |
| Burchert | 2020 | Sorafenib | Maintenance therapy post allo-HSCT | Grade ≥3 |  |  |  |  |  |  | 2/42 | 1/39 | 1.857 | 0.175 | 19.680 | 0.607 | 1/42 | 1/39 | 0.929 | 0.060 | 14.342 | 0.958 |
| Sasaki | 2019 | Sorafenib | Induction and consolidation therapy | All grade | 0/79 | 4/104 | 0.146 | 0.008 | 2.670 | 0.194 |  |  |  |  |  |  |  |  |  |  |  |  |
| Zhang | 2017 | Sorafenib | Induction and consolidation therapy | All grade | 1/22 | 5/31 | 0.282 | 0.042 | 1.873 | 0.190 |  |  |  |  |  |  |  |  |  |  |  |  |
| Serve | 2013 | Sorafenib | Induction, consolidation and maintenance therapy after CR | Grade ≥3 | 17/102 | 7/95 | 2.262 | 0.993 | 5.153 | 0.052 |  |  |  |  |  |  | 58/102 | 50/95 | 1.080 | 0.837 | 1.394 | 0.552 |
| Perl | 2019 | Gilteritinib | Salvage therapy for rrAML | All grade |  |  |  |  |  |  | 63/246 | 18/109 | 1.551 | 0.967 | 2.488 | 0.069 | 115/246 | 40/109 | 1.274 | 0.963 | 1.686 | 0.091 |
| Perl | 2019 | Gilteritinib | Salvage therapy for rrAML | Grade ≥3 |  |  |  |  |  |  | 56/246 | 18/109 | 1.379 | 0.852 | 2.230 | 0.191 | 113/246 | 40/109 | 1.252 | 0.945 | 1.659 | 0.118 |
| Cortes | 2019 | Quizartinib | Salvage therapy for rrAML | All grade |  |  |  |  |  |  | 94/241 | 32/94 | 2.938 | 2.051 | 4.207 | 0.000 | 81/241 | 24/94 | 1.316 | 0.893 | 1.941 | 0.165 |
| Cortes | 2019 | Quizartinib | Salvage therapy for rrAML | Grade ≥3 |  |  |  |  |  |  | 85/241 | 32/94 | 2.656 | 1.844 | 3.827 | 0.000 | 76/241 | 23/94 | 1.289 | 0.863 | 1.925 | 0.215 |
| Stone | 2017 | Midostaurin | Induction, consolidation and maintenance therapy after CR | Grade ≥3 |  |  |  |  |  |  | 346/355 | 342/354 | 1.009 | 0.982 | 1.036 | 0.520 | 338/355 | 339/354 | 0.994 | 0.932 | 1.061 | 0.860 |
| Knapper | 2017 | Lestaurtinib | Induction therapy | All grade | 3/88 | 2/87 | 1.500 | 0.260 | 8.630 | 0.700 |  |  |  |  |  |  |  |  |  |  |  |  |
| Knapper | 2017 | Lestaurtinib | Induction therapy | All grade | 2/212 | 0/113 | 4.640 | 0.430 | 49.900 | 0.200 |  |  |  |  |  |  |  |  |  |  |  |  |
| Levis | 2011 | Lestaurtinib | Salvage therapy for rrAML | All grade | 13/111 | 7/109 | 1.824 | 0.669 | 4.969 | 0.240 |  |  |  |  |  |  |  |  |  |  |  |  |
|  |  |  | Summary in random-effects model | All grade | 36/614, 5.86% | 25/539, 4.64% | 1.420 | 0.680 | 2.980 | 0.352 | **170/587, 28.96%** | **56/305, 18.36%** | **2.20** | **1.39** | **3.48** | **0.0008** | **205/587, 34.92%** | **68/305, 22.30%** | **1.32** | **1.05** | **1.65** | **0.016** |
|  |  |  | Heterogeneity, I^2 |  |  |  | 31.9% |  |  |  |  |  | **55.2%** |  |  |  |  |  | **0.0%** |  |  |  |
|  |  |  | Heterogeneity, p-value |  |  |  | 0.197 |  |  |  |  |  | **0.108** |  |  |  |  |  | **0.620** |  |  |  |
|  |  |  | Summary in random-effects model | Grade ≥3 |  |  |  |  |  |  | 502/984, 51.02% | 399/698, 57.16% | 1.63 | 0.94 | 2.84 | 0.083 | 595/1086, 54.79% | 457/793, 57.63% | 1.07 | 0.95 | 1.2 | 0.256252 |
|  |  |  | Heterogeneity, I^2 |  |  |  |  |  |  |  |  |  | 87.20% |  |  |  |  |  | 17.70% |  |  |  |
|  |  |  | Heterogeneity, p-value |  |  |  |  |  |  |  |  |  | 0 |  |  |  |  |  | 0.299 |  |  |  |
| Study | Year | FLT3i | Stage | Grade | Anemia | | | | | | Skin | | | | | | Gastrointestinal | | | | | |
|  |  |  |  |  | FLT3i | Control | RR | Lower 95% CI | Upper 95% CI | P-value | FLT3i | Control | RR | Lower 95% CI | Upper 95% CI | P-value | FLT3i | Control | RR | Lower 95% CI | Upper 95% CI | P-value |
| Xuan | 2020 | Sorafenib | Maintenance therapy post allo-HSCT | All grade |  |  |  |  |  |  | 27/100 | 10/102 | 2.754 | 1.408 | 5.388 | 0.003 | 36/100 | 28/102 | 1.311 | 0.871 | 1.975 | 0.195 |
| Xuan | 2020 | Sorafenib | Maintenance therapy post allo-HSCT | Grade ≥3 |  |  |  |  |  |  | 7/100 | 1/102 | 7.140 | 0.895 | 56.984 | 0.064 | 11/100 | 8/102 | 1.403 | 0.589 | 3.341 | 0.445 |
| Burchert | 2020 | Sorafenib | Maintenance therapy post allo-HSCT | Grade ≥3 |  |  |  |  |  |  | 5/42 | 1/39 | 4.643 | 0.567 | 38.003 | 0.152 | 6/42 | 6/39 | 0.929 | 0.327 | 2.638 | 0.889 |
| Roellig | 2015 | Sorafenib | Induction, consolidation and maintenance therapy after CR | All grade |  |  |  |  |  |  | 83/134 | 62/133 | 1.329 | 1.061 | 1.664 | 0.013 |  |  |  |  |  |  |
| Roellig | 2015 | Sorafenib | Induction, consolidation and maintenance therapy after CR | Grade ≥3 |  |  |  |  |  |  | 9/134 | 4/133 | 4.060 | 1.250 | 15.700 | 0.026 |  |  |  |  |  |  |
| Serve | 2013 | Sorafenib | Induction, consolidation and maintenance therapy after CR | Grade ≥3 |  |  |  |  |  |  | 13/102 | 7/95 | 1.730 | 0.721 | 4.151 | 0.220 |  |  |  |  |  |  |
| Perl | 2019 | Gilteritinib | Salvage therapy for rrAML | All grade | 116/246 | 38/109 | 1.353 | 1.013 | 1.805 | 0.040 | 36/246 | 10/109 | 1.595 | 0.822 | 3.097 | 0.168 |  |  |  |  |  |  |
| Perl | 2019 | Gilteritinib | Salvage therapy for rrAML | Grade ≥3 | 100/246 | 33/109 | 1.343 | 0.973 | 1.854 | 0.073 | 1/246 | 1/109 | 0.443 | 0.028 | 7.019 | 0.564 |  |  |  |  |  |  |
| Cortes | 2019 | Quizartinib | Salvage therapy for rrAML | All grade | 88/241 | 30/94 | 1.144 | 0.815 | 1.606 | 0.436 | 55/241 | 17/94 | 1.262 | 0.774 | 2.058 | 0.351 |  |  |  |  |  |  |
| Cortes | 2019 | Quizartinib | Salvage therapy for rrAML | Grade ≥3 | 72/241 | 27/94 | 1.040 | 0.717 | 1.510 | 0.836 | 6/241 | 0/94 | 5.103 | 0.290 | 89.705 | 0.265 |  |  |  |  |  |  |
| Berger | 2019 | Midostaurin | Induction, consolidation and maintenance therapy after CR | All grade |  |  |  |  |  |  | 6/34 | 8/35 | 0.772 | 0.423 | 1.410 | 0.400 | 16/34 | 8/35 | 2.059 | 1.017 | 4.168 | 0.045 |
| Berger | 2019 | Midostaurin | Induction, consolidation and maintenance therapy after CR | Grade ≥3 |  |  |  |  |  |  | - | - |  |  |  |  | 1/34 | 0/35 | 3.086 | 0.130 | 73.215 | 0.486 |
| Stone | 2017 | Midostaurin | Induction, consolidation and maintenance therapy after CR | Grade ≥3 | 329/355 | 311/354 | 1.055 | 1.005 | 1.107 | 0.030 | 50/355 | 27/354 | 1.847 | 1.174 | 2.906 | 0.008 |  |  |  |  |  |  |
|  |  |  | Summary in random-effects model | All grade | **204/487, 41.89%** | **68/203, 33.50%** | **1.26** | **1.01** | **1.57** | **0.040** | **207/755, 27.42%** | **107/473, 22.62%** | **1.37** | **1.01** | **1.87** | **0.045** | **52/134, 38.8%** | **36/137, 26.28%** | **1.49** | **1** | **2.23** | **0.051** |
|  |  |  | Heterogeneity, I^2 |  |  |  | **0.0%** |  |  |  |  |  | **49.9%** |  |  |  |  |  | **15.10%** |  |  |  |
|  |  |  | Heterogeneity, p-value |  |  |  | **0.460** |  |  |  |  |  | **0.092** |  |  |  |  |  | **0.278** |  |  |  |
|  |  |  | Summary in random-effects model | Grade ≥3 | 501/842, 59.50% | 371/557, 66.60% | 1.07 | 0.99 | 1.15 | 0.077 | **91/1220, 7.46%** | **41/926, 4.43%** | **2.07** | **1.44** | **2.99** | **0.0001** | 18/176, 10.23% | 14/176, 7.95% | 1.23 | 0.64 | 2.37 | 0.535 |
|  |  |  | Heterogeneity, I^2 |  |  |  | 5.50% |  |  |  |  |  | **0.0%** |  |  |  |  |  | 0.0% |  |  |  |
|  |  |  | Heterogeneity, p-value |  |  |  | 0.347 |  |  |  |  |  | **0.543** |  |  |  |  |  | 0.708 |  |  |  |
| Study | Year | FLT3i | Stage | Grade | Vomiting | | | | | | Diarrhea | | | | | | Hepatobiliary or pancreatic | | | | | |
|  |  |  |  |  | FLT3i | Control | RR | Lower 95% CI | Upper 95% CI | P-value | FLT3i | Control | RR | Lower 95% CI | Upper 95% CI | P-value | FLT3i | Control | RR | Lower 95% CI | Upper 95% CI | P-value |
| Xuan | 2020 | Sorafenib | Maintenance therapy post allo-HSCT | All grade |  |  |  |  |  |  |  |  |  |  |  |  | 21/100 | 23/102 | 0.931 | 0.552 | 1.572 | 0.790 |
| Xuan | 2020 | Sorafenib | Maintenance therapy post allo-HSCT | Grade ≥3 |  |  |  |  |  |  |  |  |  |  |  |  | 5/100 | 6/102 | 0.850 | 0.268 | 2.696 | 0.783 |
| Burchert | 2020 | Sorafenib | Maintenance therapy post allo-HSCT | Grade ≥3 |  |  |  |  |  |  |  |  |  |  |  |  | 2/42 | 2/39 | 0.929 | 0.137 | 6.276 | 0.939 |
| Roellig | 2015 | Sorafenib | Induction, consolidation and maintenance therapy after CR | All grade | 46/134 | 63/133 | 0.725 | 0.540 | 0.973 | 0.032 | 93/134 | 86/133 | 1.073 | 0.907 | 1.270 | 0.411 | 16/134 | 13/133 | 1.222 | 0.612 | 2.439 | 0.571 |
| Roellig | 2015 | Sorafenib | Induction, consolidation and maintenance therapy after CR | Grade ≥3 | 2/134 | 1/133 | 2.820 | 0.260 | 61.900 | 0.405 | 15/134 | 5/133 | 7.890 | 2.940 | 25.200 | <0.0001 | 11/134 | 6/133 | 2.750 | 0.990 | 8.000 | 0.052 |
| Serve | 2013 | Sorafenib | Induction, consolidation and maintenance therapy after CR | Grade ≥3 |  |  |  |  |  |  | 14/102 | 6/95 | 2.173 | 0.871 | 5.424 | 0.096 |  |  |  |  |  |  |
| Perl | 2019 | Gilteritinib | Salvage therapy for rrAML | All grade | 53/246 | 15/109 | 1.566 | 0.924 | 2.652 | 0.096 | 81/246 | 32/109 | 1.122 | 0.797 | 1.578 | 0.510 |  |  |  |  |  |  |
| Perl | 2019 | Gilteritinib | Salvage therapy for rrAML | Grade ≥3 | 1/246 | 0/109 | 1.336 | 0.055 | 32.538 | 0.859 | 9/246 | 3/109 | 1.329 | 0.367 | 4.815 | 0.665 |  |  |  |  |  |  |
| Cortes | 2019 | Quizartinib | Salvage therapy for rrAML | All grade | 80/241 | 20/94 | 1.560 | 1.017 | 2.394 | 0.042 | 70/241 | 34/94 | 0.803 | 0.575 | 1.121 | 0.197 |  |  |  |  |  |  |
| Cortes | 2019 | Quizartinib | Salvage therapy for rrAML | Grade ≥3 | 8/241 | 1/94 | 3.120 | 0.396 | 24.608 | 0.280 | 4/241 | 3/94 | 0.520 | 0.119 | 2.280 | 0.386 |  |  |  |  |  |  |
| Berger | 2019 | Midostaurin | Induction, consolidation and maintenance therapy after CR | All grade |  |  |  |  |  |  |  |  |  |  |  |  | 10/34 | 5/35 | 2.059 | 0.785 | 5.400 | 0.142 |
| Berger | 2019 | Midostaurin | Induction, consolidation and maintenance therapy after CR | Grade ≥3 |  |  |  |  |  |  |  |  |  |  |  |  | 2/34 | 1/35 | 2.059 | 0.196 | 21.666 | 0.548 |
| Stone | 2017 | Midostaurin | Induction, consolidation and maintenance therapy after CR | Grade ≥3 |  |  |  |  |  |  | 56/355 | 54/354 | 1.034 | 0.537 | 1.990 | 0.920 |  |  |  |  |  |  |
|  |  |  | Summary in random-effects model | All grade | 179/621, 28.82% | 98/336, 29.17% | 1.180 | 0.670 | 2.080 | 0.567 | 244/621, 39.29% | 152/336, 45.24% | 1.020 | 0.860 | 1.210 | 0.820 | 47/268, 17.54% | 41/270, 15.19% | 1.150 | 0.780 | 1.700 | 0.482 |
|  |  |  | Heterogeneity, I^2 |  |  |  | 82.6% |  |  |  |  |  | 23.2% |  |  |  |  |  | 2.8% |  |  |  |
|  |  |  | Heterogeneity, p-value |  |  |  | 0.003 |  |  |  |  |  | 0.272 |  |  |  |  |  | 0.357 |  |  |  |
|  |  |  | Summary in random-effects model | Grade ≥3 | 11/621, 1.77% | 2/336, 0.60% | 2.54 | 0.59 | 10.96 | 0.211 | 98/1078, 9.09% | 71/785, 9.04% | 1.720 | 0.750 | 3.920 | 0.199 | 20/310, 6.45% | 15/309, 4.85% | 1.540 | 0.770 | 3.060 | 0.220 |
|  |  |  | Heterogeneity, I^2 |  |  |  | 0.0% |  |  |  |  |  | 69.0% |  |  |  |  |  | 0.0% |  |  |  |
|  |  |  | Heterogeneity, p-value |  |  |  | 0.905 |  |  |  |  |  | 0.012 |  |  |  |  |  | 0.470 |  |  |  |
| Study | Year | FLT3i | Stage | Grade | Alanine aminotransferase increased | | | | | | Aspartate aminotransferase increased | | | | | | Cardiac | | | | | |
|  |  |  |  |  | FLT3i | Control | RR | Lower 95% CI | Upper 95% CI | P-value | FLT3i | Control | RR | Lower 95% CI | Upper 95% CI | P-value | FLT3i | Control | RR | Lower 95% CI | Upper 95% CI | P-value |
| Xuan | 2020 | Sorafenib | Maintenance therapy post allo-HSCT | All grade |  |  |  |  |  |  |  |  |  |  |  |  | 14/100 | 13/102 | 1.099 | 0.544 | 2.218 | 0.793 |
| Xuan | 2020 | Sorafenib | Maintenance therapy post allo-HSCT | Grade ≥3 |  |  |  |  |  |  |  |  |  |  |  |  | 0/100 | 1/102 | 0.340 | 0.014 | 8.247 | 0.507 |
| Roellig | 2015 | Sorafenib | Induction, consolidation and maintenance therapy after CR | All grade |  |  |  |  |  |  |  |  |  |  |  |  | 40/134 | 24/133 | 1.654 | 1.059 | 2.583 | 0.027 |
| Roellig | 2015 | Sorafenib | Induction, consolidation and maintenance therapy after CR | Grade ≥3 |  |  |  |  |  |  |  |  |  |  |  |  | 10/134 | 4/133 | 3.460 | 1.150 | 11.800 | 0.033 |
| Serve | 2013 | Sorafenib | Induction, consolidation and maintenance therapy after CR | Grade ≥3 |  |  |  |  |  |  |  |  |  |  |  |  | 6/102 | 2/95 | 2.794 | 0.578 | 13.507 | 0.201 |
| Perl | 2019 | Gilteritinib | Salvage therapy for rrAML | All grade | 103/246 | 10/109 | 4.5638 | 2.4828 | 8.389 | 0 | 99/246 | 13/109 | 3.374 | 1.981 | 5.746 | 0.000 |  |  |  |  |  |  |
| Perl | 2019 | Gilteritinib | Salvage therapy for rrAML | Grade ≥3 | 34/246 | 5/109 | 3.013 | 1.2113 | 7.4948 | 0.0177 | 36/246 | 2/109 | 7.976 | 1.955 | 32.534 | 0.004 |  |  |  |  |  |  |
| Cortes | 2019 | Quizartinib | Salvage therapy for rrAML | All grade | 32/241 | 4/94 | 3.1203 | 1.1345 | 8.582 | 0.0275 |  |  |  |  |  |  | 64/241 | - | - | - | - | - |
| Cortes | 2019 | Quizartinib | Salvage therapy for rrAML | Grade ≥3 | 9/241 | 2/94 | 1.7552 | 0.3864 | 7.9733 | 0.4663 |  |  |  |  |  |  | 10/241 | - | - | - | - | - |
| Stone | 2017 | Midostaurin | Induction, consolidation and maintenance therapy after CR | Grade ≥3 | 45/355 | 33/354 | 1.3598 | 0.858737 | 2.153227 | 0.19 |  |  |  |  |  |  |  |  |  |  |  |  |
|  |  |  | Summary in random-effects model | All grade | **135/487, 27.72%** | **14/203, 6.90%** | **4.13** | **2.45** | **6.95** | **9.70E-08** | 99/246,40.24% | 13/109, 11.93% | 3.374 | 1.981 | 5.746 | 0.000 | **54/234, 23.08%** | **37/235, 15.74%** | **1.47** | **1.01** | **2.14** | **0.044** |
|  |  |  | Heterogeneity, I^2 |  |  |  | **0.0%** |  |  |  |  |  | - |  |  |  |  |  | **0.0%** |  |  |  |
|  |  |  | Heterogeneity, p-value |  |  |  | **0.528** |  |  |  |  |  | - |  |  |  |  |  | **0.336** |  |  |  |
|  |  |  | Summary in random-effects model | Grade ≥3 | **88/842, 10.45%** | **44/557, 7.90%** | **1.68** | **1.04** | **2.72** | **0.034** | 36/246, 14.63% | 2/109, 1.83% | 7.976 | 1.955 | 32.534 | 0.004 | **16/336, 4.76%** | **7/330, 2.12%** | **2.68** | **1.09** | **6.59** | **0.032** |
|  |  |  | Heterogeneity, I^2 |  |  |  | **14.80%** |  |  |  |  |  | - |  |  |  |  |  | **0.0%** |  |  |  |
|  |  |  | Heterogeneity, p-value |  |  |  | **0.309** |  |  |  |  |  | - |  |  |  |  |  | **0.407** |  |  |  |
| Study | Year | FLT3i | Stage | Grade | Infections | | | | | | Acute GVHD | | | | | | Chronic GVHD | | | | | |
|  |  |  |  |  | FLT3i | Control | RR | Lower 95% CI | Upper 95% CI | P-value | FLT3i | Control | RR | Lower 95% CI | Upper 95% CI | P-value | FLT3i | Control | RR | Lower 95% CI | Upper 95% CI | P-value |
| Xuan | 2020 | Sorafenib | Maintenance therapy post allo-HSCT | All grade | 33/100 | 33/102 | 1.020 | 0.687 | 1.515 | 0.922 | 31/100 | 27/102 | 1.171 | 0.757 | 1.811 | 1.811 | 23/100 | 22/102 | 1.066 | 0.637 | 1.785 | 0.807 |
| Xuan | 2020 | Sorafenib | Maintenance therapy post allo-HSCT | Grade ≥3 | 25/100 | 24/102 | 1.063 | 0.653 | 1.730 | 0.807 | 23/100 | 21/102 | 1.117 | 0.662 | 1.885 | 0.678 | 18/100 | 17/102 | 1.080 | 0.591 | 1.973 | 0.802 |
| Burchert | 2020 | Sorafenib | Maintenance therapy post allo-HSCT | Grade ≥3 | 11/42 | 9/39 | 1.135 | 0.528 | 2.440 | 0.746 | 10/42 | 7/39 | 1.327 | 0.560 | 3.141 | 0.521 | 26/42 | 18/39 | 1.341 | 0.887 | 2.029 | 0.164 |
| Bazarbachi | 2019 | Sorafenib | Maintenance therapy post allo-HSCT | All grade |  |  |  |  |  |  |  |  |  |  |  |  | - | - | 1.840 | 0.960 | 3.530 | 0.070 |
| Bazarbachi | 2019 | Sorafenib | Salvage therapy after relapse post allo-HSCT | All grade |  |  |  |  |  |  | 9/34 | 32/118 | 0.976 | 0.444 | 2.146 | 0.952 | 4/34 | 18/118 | 0.771 | 0.320 | 1.860 | 0.563 |
| Chappell | 2019 | Sorafenib | Maintenance therapy post allo-HSCT | All grade | 1/29 | 1/55 | 1.897 | 0.123 | 29.227 | 0.647 |  |  |  |  |  |  |  |  |  |  |  |  |
| Xuan | 2019 | Sorafenib | Salvage therapy after relapse post allo-HSCT | All grade |  |  |  |  |  |  | 20/53 | 7/30 | 1.617 | 0.775 | 3.373 | 0.200 | 16/53 | 6/30 | 1.509 | 0.662 | 3.443 | 0.328 |
| Brunner | 2016 | Sorafenib | Maintenance therapy post allo-HSCT |  |  |  |  |  |  |  | 7/26 | 6/43 | 1.930 | 0.727 | 5.119 | 0.187 |  |  |  |  |  |  |
| Roellig | 2015 | Sorafenib | Induction, consolidation and maintenance therapy after CR | All grade | 65/134 | 74/133 | 0.872 | 0.692 | 1.099 | 0.245 |  |  |  |  |  |  |  |  |  |  |  |  |
| Roellig | 2015 | Sorafenib | Induction, consolidation and maintenance therapy after CR | Grade ≥3 | 48/134 | 55/133 | 1.100 | 0.710 | 1.720 | 0.666 |  |  |  |  |  |  |  |  |  |  |  |  |
| Serve | 2013 | Sorafenib | Induction, consolidation and maintenance therapy after CR | Grade ≥3 | 39/102 | 30/95 | 1.211 | 0.824 | 1.780 | 0.331 |  |  |  |  |  |  |  |  |  |  |  |  |
| Perl | 2019 | Gilteritinib | Salvage therapy for rrAML | All grade | 43/246 | 8/109 | 2.382 | 1.159 | 4.894 | 0.018 |  |  |  |  |  |  |  |  |  |  |  |  |
| Perl | 2019 | Gilteritinib | Salvage therapy for rrAML | Grade ≥3 | 29/246 | 5/109 | 2.570 | 1.022 | 6.461 | 0.045 |  |  |  |  |  |  |  |  |  |  |  |  |
| Cortes | 2019 | Quizartinib | Salvage therapy for rrAML | All grade | 38/241 | 10/94 | 1.482 | 0.770 | 2.852 | 0.239 |  |  |  |  |  |  |  |  |  |  |  |  |
| Cortes | 2019 | Quizartinib | Salvage therapy for rrAML | Grade ≥3 | 29/241 | 8/94 | 1.414 | 0.671 | 2.980 | 0.363 |  |  |  |  |  |  |  |  |  |  |  |  |
| Stone | 2017 | Midostaurin | Induction, consolidation and maintenance therapy after CR | Grade ≥3 | 186/355 | 178/354 | 1.042 | 0.903 | 1.203 | 0.574 |  |  |  |  |  |  |  |  |  |  |  |  |
|  |  |  | Summary in random-effects model | All grade | 180/930, 19.35% | 126/493, 25.56% | 1.180 | 0.830 | 1.690 | 0.362 | 67/213, 31.46% | 72/293, 24.57% | 1.270 | 0.930 | 1.760 | 0.142 | 43/187, 22.99% | 36/250, 14.4% | 1.25 | 0.89 | 1.75 | 0.196 |
|  |  |  | Heterogeneity, I^2 |  |  |  | 53.1% |  |  |  |  |  | 0.0% |  |  |  |  |  | 2.60% |  |  |  |
|  |  |  | Heterogeneity, p-value |  |  |  | 0.074 |  |  |  |  |  | 0.641 |  |  |  |  |  | 0.379 |  |  |  |
|  |  |  | Summary in random-effects model | Grade ≥3 | 367/1220, 30.08% | 309/926, 33.37% | 1.090 | 0.970 | 1.230 | 0.155 | 33/142, 23.24% | 28/141, 19.86% | 1.170 | 0.750 | 1.830 | 0.490 | 44/142, 30.99% | 35/141, 24.82% | 1.25 | 0.89 | 1.76 | 0.200 |
|  |  |  | Heterogeneity, I^2 |  |  |  | 0.0% |  |  |  |  |  | 0.0% |  |  |  |  |  | 0.0% |  |  |  |
|  |  |  | Heterogeneity, p-value |  |  |  | 0.612 |  |  |  |  |  | 0.738 |  |  |  |  |  | 0.562 |  |  |  |
| Study | Year | FLT3i | Stage | Grade | Pyrexia | | | | | | Cough | | | | | | Dyspnea | | | | | |
|  |  |  |  |  | FLT3i | Control | RR | Lower 95% CI | Upper 95% CI | P-value | FLT3i | Control | RR | Lower 95% CI | Upper 95% CI | P-value | FLT3i | Control | RR | Lower 95% CI | Upper 95% CI | P-value |
| Roellig | 2015 | Sorafenib | Induction, consolidation and maintenance therapy after CR | All grade | 127/134 | 120/133 | 1.050 | 0.981 | 1.125 | 0.160 |  |  |  |  |  |  |  |  |  |  |  |  |
| Roellig | 2015 | Sorafenib | Induction, consolidation and maintenance therapy after CR | Grade ≥3 | 73/134 | 71/133 | 1.540 | 1.040 | 2.280 | 0.030 |  |  |  |  |  |  |  |  |  |  |  |  |
| Serve | 2013 | Sorafenib | Induction, consolidation and maintenance therapy after CR | Grade ≥3 |  |  |  |  |  |  |  |  |  |  |  |  | 1/95 | 3/102 | 0.311 | 0.033 | 2.933 | 0.307 |
| Perl | 2019 | Gilteritinib | Salvage therapy for rrAML | All grade | 105/246 | 32/109 | 1.454 | 1.050 | 2.013 | 0.024 | 72/246 | 11/109 | 2.900 | 1.603 | 5.248 | 0.000 | 58/246 | 7/109 | 3.671 | 1.732 | 7.781 | 0.001 |
| Perl | 2019 | Gilteritinib | Salvage therapy for rrAML | Grade ≥3 | 8/246 | 4/109 | 0.886 | 0.273 | 2.881 | 0.841 | 1/246 | 0/109 | 1.336 | 0.055 | 32.538 | 0.859 | 10/246 | 3/109 | 1.477 | 0.415 | 5.261 | 0.547 |
| Cortes | 2019 | Quizartinib | Salvage therapy for rrAML | All grade | 92/241 | 42/94 | 0.854 | 0.648 | 1.126 | 0.265 | 56/241 | 13/94 | 1.680 | 0.965 | 2.925 | 0.067 | 49/241 | 8/94 | 2.389 | 1.177 | 4.851 | 0.016 |
| Cortes | 2019 | Quizartinib | Salvage therapy for rrAML | Grade ≥3 | 6/241 | 4/94 | 0.585 | 0.169 | 2.027 | 0.398 | 1/241 | 0/94 | 1.178 | 0.048 | 28.656 | 0.920 | 12/241 | 5/94 | 0.936 | 0.339 | 2.585 | 0.899 |
|  |  |  | Summary in random-effects model | All grade | 324/621, 52.17% | 194/336, 57.74% | 1.070 | 0.860 | 1.330 | 0.543 | **128/487, 26.28%** | **24/203, 11.82%** | **2.180** | **1.280** | **3.730** | **0.004** | **107/487, 21.97%** | **15/203, 7.39%** | **2.920** | **1.750** | **4.900** | **0.00005** |
|  |  |  | Heterogeneity, I^2 |  |  |  | 66.6% |  |  |  |  |  | **42.4%** |  |  |  |  |  | **0.00%** |  |  |  |
|  |  |  | Heterogeneity, p-value |  |  |  | 0.050 |  |  |  |  |  | **0.188** |  |  |  |  |  | **0.415** |  |  |  |
|  |  |  | Summary in random-effects model | Grade ≥3 | 87/621, 14.01% | 79/336, 23.51% | 1.200 | 0.700 | 2.050 | 0.506 | 2/487, 0.41% | 0/203, 0.00% | 1.250 | 0.130 | 12.000 | 0.847 | 23/582, 3.95% | 11/305, 3.61% | 0.970 | 0.460 | 2.050 | 0.936 |
|  |  |  | Heterogeneity, I^2 |  |  |  | 24.9% |  |  |  |  |  | 0.00% |  |  |  |  |  | 0.00% |  |  |  |
|  |  |  | Heterogeneity, p-value |  |  |  | 0.264 |  |  |  |  |  | 0.956 |  |  |  |  |  | 0.493 |  |  |  |
| Study | Year | FLT3i | Stage | Grade | Pain | | | | | | Hypokalemia | | | | | | Fatigue | | | | | |
|  |  |  |  |  | FLT3i | Control | RR | Lower 95% CI | Upper 95% CI | P-value | FLT3i | Control | RR | Lower 95% CI | Upper 95% CI | P-value | FLT3i | Control | RR | Lower 95% CI | Upper 95% CI | P-value |
| Roellig | 2015 | Sorafenib | Induction, consolidation and maintenance therapy after CR | All grade | 92/134 | 95/133 | 0.961 | 0.822 | 1.125 | 0.621 |  |  |  |  |  |  | 17/134 | 26/133 | 0.649 | 0.370 | 1.139 | 0.132 |
| Roellig | 2015 | Sorafenib | Induction, consolidation and maintenance therapy after CR | Grade ≥3 | 15/134 | 13/133 | 2.090 | 0.990 | 4.440 | 0.055 |  |  |  |  |  |  | 2/134 | 2/133 | 1.440 | 0.170 | 12.500 | 0.719 |
| Serve | 2013 | Sorafenib | Induction, consolidation and maintenance therapy after CR | Grade ≥3 | 3/102 | 1/95 | 2.794 | 0.296 | 26.400 | 0.370 |  |  |  |  |  |  |  |  |  |  |  |  |
| Perl | 2019 | Gilteritinib | Salvage therapy for rrAML | All grade |  |  |  |  |  |  | 103/246 | 47/109 | 0.971 | 0.748 | 1.261 | 0.825 | 80/246 | 17/109 | 2.085 | 1.300 | 3.344 | 0.002 |
| Perl | 2019 | Gilteritinib | Salvage therapy for rrAML | Grade ≥3 |  |  |  |  |  |  | 32/246 | 13/109 | 1.091 | 0.596 | 1.995 | 0.778 | 10/246 | 3/109 | 1.477 | 0.415 | 5.261 | 0.547 |
| Cortes | 2019 | Quizartinib | Salvage therapy for rrAML | All grade | 54/241 | 16/94 | 1.316 | 0.795 | 2.180 | 0.286 | 78/241 | 26/94 | 1.170 | 0.805 | 1.702 | 0.411 | 95/241 | 27/94 | 1.372 | 0.962 | 1.957 | 0.080 |
| Cortes | 2019 | Quizartinib | Salvage therapy for rrAML | Grade ≥3 | 5/241 | 1/94 | 1.950 | 0.231 | 16.473 | 0.540 | 28/241 | 8/94 | 1.365 | 0.646 | 2.886 | 0.415 | 19/241 | 1/94 | 7.411 | 1.006 | 54.579 | 0.049 |
| Stone | 2017 | Midostaurin | Induction, consolidation and maintenance therapy after CR | Grade ≥3 | 47/355 | 44/354 | 1.065 | 0.618 | 1.835 | 0.820 | 49/355 | 60/354 | 0.814 | 0.575 | 1.153 | 0.247 | 32/355 | 37/354 | 0.862 | 0.543 | 1.369 | 0.530 |
|  |  |  | Summary in random-effects model | All grade | 146/375, 38.93% | 111/227, 48.90% | 1.02 | 0.8 | 1.31 | 0.875 | 181/487, 37.17% | 73/203, 35.96% | 1.030 | 0.830 | 1.280 | 0.789 | 192/621, 30.92% | 70/336, 20.83% | 1.260 | 0.700 | 2.250 | 0.438 |
|  |  |  | Heterogeneity, I^2 |  |  |  | 26.50% |  |  |  |  |  | 0.00% |  |  |  |  |  | 79.5% |  |  |  |
|  |  |  | Heterogeneity, p-value |  |  |  | 0.243 |  |  |  |  |  | 0.423 |  |  |  |  |  | 0.008 |  |  |  |
|  |  |  | Summary in random-effects model | Grade ≥3 | 70/832, 8.41% | 59/676, 8.73% | 1.4 | 0.92 | 2.14 | 0.118 | 109/842, 12.95% | 81/557, 14.54% | 0.930 | 0.700 | 1.230 | 0.614 | 63/976, 6.45% | 43/690, 6.23% | 1.350 | 0.620 | 2.920 | 0.448 |
|  |  |  | Heterogeneity, I^2 |  |  |  | 0.00% |  |  |  |  |  | 0.00% |  |  |  |  |  | 36.5% |  |  |  |
|  |  |  | Heterogeneity, p-value |  |  |  | 0.471 |  |  |  |  |  | 0.398 |  |  |  |  |  | 0.193 |  |  |  |

Abbreviations: FLT3i, FMS-like tyrosine kinase 3 inhibitor; allo-HSCT, allogeneic hematopoietic stem cell transplantation; CR, complete remission; AML, acute myeloid leukemia; RR, relative risk; 95% CI, 95% confidence intervals; rrAML, refractory or relapsed AML; GVHD, graft-versus-host disease.

**Supplementary Table 8.** **Publication bias based on Egger’s and Begg’s tests.**

| **Treatment stage** | **Endpoints** | **Total number of studies** | **P-value of Egger’s test** | **P-value of Begg’s test** |
| --- | --- | --- | --- | --- |
| FLT3i in induction regimen of newly diagnosed AML | CR | 9 | 0.970 | 0.917 |
|  | OS | 10 | 0.178 | 0.210 |
|  | EFS | 5 | 0.149 | 0.462 |
|  | RFS | 7 | 0.038 | 0.016 |
|  | CIR | 5 | 0.103 | 0.221 |
| FLT3i in maintenance regimen post allo-HSCT | OS | 8 | 0.190 | 0.536 |
|  | RFS | 6 | 0.390 | 0.260 |
|  | CIR | 6 | 0.585 | 0.707 |
| allo-HSCT in FLT3 (+) AML | OS | 14 | 0.037 | 0.029 |
|  | EFS | 2 | - | 1.000 |
|  | RFS | 9 | 0.030 | 0.029 |
|  | CIR | 3 | 0.965 | 1.000 |
| FLT3i in salvage regimen of rrAML | CR | 6 | 0.193 | 0.133 |
|  | OS | 7 | 0.072 | 0.035 |
|  | EFS | 2 | - | 1.000 |
|  | RFS | 2 | - | 1.000 |

Abbreviations: FLT3, FMS-like tyrosine kinase 3; FLT3i, FLT3 inhibitor; allo-HSCT, allogeneic hematopoietic stem cell transplant; rrAML, refractory and relapsed acute myeloid leukemia; CR, complete remission; OS, overall survival; EFS, event-free survival; RFS, relapse-free survival; CIR, cumulative incidence of relapse.

**Supplementary Table 9.** **Pooled risk ratios of complete remission rate in network meta-analysis.**

|  | **Chemotherapy** | **Gilteritinib** | **Lestaurtinib** | **Midostaurin** | **Quizartinib** | **Sorafenib** |
| --- | --- | --- | --- | --- | --- | --- |
| **Chemotherapy** | **Chemotherapy** | 0.45 (0.2, 1.06) | 0.99 (0.57, 1.9) | 0.89 (0.41, 1.91) | 0.56 (0.26, 1.25) | 1.08 (0.63, 1.86) |
| **Gilteritinib** | 2.21 (0.95, 5.11) | **Gilteritinib** | 2.19 (0.8, 6.46) | 1.97 (0.64, 6.13) | 1.24 (0.39, 3.95) | 2.38 (0.87, 6.44) |
| **Lestaurtinib** | 1.01 (0.53, 1.77) | 0.46 (0.15, 1.24) | **Lestaurtinib** | 0.91 (0.32, 2.18) | 0.57 (0.2, 1.44) | 1.09 (0.46, 2.32) |
| **Midostaurin** | 1.12 (0.52, 2.44) | 0.51 (0.16, 1.57) | 1.1 (0.46, 3.16) | **Midostaurin** | 0.63 (0.21, 1.94) | 1.21 (0.48, 3.1) |
| **Quizartinib** | 1.78 (0.8, 3.9) | 0.81 (0.25, 2.57) | 1.75 (0.69, 4.96) | 1.59 (0.51, 4.77) | **Quizartinib** | 1.92 (0.73, 4.98) |
| **Sorafenib** | 0.93 (0.54, 1.59) | 0.42 (0.16, 1.15) | 0.91 (0.43, 2.2) | 0.83 (0.32, 2.08) | 0.52 (0.2, 1.36) | **Sorafenib** |

**Supplementary Table 10.** **Pooled hazard ratios of overall survival in network meta-analysis.**

|  | **Chemotherapy** | **Gilteritinib** | **Lestaurtinib** | **Midostaurin** | **Quizartinib** | **Standard of care** | **Sorafenib** |
| --- | --- | --- | --- | --- | --- | --- | --- |
| **Chemotherapy** | **Chemotherapy** | 0.64 (0.39, 1.03) | 0.92 (0.69, 1.24) | 0.8 (0.53, 1.24) | 0.76 (0.47, 1.23) | 1.67 (0.86, 3.14) | 0.82 (0.5, 1.28) |
| **Gilteritinib** | 1.57 (0.97, 2.55) | **Gilteritinib** | 1.45 (0.82, 2.55) | 1.25 (0.67, 2.45) | 1.19 (0.6, 2.36) | 2.63 (1.14, 5.75) | 1.28 (0.64, 2.48) |
| **Lestaurtinib** | 1.08 (0.81, 1.45) | 0.69 (0.39, 1.22) | **Lestaurtinib** | 0.86 (0.53, 1.47) | 0.82 (0.47, 1.45) | 1.81 (0.87, 3.64) | 0.88 (0.5, 1.52) |
| **Midostaurin** | 1.26 (0.8, 1.88) | 0.8 (0.41, 1.49) | 1.16 (0.68, 1.88) | **Midostaurin** | 0.95 (0.5, 1.76) | 2.1 (1.02, 4.1) | 1.03 (0.55, 1.78) |
| **Quizartinib** | 1.32 (0.81, 2.12) | 0.84 (0.42, 1.66) | 1.22 (0.69, 2.14) | 1.05 (0.57, 2.01) | **Quizartinib** | 2.2 (0.97, 4.78) | 1.08 (0.55, 2.06) |
| **Standard of care** | 0.6 (0.32, 1.16) | 0.38 (0.17, 0.88) | 0.55 (0.27, 1.15) | 0.48 (0.24, 0.98) | 0.45 (0.21, 1.03) | **Standard of care** | 0.49 (0.29, 0.81) |
| **Sorafenib** | 1.23 (0.78, 1.99) | 0.78 (0.4, 1.56) | 1.13 (0.66, 1.99) | 0.97 (0.56, 1.82) | 0.93 (0.49, 1.83) | 2.05 (1.23, 3.41) | **Sorafenib** |

**Supplementary Table 11.** **Pooled hazard ratios of event-free survival in network meta-analysis.**

|  | **Chemotherapy** | **Gilteritinib** | **Quizartinib** | **Sorafenib** |
| --- | --- | --- | --- | --- |
| **Chemotherapy** | **Chemotherapy** | 0.79 (0.4, 1.57) | 0.9 (0.47, 1.73) | 0.93 (0.54, 1.4) |
| **Gilteritinib** | 1.26 (0.64, 2.47) | **Gilteritinib** | 1.14 (0.44, 2.92) | 1.17 (0.48, 2.51) |
| **Quizartinib** | 1.11 (0.58, 2.13) | 0.88 (0.34, 2.26) | **Quizartinib** | 1.03 (0.43, 2.16) |
| **Sorafenib** | 1.08 (0.72, 1.85) | 0.86 (0.4, 2.09) | 0.97 (0.46, 2.32) | **Sorafenib** |

**Supplementary Table 12.** **Pooled hazard ratios of relapse-free survival in network meta-analysis.**

|  | **Chemotherapy** | **Gilteritinib** | **Lestaurtinib** | **Midostaurin** | **Srandard of care** | **Sorafenib** |
| --- | --- | --- | --- | --- | --- | --- |
| **Chemotherapy** | **Chemotherapy** | 0.2 (0.02, 2.03) | 0.88 (0.5, 1.58) | 0.68 (0.33, 1.39) | 1.41 (0.56, 3.64) | 0.49 (0.22, 1.11) |
| **Gilteritinib** | 5.02 (0.49, 53.8) | **Gilteritinib** | 4.45 (0.4, 51.31) | 3.41 (0.31, 39.98) | 7.05 (0.58, 92.49) | 2.45 (0.21, 30.52) |
| **Lestaurtinib** | 1.14 (0.63, 2.01) | 0.22 (0.02, 2.5) | **Lestaurtinib** | 0.77 (0.31, 1.92) | 1.6 (0.54, 4.75) | 0.56 (0.21, 1.49) |
| **Midostaurin** | 1.48 (0.72, 3.06) | 0.29 (0.03, 3.26) | 1.3 (0.52, 3.26) | **Midostaurin** | 2.08 (0.77, 5.67) | 0.73 (0.29, 1.87) |
| **Srandard of care** | 0.71 (0.27, 1.78) | 0.14 (0.01, 1.73) | 0.63 (0.21, 1.85) | 0.48 (0.18, 1.29) | **Srandard of care** | 0.35 (0.18, 0.67) |
| **Sorafenib** | 2.04 (0.9, 4.6) | 0.41 (0.03, 4.72) | 1.8 (0.67, 4.75) | 1.38 (0.53, 3.5) | 2.88 (1.5, 5.46) | **Sorafenib** |

**Supplementary Figures**

**Supplementary Figure 1. Risk of bias graph in quality assessment for randomized controlled trials.**


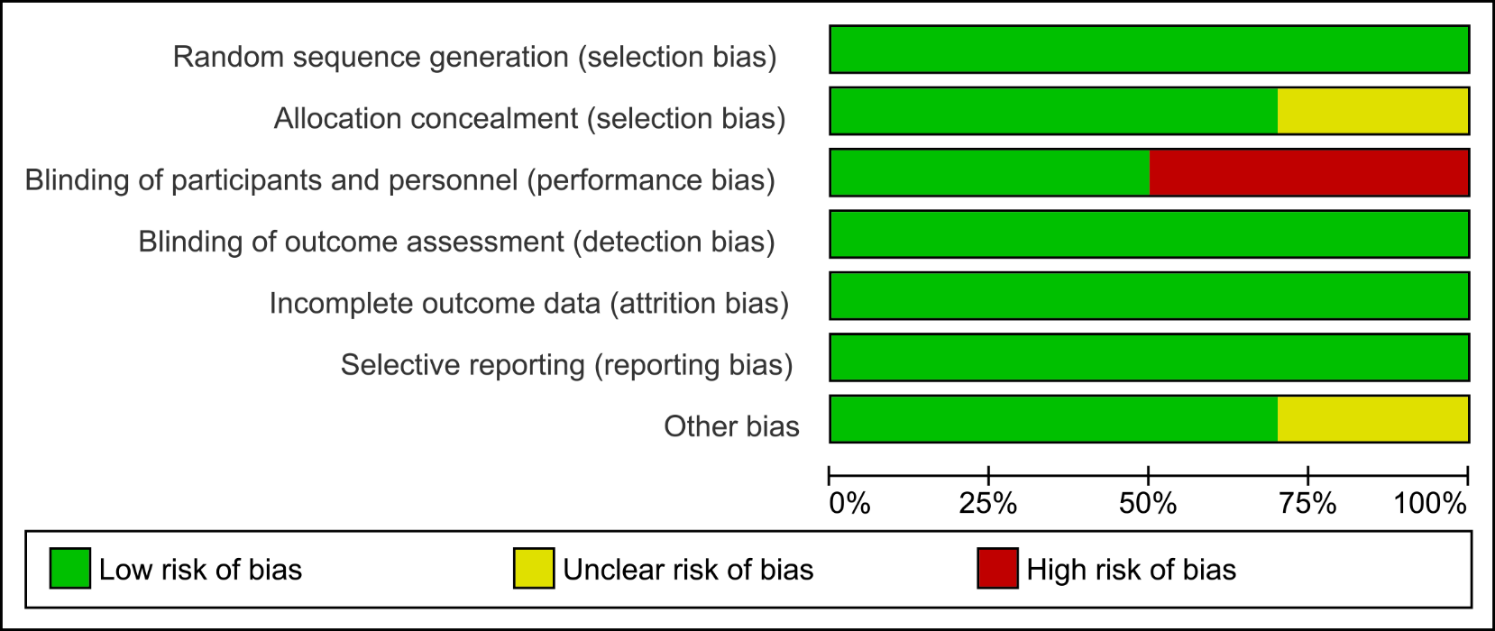


**Supplementary Figure 2. Risk of bias summary in quality assessment for randomized controlled trials.**


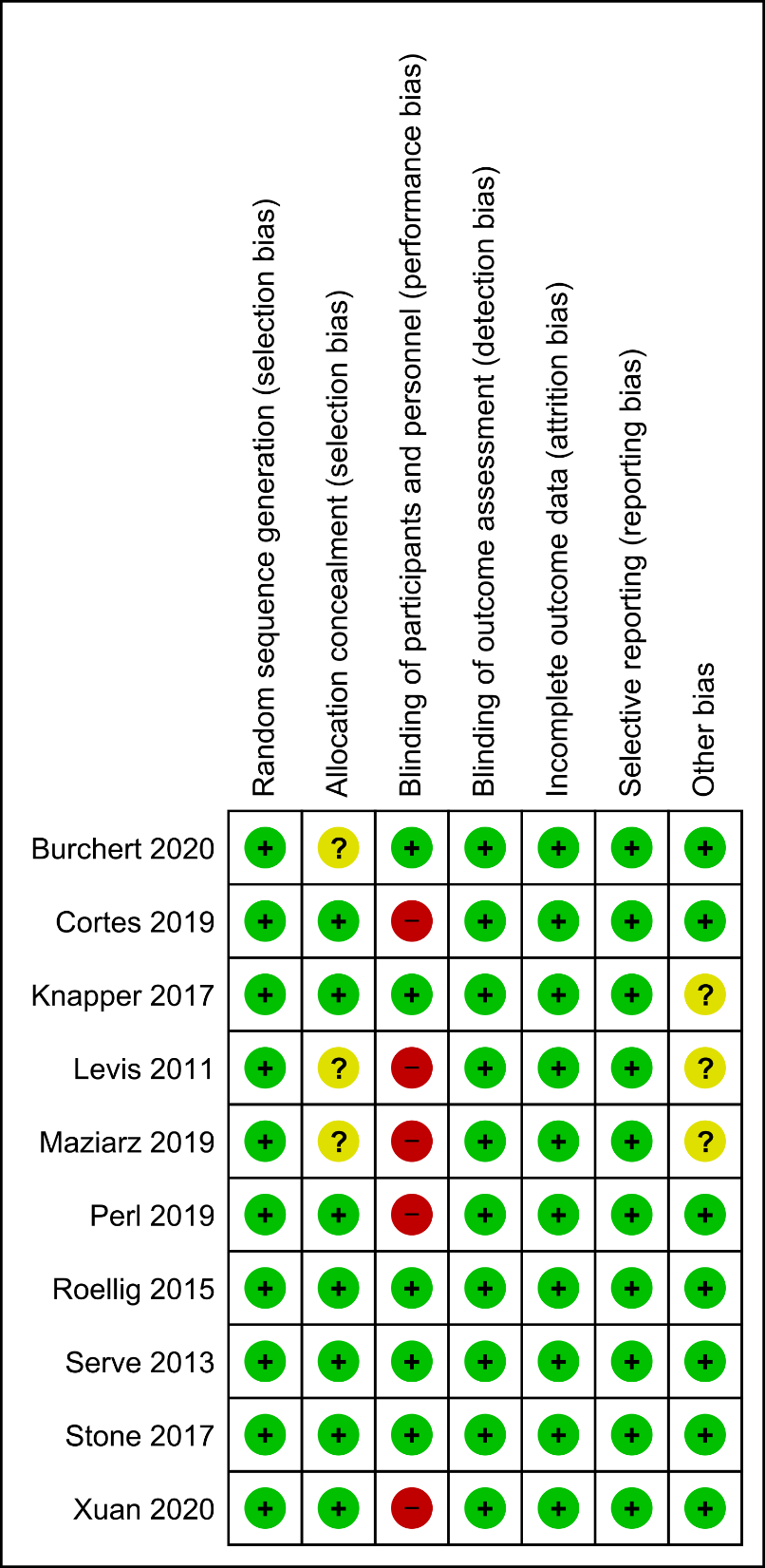


**Supplementary Figure 3. Adjusted HR of EFS and RFS after sensitivity analyses for FLT3i treatment during induction stage in newly diagnosed AML.**


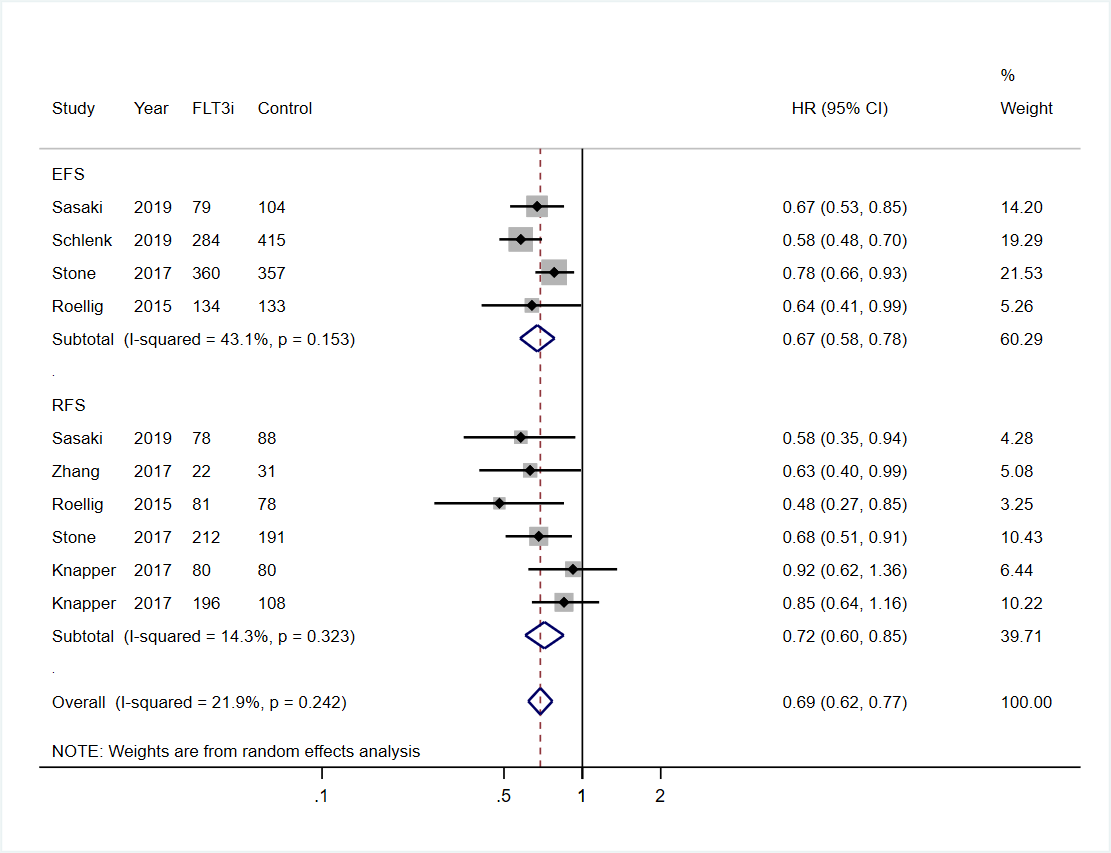


Abbreviations: HR, hazard ratio; 95% CI, 95% confidence interval; EFS, event-free survival; RFS, relapse-free survival; FLT3i, FMS-like tyrosine kinase 3 inhibitor; AML, acute myeloid leukemia.

**Supplementary Figure 4. Adjusted HR of OS and RFS after sensitivity analyses for the role of allo-HSCT in FLT3(+) AML.**


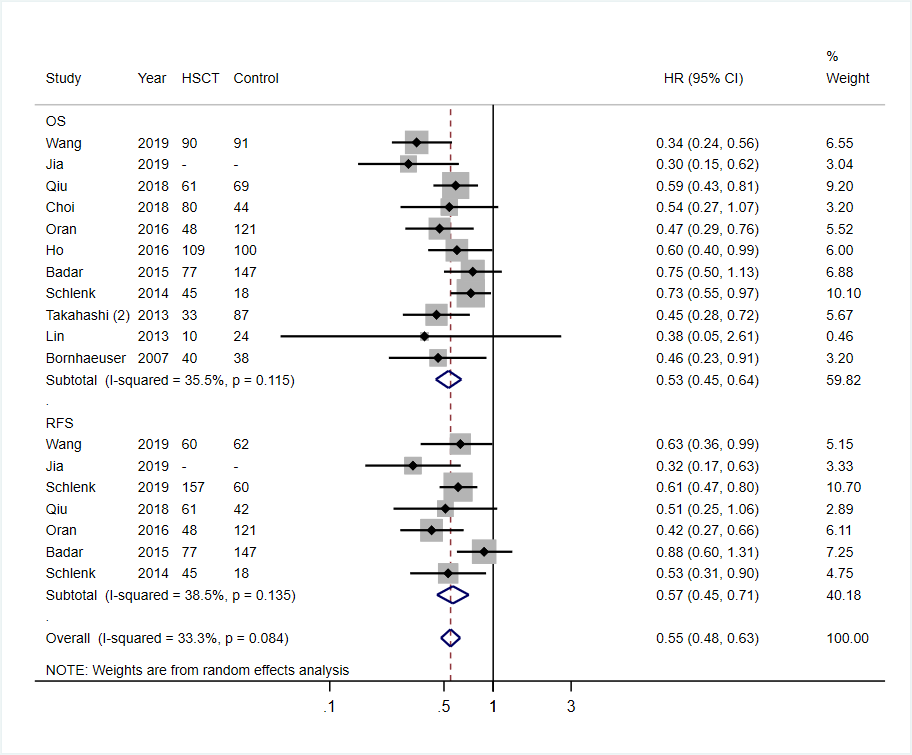


Abbreviations: HR, hazard ratio; 95% CI, 95% confidence interval; OS, overall survival; RFS, relapse-free survival; allo-HSCT, allogeneic hematopoietic stem cell transplant; FLT3, FMS-like tyrosine kinase 3; AML, acute myeloid leukemia.

**Supplementary Figure 5. Adjusted HR of OS after sensitivity analyses for FLT3i treatment in salvage regimen in rrAML.**


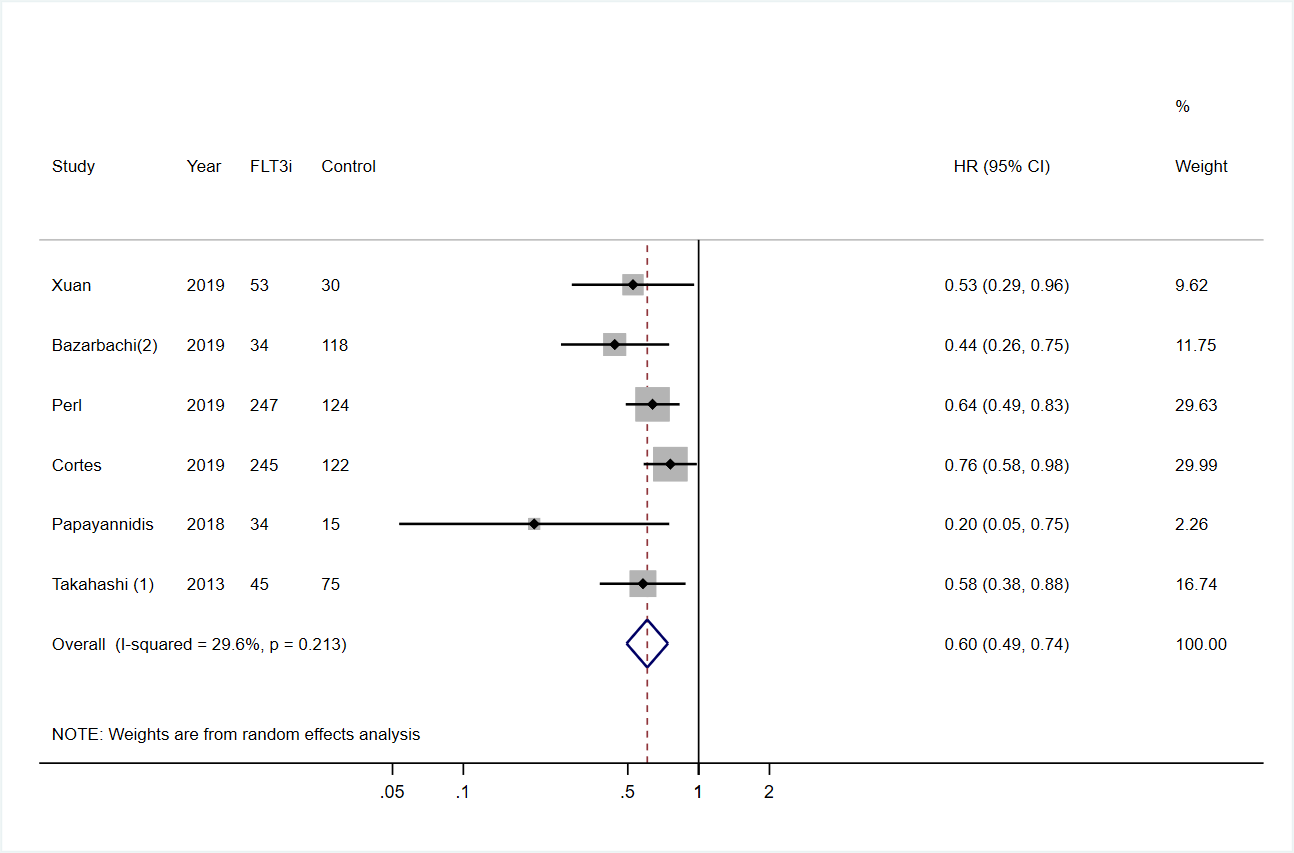


Abbreviations: HR, hazard risk; 95% CI, 95% confidence interval; OS, overall survival; FLT3i, FMS-like tyrosine kinase 3 inhibitor; rrAML, refractory and relapsed acute myeloid leukemia.

**Supplementary Figure 6. Funnel plots for CR.**


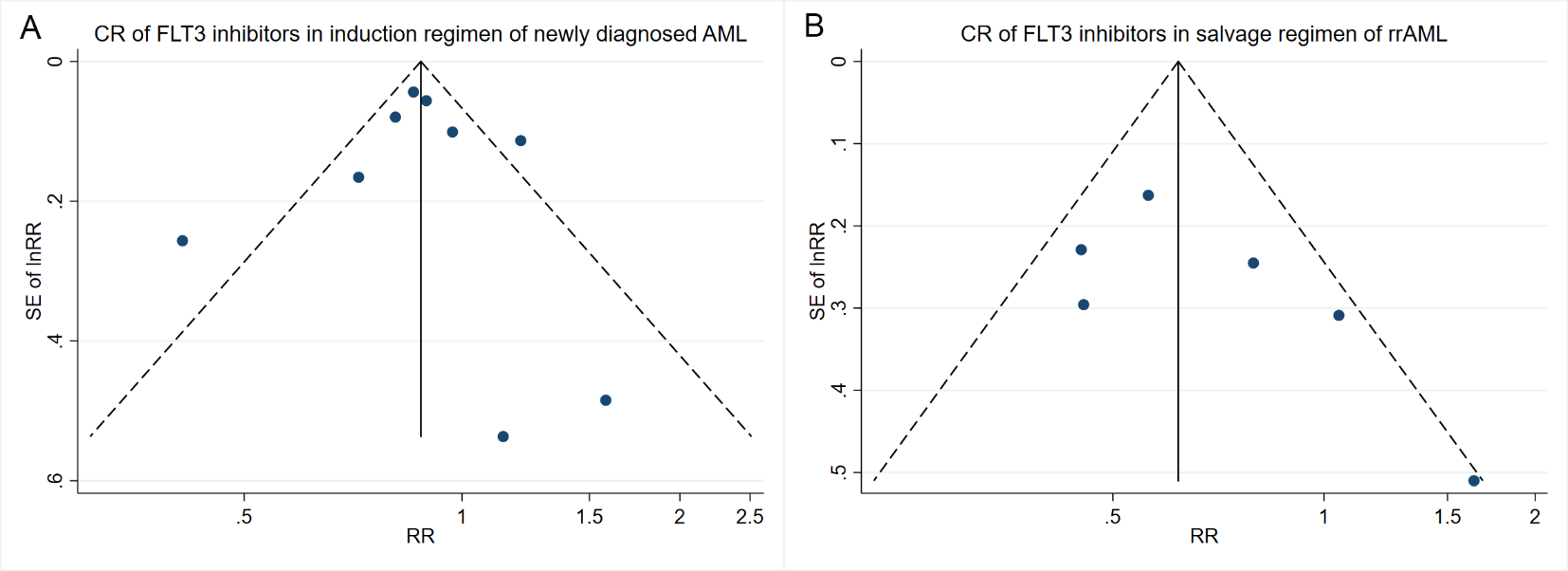


(A) Funnel plot of CR of FLT3i in induction regimen of newly diagnosed AML; (B) Funnel plot of CR of FLT3i in salvage regimen of rrAML. Abbreviations: CR, complete remission; FLT3i, FMS-like tyrosine kinase 3 inhibitor; AML, acute myeloid leukemia; rrAML, refractory and relapsed AML; RR, relative risk; SE, standard error.

**Supplementary Figure 7. Funnel plots for survival outcomes of FLT3i in induction treatment in newly diagnosed AML.**


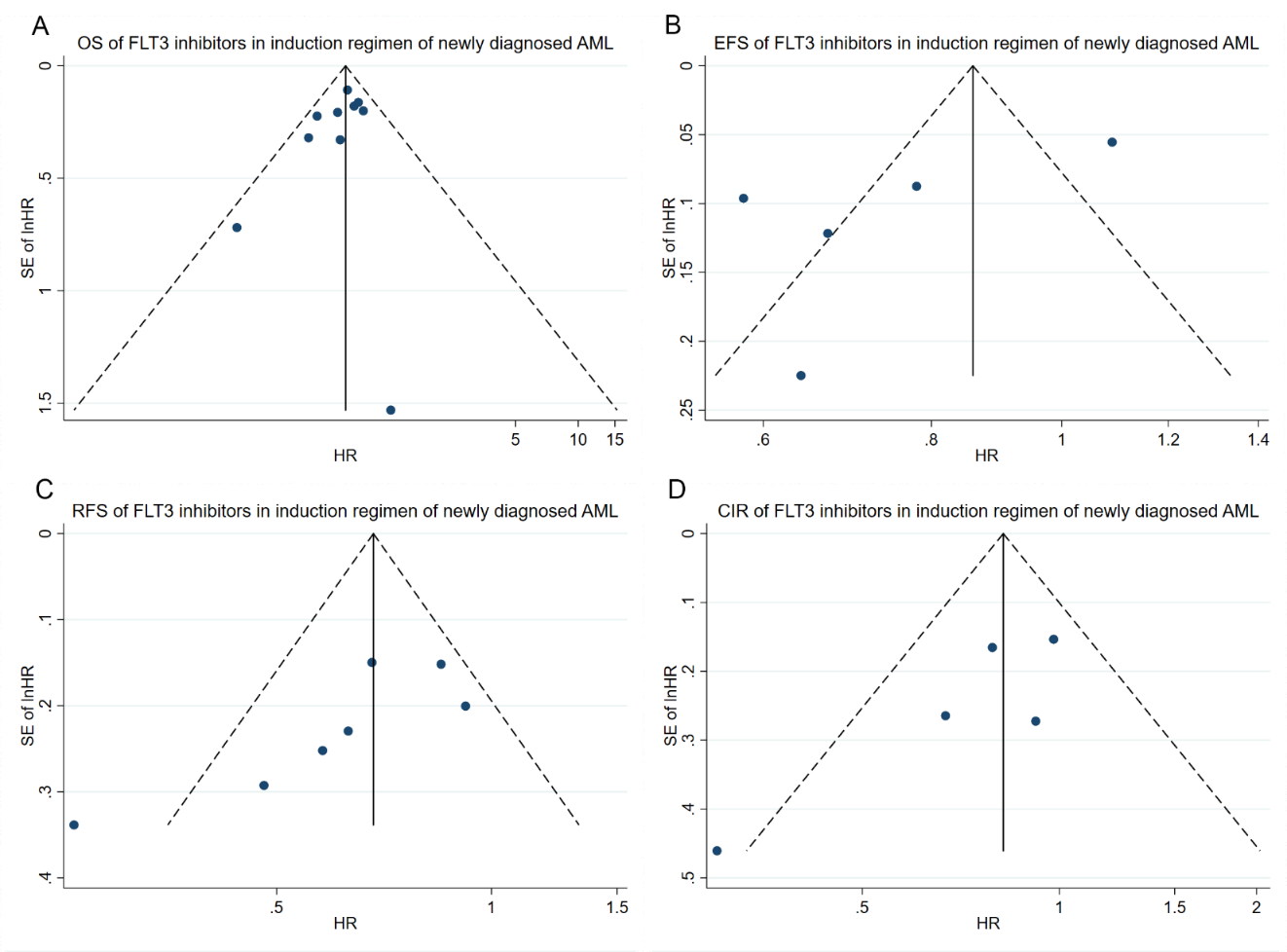


(A) Funnel plot of overall survival; (B) Funnel plot of event-free survival; (C) Funnel plot of relapse-free survival; (D) Funnel plot of cumulative incidence of relapse. Abbreviations: FLT3i, FMS-like tyrosine kinase 3 inhibitor; AML, acute myeloid leukemia; HR, hazard ratio; SE, standard error.

**Supplementary Figure 8. Funnel plots for survival outcomes of allo-HSCT in FLT3(+) AML.**


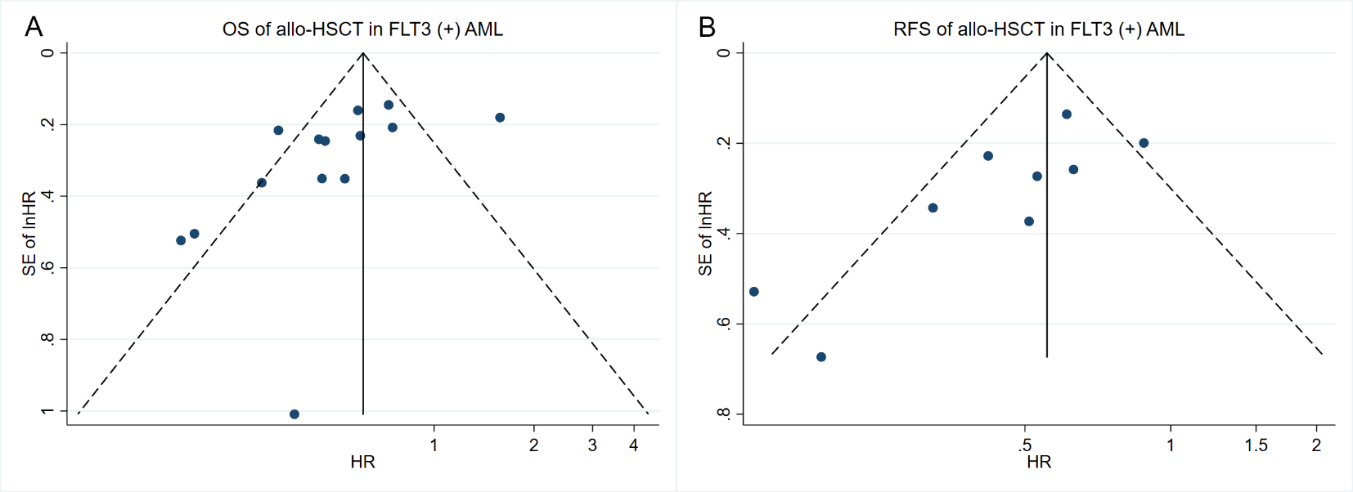


(A) Funnel plot of overall survival; (B) Funnel plot of relapse-free survival. Abbreviations: allo-HSCT, allogeneic hematopoietic stem cell transplant; FLT3, FMS-like tyrosine kinase 3; AML, acute myeloid leukemia; HR, hazard ratio; SE, standard error.

**Supplementary Figure 9. Funnel plots for survival outcomes of FLT3i as maintenance therapy post allo-HSCT.**


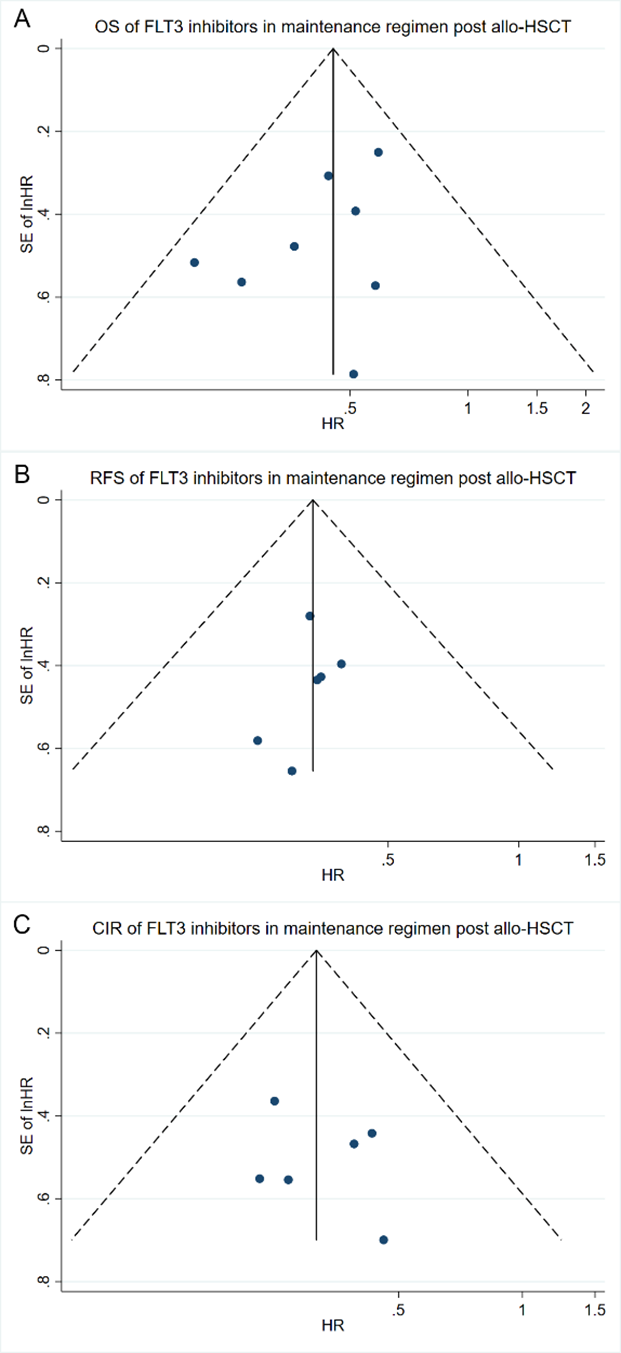


(A) Funnel plot of overall survival; (B) Funnel plot of relapse-free survival; C, Funnel plot of cumulative incidence of relapse. Abbreviations: FLT3i, FMS-like tyrosine kinase 3 inhibitor; allo-HSCT, allogeneic hematopoietic stem cell transplant; SE, standard error.

**Supplementary Figure 10. Funnel plot for OS of FLT3i in salvage regimen for rrAML**.


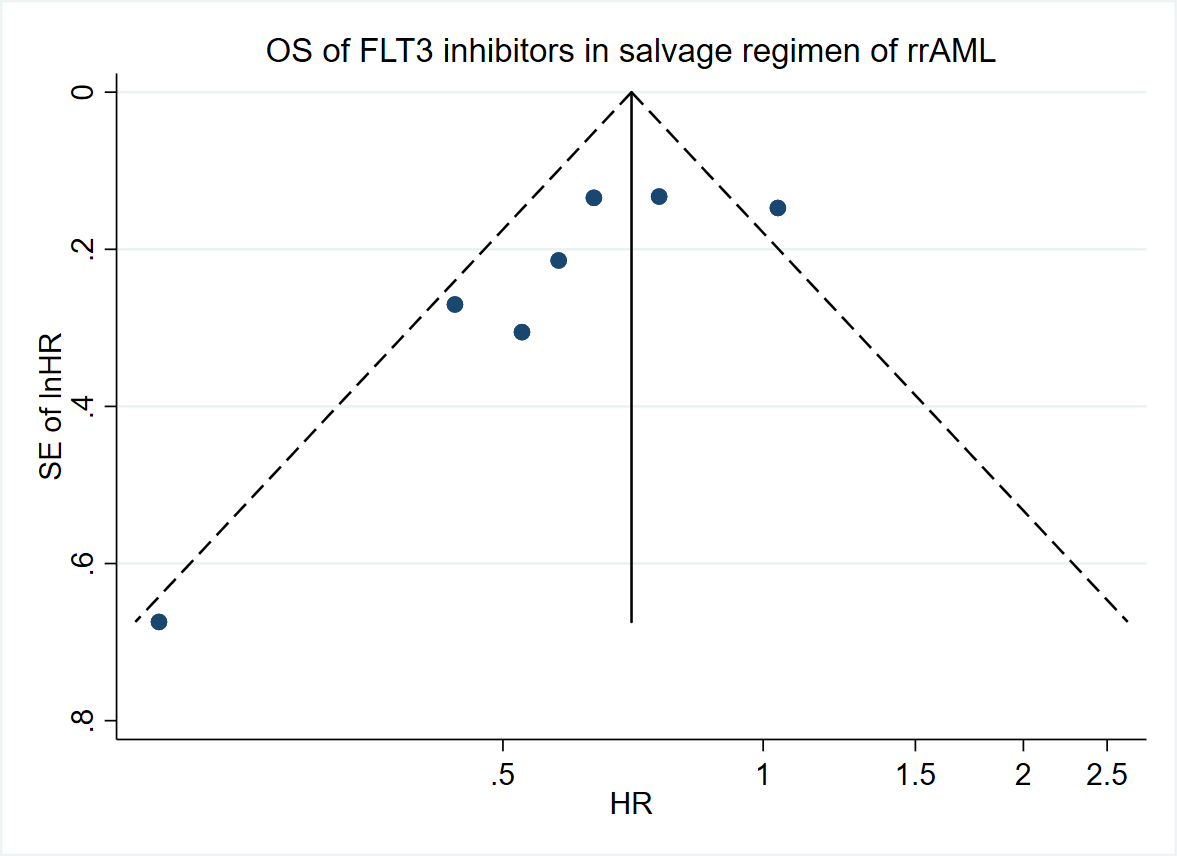


Abbreviations: OS, overall survival; FLT3i, FMS-like tyrosine kinase 3 inhibitor; rrAML, refractory and relapsed acute myeloid leukemia; HR, hazard ratio; SE, standard error.

**Supplementary Figure 11. Network of analyzed comparison in RCT.**


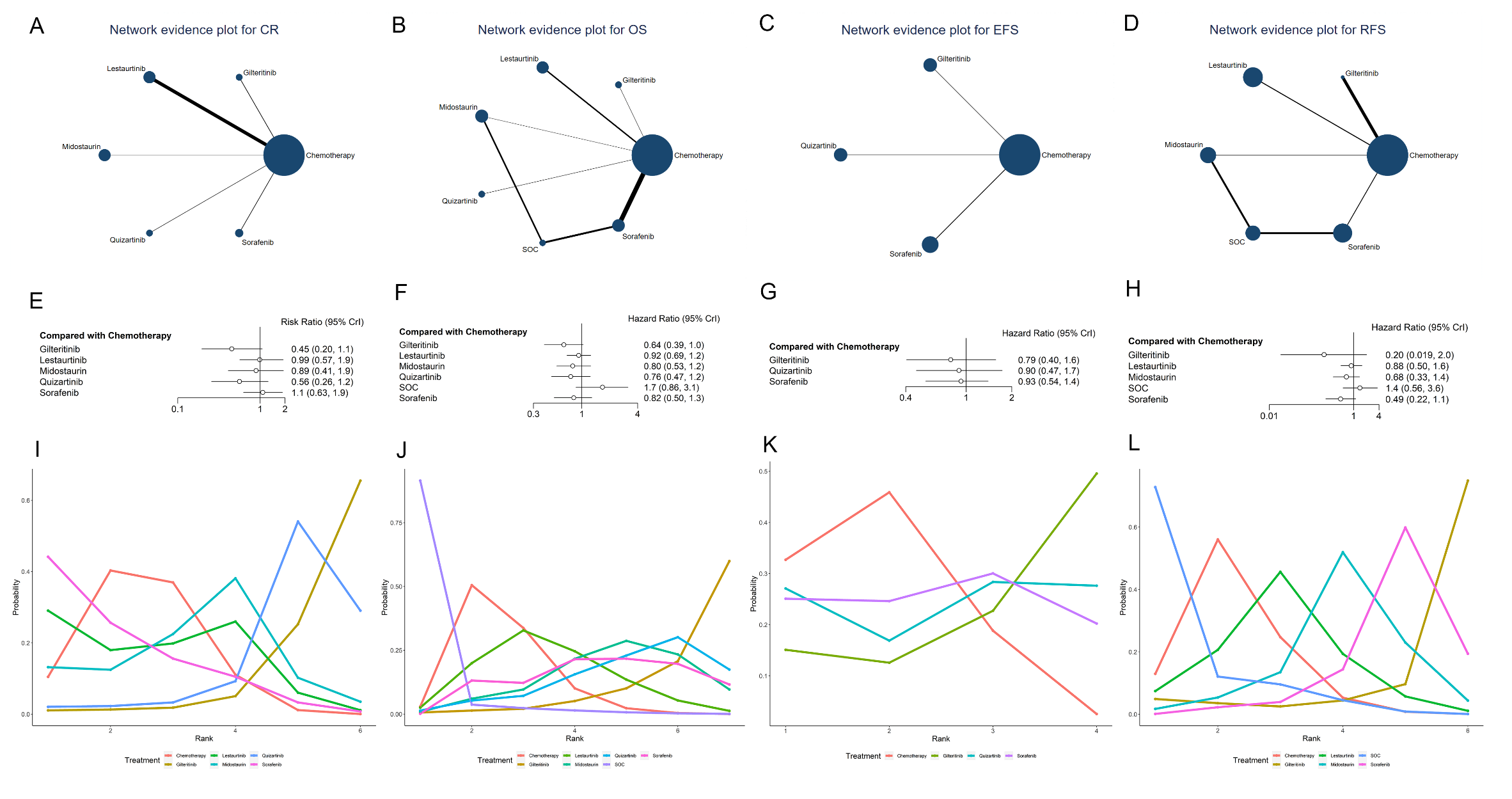


Network evidence plot for CR (A), OS (B), EFS (C), and RFS (D); (E) Comparison of risk ratio for CR between FLT3i and chemotherapy; Comparison of hazard ratio for OS (F), EFS (G), and RFS (H) between FLT3i and chemotherapy; Rank of probability for improving CR (I), OS (J), EFS (K), and RFS (L). Abbreviations: RCT, randomized controlled trial; CR, complete remission; OS, overall survival; EFS, event-free survival; RFS, relapse-free survival; FLT3i, FMS-like tyrosine kinase 3 inhibitor; SOC, standard of care.

**Supplementary Figure 12. Inconsistency between indirect and direct evidence for OS and RFS.**


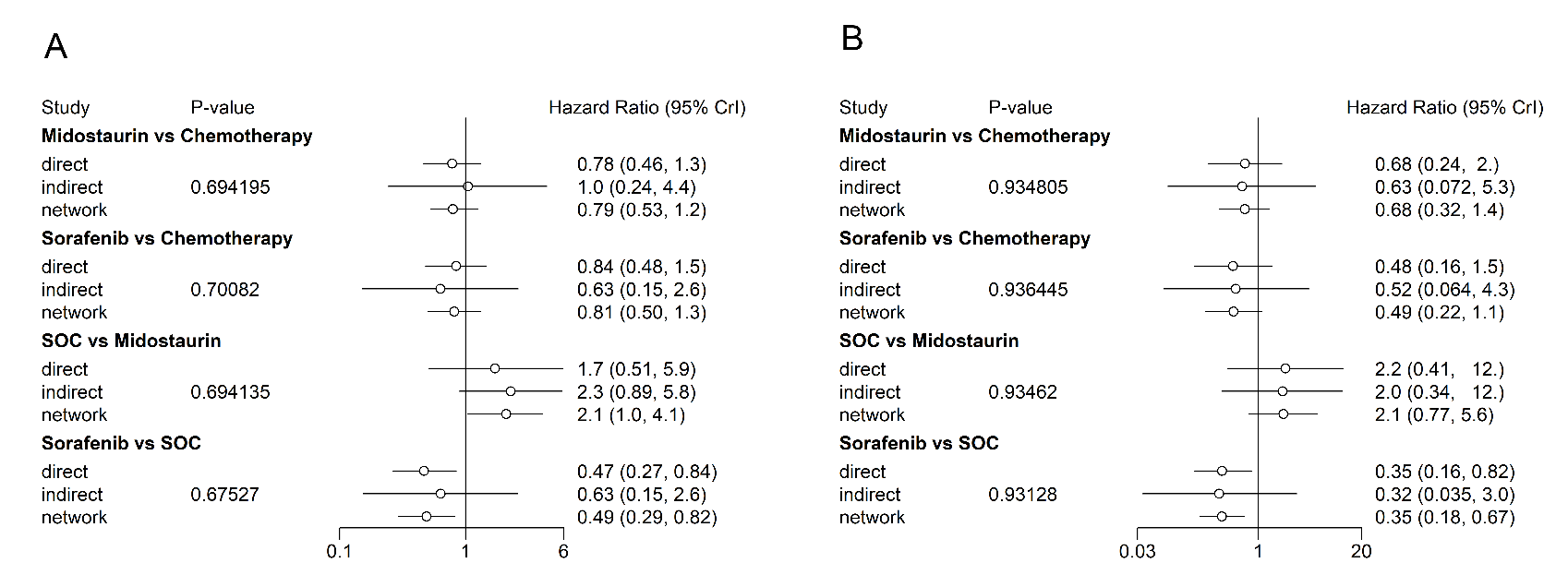


(A) Inconsistency of overall survival; (B) Inconsistency of relapse-free survival. Abbreviations: SOC, standard of care; 95% Crl, credible interval.

**Supplementary Figure 13. Analyses of heterogeneity in endpoints.**


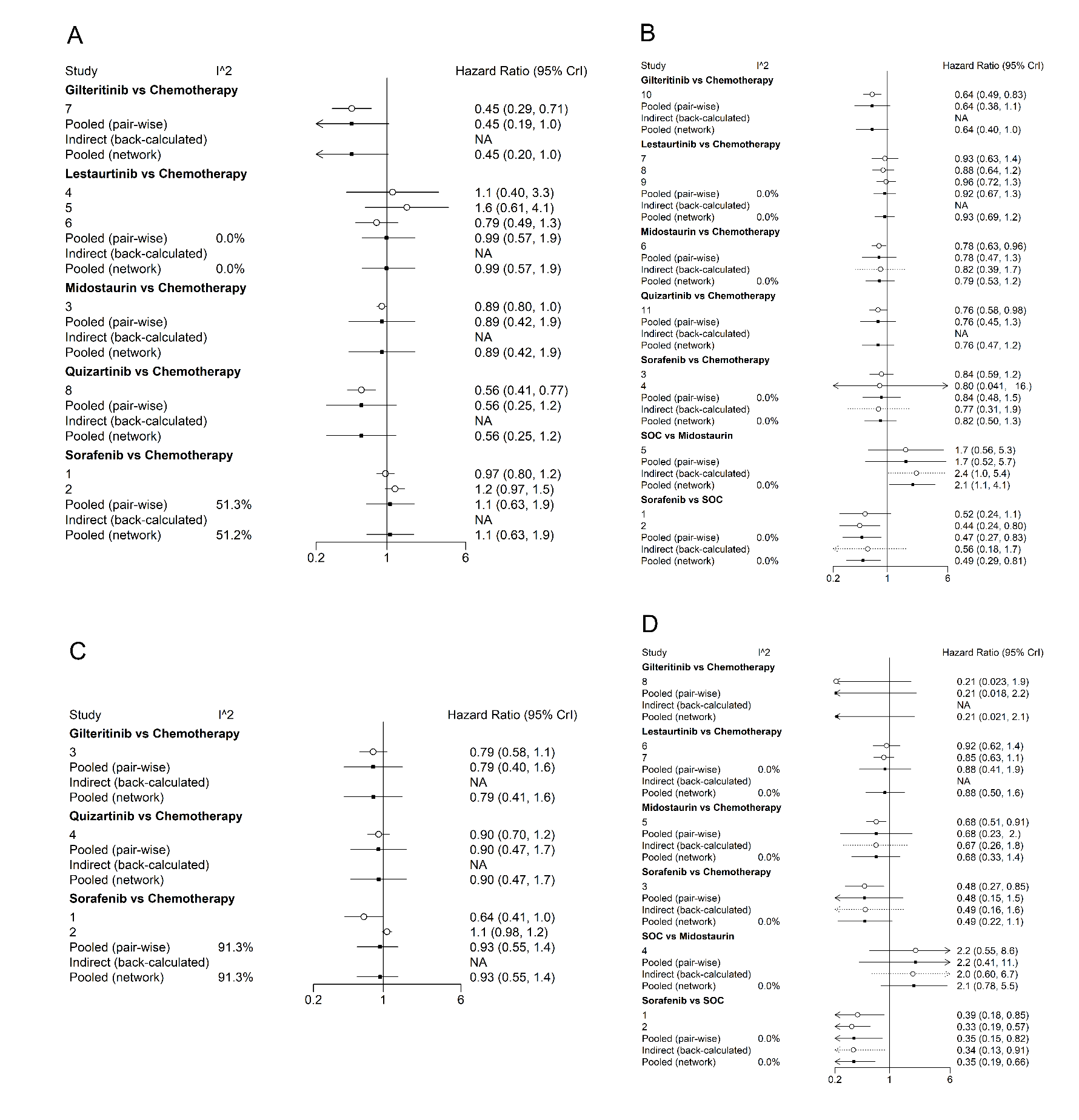


(A) Heterogeneity of complete remission; (B) Heterogeneity of overall survival; (C) Heterogeneity of event-free survival; (D) Heterogeneity of relapse-free survival. Abbreviations: SOC, standard of care; 95% Crl, credible interval.
